# Fat mass and obesity associated protein inhibits neuronal ferroptosis via the FYN/Drp1 axis and alleviate cerebral ischemia/reperfusion injury

**DOI:** 10.1111/cns.14636

**Published:** 2024-03-02

**Authors:** Yi Zhang, Xin Gong

**Affiliations:** ^1^ Department of Emergency, Hunan Provincial People's Hospital The First Affiliated Hospital of Hunan Normal University Changsha Hunan China; ^2^ Department of Neurosurgery, Hunan Provincial People's Hospital The First Affiliated Hospital of Hunan Normal University Changsha Hunan China

**Keywords:** cerebral ischemia/reperfusion, Drp1, ferroptosis, FTO, FYN, mitochondrial fission, N6‐methyladenosine, oxidative stress

## Abstract

**Objectives:**

FTO is known to be involved in cerebral ischemia/reperfusion (I/R) injury. However, its related specific mechanisms during this condition warrant further investigations. This study aimed at exploring the impacts of FTO and the FYN/DRP1 axis on mitochondrial fission, oxidative stress (OS), and ferroptosis in cerebral I/R injury and the underlying mechanisms.

**Methods:**

The cerebral I/R models were established in mice via the temporary middle cerebral artery occlusion/reperfusion (tMCAO/R) and hypoxia/reoxygenation models were induced in mouse hippocampal neurons via oxygen–glucose deprivation/reoxygenation (OGD/R). After the gain‐ and loss‐of‐function assays, related gene expression was detected, along with the examination of mitochondrial fission, OS‐ and ferroptosis‐related marker levels, neuronal degeneration and cerebral infarction, and cell viability and apoptosis. The binding of FTO to FYN, m6A modification levels of FYN, and the interaction between FYN and Drp1 were evaluated.

**Results:**

FTO was downregulated and FYN was upregulated in tMCAO/R mouse models and OGD/R cell models. FTO overexpression inhibited mitochondrial fission, OS, and ferroptosis to suppress cerebral I/R injury in mice, which was reversed by further overexpressing FYN. FTO overexpression also suppressed mitochondrial fission and ferroptosis to increase cell survival and inhibit cell apoptosis in OGD/R cell models, which was aggravated by additionally inhibiting DRP1. FTO overexpression inhibited FYN expression via the m6A modification to inactive Drp1 signaling, thus reducing mitochondrial fission and ferroptosis and enhancing cell viability in cells.

**Conclusions:**

FTO overexpression suppressed FYN expression through m6A modification, thereby subduing Drp1 activity and relieving cerebral I/R injury.

## Graphical abstract

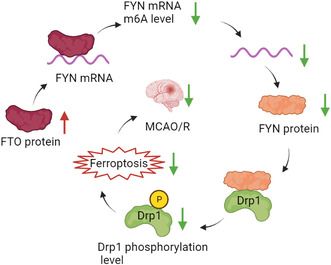

## 1 INTRODUCTION

Cerebral ischemia/reperfusion (I/R) injury is devastating neuronal injury resulting from the blood reperfusion after arterial occlusion‐causing ischemic stroke and neurological or cardiovascular surgeries.^1^ It is characterized by neuronal death and can cause cognitive decline, disability, and even mortality.^2^ Current available treatments (mechanical therapies and drug therapies) for cerebral I/R injury are often accompanied by side effects. Therefore, there is a need to discover new strategies to treat cerebral I/R injury. During this injury, oxidative stress (OS) and ferroptosis are important mechanisms: cerebral I/R injury can induce OS and ferroptosis; OS is related to the pathogenesis of cerebral I/R injury; both the inhibition of OS and ferroptosis are revealed to protect neurons against this injury.^3^, ^4^, ^5^ To deepen the understanding of the detailed mechanisms in cerebral I/R injury, more experimental data are needed to be acquired through a mass of in‐depth research.

A healthy network of mitochondria is crucial for cardiovascular function, during which the dynamic changes of mitochondrial fission and fusion are important, and the aberrant mitochondrial fission can cause the development of diseases.^6^ Increasing documentations have stated the involvement of mitochondrial fission alternation in the molecular research of cerebral I/R injury.^7^, ^8^ Dynamin‐related protein 1 (Drp1) is a vital pro‐fission protein that manipulates mitochondrial dynamics and complex processes of cardiac and organ changes.^9^ Notably, it has been reported to be elevated after cerebral I/R injury and accelerates the formation of autophagosomes and inflammation and damages mitochondria.^10^ In myocardial I/R, Drp1‐Ser616 phosphorylation induces ROS accumulation and cardiomyocyte necrosis.^11^ Src‐like protein‐tyrosine kinase Fyn (FYN) activated Drp1 signaling in an intracerebral hemorrhage model.^12^ Belonging to the Src nonreceptor tyrosine kinase family, FYN exerts many functions in cellular processes and is related to inflammation in the central nervous system.^13^ Importantly, FYN stimulates neuroinflammation and OS, thus irritating I/R injury.^14^ However, the function of the FYN/Drp1 axis on ferroptosis in cerebral I/R injury warrants further investigations.

N6‐methyladenosine (m6A) modification is an epigenetic modification taking a part in several pathophysiological processes, which is revealed to be associated with the progression of neurological diseases together with its related enzymes.^15^ Several articles have exposed the roles of m6A modification in cerebral I/R injury,^16^, ^17^ which indicates the likely participation of m6A‐associated enzymes. The fat mass and obesity‐associated (FTO) protein is a recognized m6A demethylase related to the development of many diseases via the modulation of m6A modification.^18^, ^19^, ^20^ Importantly, FTO has been stated to modulate neurogenesis and participate in the progression of many neurological diseases including cerebral I/R injury.^21^, ^22^, ^23^, ^24^ Meanwhile, the mRNA of FYN was capable of being stabilized through the m6A modification in hepatocellular carcinoma.^25^ Our bioinformatics analysis uncovered the probability of FTO in mediating m6A modification of FYN. These manifested the potential regulatory mechanism of these two proteins in cerebral I/R injury.

As a result, we conjectured that FTO and the FYN/Drp1 axis have impacts on mitochondrial fission, OS, and ferroptosis in cerebral I/R injury and there are interactions between these proteins. To prove this conjecture, we designed a set of experiments in cellular and animal models, aiming to enrich the knowledge of the molecular mechanisms underlying cerebral I/R injury.

## 2 MATERIALS AND METHODS

### 2.1 Establishment of temporary middle cerebral artery occlusion/reperfusion (tMCAO/R) models

Totally 90 adult male C57BL/6 mice were obtained from Hunan SJA laboratory animal Co., Ltd (Changsha, China) to establish the tMCAO/R models. All animal experiments were ratified by Ethics Committee of Hunan Provincial People's Hospital, and carried out according to the standards formulated by the Animal Experiment Committee of Hunan Provincial People's Hospital and Guidelines for the Care and Use of Laboratory Animals released by National Institutes of Health.

The tMCAO/R model was established via 60‐min MCAO in the right brain and subsequent reperfusion as per previous research^26^: the mouse was anesthetized, after which the skin in the neck was cut open, a single line was inserted into internal carotid artery (ICA) along the distal end of the external carotid artery (ECA); when the line reached MCA, the artery was occluded for 60 min and the line was withdrawn. The experimental animals were covered with a thermostatic blanket to keep a constant temperature. The sham group of mice underwent the operation without artery occlusion. All mice were assigned into sham (receiving sham operation), MCAO/R (receiving cerebral I/R), MCAO/R + overexpression (oe)‐negative control (NC) (receiving simultaneous cerebral I/R and NC overexpression), MCAO/R + oe‐FTO (receiving simultaneous cerebral I/R and FTO overexpression), MCAO/R + oe‐FTO + oe‐NC (receiving simultaneous cerebral I/R and FTO and NC overexpression), MCAO/R + oe‐FTO + oe‐FYN (receiving simultaneous cerebral I/R and FTO and FYN overexpression), MCAO/R + oe‐NC + DMSO (receiving cerebral I/R, NC overexpression, and DMSO treatments), MCAO/R + oe‐FTO + DMSO (receiving cerebral I/R, FTO overexpression, and DMSO treatments), and MCAO/R + oe‐FTO + Mdivi‐1 (receiving cerebral I/R, FTO overexpression, and Drp1 inhibitor Mdivi‐1 treatments) groups (10 mice each group). At the time of reperfusion after 60‐min ischemia, overexpression‐related lentivirus vectors (total of 5 μL, viral titer: 4–8 × 10^8^ TU/mL) were injected locally at 1 μL/min with a 10 ‐μL microinjector using a mouse brain stereotaxic instrument (Stoelting, Chicago, Illinois, USA). The specific injection location was AP: +0.3 mm, 0.8 mm, and 1.9 mm; ML: 2.5 mm; DV: −2 mm.^27^ After injection, the microinjector stayed unmoved for 5 min. The following experiments were carried out 24 h after reperfusion. Lentivirus particles were purchased from Genomeditech (Shanghai, China).

### 2.2 Fluoro‐Jade C (FJC) staining

The brain tissue sections were dried for 24 h at 60°C, eluted by gradient ethanol, and soaked in 0.06% potassium, followed by 10‐min incubation in 0.1% acetic acid dissolved in 0.0001% FJC (EMD Millipore, Billerica, MA, USA). Afterward, brain tissue sections were baked for 10 min at 50°C and coated with neutral balsam, followed by the observation of degenerated neurons under a fluorescence microscope.

### 2.3 2,3,5‐Triphenyltetrazolium chloride (TTC)‐staining

Five mice were selected from each group and brain tissues were cut into 5 pieces of 2 mm‐thick coronal sections and stained for 30 min in 2% TTC (G3004, Solarbio, Beijing, China) solution at 37°C. The infarction zone presented pale and noninfarction zone presented brick red, and both regions were analyzed with Image J software. Considering the limitation of edema, the formula for calculating infarction volume was corrected according to Swanson's method:

$$\begin{array}{c} \text{Infarction volume}\;\left(\%\right)=\left(\text{Ischemia contralateral volume}–\text{Non}\right.\\ \left.-\text{infarct volume}\;\mathrm{on}\;\text{the ischemic side}\right)/\\ \text{Ischemia contralateral volume}\times 100\%. \end{array}$$

### 2.4 Detection of brain water content

The dry‐wet method was adopted for the detection of edema. The brain tissue weight was the wet weight and the weight of the brain tissue after 24‐h baking at 95°C was the dry weight. The brain water content = (wet weight – dry weight)/wet weight × 100%.

### 2.5 Transmission electron microscopy (TEM) observation of mitochondrial ultrastructure

Brain tissues 1 mm^3^ around the brain infarction were taken and fixed in 2.5% glutaraldehyde, followed by 0.1 M phosphate‐buffered saline (PBS) washing. Subsequently, samples were dehydrated in 50%, 70%, 90%, and 100% ethanol and 100% epoxypropane, after which they were embedded in epoxypropane + embedding solution (2:1) and made into 1 μm sections with an ultramicrotome. Next, sections were stained with 3% uranyl acetate lead citrate and monitored with a TEM (HT7700, Hitachi, Tokyo, Japan). And mitochondrial length was measured using the standard EMIP‐SP software.

### 2.6 Detection of mitochondrial membrane potential (ΔΨm)

ΔΨm was detected with JC‐1 kits (ab141387, Abcam, Cambridge, UK): after the related treatment as per the instructions, samples were cultured with JC‐1 dying liquid for 20 min avoiding light, followed by fluorescence microscope examination.

### 2.7 Enzyme‐linked immunosorbent assay (ELISA)

The expression of malondialdehyde (MDA, S0131S), glutathione (GSH, S0052), superoxide dismutase (SOD, S0101S) was monitored in accordance with the instructions of kits (Beyotime, Shanghai, China). A Benchmark microplate reader (Bio‐Rad Laboratories, Hercules, CA, USA) was used to measure optical density (OD).

### 2.8 Fe^2+^ content detection

The treated tissues or cells were centrifuged and 220 μL supernatants were mixed with 60 μL Fe^2+^ detection reagents and reacted for 30 min, after which the OD values were analyzed at 562 nm and the content of Fe^2+^ was calculated through the comparison with the standard curve.

### 2.9 Cell culture and establishment of oxygen–glucose deprivation/reoxygenation (OGD/R) cell models

Mouse hippocampal neurons HT22 were purchased from iCell Bioscience (Shanghai, China) for the establishment of I/R models to simulate the cerebral I/R injuries in vivo. Cells were cultured in a Dulbecco's modified Eagle medium (DMEM) involving 10% fetal bovine serum and 1% penicillin–streptomycin solution at 37°C with 5% CO_2_ and 95% air. For the establishment of OGD/R models, HT22 cells were treated with hypoxia for 6 h in a glucose‐free medium at 37°C. The specific operations were as follows: 1 × 10^4^ HT22 cells were seeded in 96‐well plates in a normal medium until 80% confluence, after which cells were treated with a glucose‐free medium and then put in a sealed container (Mitsubishi Gas Chemical Company, Tokyo, Japan) containing anaerobes. After 6‐h OGD, cells were cultured in a normal medium in a normoxia environment to create the condition of OGD/R. Cells were arranged into control, OGD/R [treated with hypoxia/reoxygenation (H/R)], OGD/R + oe‐NC (treated with H/R and NC overexpression), OGD/R + oe‐FTO (treated with H/R and FTO overexpression), OGD/R + oe‐NC + DMSO (treated with H/R, NC overexpression, and DMSO), OGD/R + oe‐FTO + DMSO (treated with H/R, FTO overexpression, and DMSO), and OGD/R + oe‐FTO + Mdivi‐1 [treated with H/R, FTO overexpression, and Drp1 inhibitor Mdivi‐1 (10 μM, Beyotime)] groups. The overexpression vectors and its negative lentivirus recombinant vectors were designed according to the experiment design requirements, and the lentivirus transfection reagents were used to cotransfect into HEK293T cells for 48 h, after which p24 ELISA kits (Cell Biolabs, San Diego, CA, USA) were utilized for measurement of virus titer. After that, cell density was adjusted to 1 × 10^5^/mL and 200 μL/well cell suspension was seeded in the 24‐well plates overnight. The next day, the prepared lentivirus particles were utilized for 24‐h cell infection and 5 μg/mL Polybrene (GM‐040901, Genomeditech) was used to enhance the infection efficiency. Lentivirus usage volume = MOI * cell count/virus titer (multiplicity of infection [MOI] = 10).

### 2.10 Cell counting kit (CCK)‐8 assay

The cell counting kits (CK04, Dojindo, Kumamoto, Japan) were used to test the cell viability via the CCK‐8 assay. Cells at the logarithmic growth phase were seeded in 96‐well plates at 1 × 10^4^ cells/well and cultured for 24 h, after which they were treated as per grouping. Forty‐eight h after the treatment, cells were incubated with 10 ‐μL CCK‐8 reagents for 3 h at 37°C and the OD values were detected at 450 nm on a microplate reader to analyze the cell viability.

### 2.11 TdT‐mediated dUTP‐biotin nick end‐labeling (TUNEL) assay

Logarithmically growing cells were seeded in the slides in 6‐well plates at 1 × 10^6^/mL. TUNEL kits (Abbkine Scientific, Wuhan, Hubei, China) were adopted for TUNEL apoptosis detection. Cell suspensions were coated on a slide and dropwise added with 4% paraformaldehyde for 1‐h fixing. Subsequently, the fixed cells were treated for 3 min with 0.1% Triton X‐100 (Beyotime) at 4°C and cultured for 1 h with 50 μL TUNEL solution in a darkroom at 37°C, followed by 3 washings with PBS. Samples were then mounted with antifluorescence quenching mounting liquid, and the fluorescence density was observed under a fluorescence microscope. Last, 5 high‐power visual fields were randomly selected to count the TUNEL‐positive cells [apoptotic rate = the number of apoptotic cells (red)/the number of total cells (blue) * 100%].

### 2.12 Flow cytometry

Annexin V‐FITC/PI Apoptosis Detection Kit (556547, BD Biosciences) was utilized to detect cell apoptosis. Briefly, 100 μL cells (concentration 1 × 10^7^/mL) were taken from each tube and tested using flow cytometry (BD FACSCAnto™II, BD Biosciences), FlowJo version 7.6.1 software was used for data analysis. The apoptosis rate = Annexin V‐FITC+/PI−(early apoptosis, Q3) + Annexin V‐FITC+/PI+(late apoptosis, Q2).

### 2.13 Adenosine Triphosphate (ATP) synthesis detection

Cells (1 × 10^4^) were incubated with 200 μL cell lysis buffers according to the manuals of ATP detection kits (Beyotime), titrated from top to bottom, and centrifuged for 5 min at 1200 × *g*. Afterward, the supernatants were transferred to the prepared wells for 5‐min incubation at room temperature, followed by the determination of ATP fluorescence with the microplate reader.

### 2.14 ROS level detection

The generation of mitochondrial ROS was measured with MitoSox Red (Thermo Fisher Scientific Inc., Waltham, MA, USA) (red fluorescence). Finally, a laser confocal microscope system (LSM 710, Carl Zeiss, Jena, Germany) was utilized to collect images at a 510 nm‐excitation wavelength and a 590 nm‐emission wavelength, and the ImageJ software was used to calculate the average fluorescence density.

### 2.15 Western blot

Tissue/Cell Mitochondria Separation kit (Yeasen, Shanghai, China) was used to separate mitochondria from tissues or cells. Enhanced Radio‐Immunoprecipitation assay cell lysis buffers containing trypsin inhibitor (Boster, Wuhan, Hubei, China) were used to extract proteins from cells, tissues, or mitochondria, and the concentration of protein was determined with bicinchoninic acid protein quantification kits (Boster). Proteins were then separated in 10% sodium dodecyl sulfate polyacrylamide gel electrophoresis (SDS‐PAGE) and then transferred to a polyvinylidene fluoride membrane. Subsequent to 2‐h blocking in 5% bovine serum albumin to block the nonspecific binding at ambient temperature, membranes were probed overnight at 4°C with primary antibodies against glutathione peroxidase (GPX4, ab125066, 1:1000, Abcam), 4‐hydroxy‐2‐nonenal (4HNE, ab243070, 1:1000, Abcam), FTO (ab280081, 1:1000, Abcam), FYN (ab184276, 1:1000, Abcam), Drp1 (ab184247, 1:1000, Abcam), p‐Drp1 Ser616 (PA5‐64821, 1:200, Thermo Fisher Scientific Inc.), COX IV (ab202554, 1:2000, Abcam), and β‐actin (ab8226, 1:4000, Abcam). After washing, membranes were incubated for 1 h with horseradish peroxidase‐labeled goat‐anti‐rabbit or goat‐anti‐mouse secondary antibody (ab6721, ab6789, 1:2000, Abcam) at room temperature. Membranes were then cultured for 1 min with electrochemiluminescence (ECL) working liquid (EMD Millipore) at ambient temperature, after which the redundant ECL working liquid was removed. Membranes were then sealed with preservative film and exposed to an X‐ray film in a dark box for 5–10 min, followed by developing and fixation. Image J software was adopted for the measurement of gray levels of western blot bands. β‐actin was utilized as the internal reference for total protein and COX IV for mitochondrial protein.

### 2.16 Methylated RNA immunoprecipitation (Me‐RIP)

The Trizol method was adopted to extract the total RNA from brain tissues and PolyATtract® mRNA Isolation Systems (A‐Z5300, A&D Technology, Beijing, China) to isolate and purify the mRNA from total RNA. As per grouping, the anti‐m6A antibody (1:500, ab208577, Abcam) or anti‐IgG antibody (ab6789, 1:100) (Abcam) was probed for 1 h with A/G magnetic beads for binding in IP buffers [20‐mM Tris (pH 7.5), 140‐mM NaCl, 1% NP‐40, and 2‐mM EDTA]. The isolated and purified mRNA and magnetic bead‐antibody complexes were added to IP buffers involving RNAase inhibitor and protease inhibitor for overnight incubation at 4°C. After elution, RNA was extracted and purified by phenol‐chloroform, followed by reverse transcription‐quantitative polymerase chain reaction (RT‐qPCR) analysis of FYN.

### 2.17 Photo‐activatable ribonucleoside‐enhanced crosslinking and immunoprecipitation (PAR‐CLIP)

After lysing, brain tissues were cultured for 14 h in 200 μM 4‐thiopyridine (Sigma‐Aldrich, St. Louis, MO, USA) and cross‐linked with 0.4 J/cm^2^ at 365 nm. Subsequent to lysing, FTO antibody was treated for IP overnight at 4°C. [γ‐‐32‐P]‐ATP was used to label the precipitated RNA, which was then observed with autoradiography. The precipitates underwent protease K treatment to remove the protein, followed by the isolation of RNA with Trizol and RT‐qPCR detection of FYN levels.

### 2.18 FYN mRNA stability assay

HT22 cells 2 × 10^5^ per well were seeded in 6‐well plates and treated with actinomycin D (2 μg/mL, Sigma‐Aldrich) for 0, 2, 4, and 8 h. RT‐qPCR experiment detected the expression of remaining FYN mRNA at various time points to evaluate the degradation of FYN mRNA.

### 2.19 Co‐immunoprecipitation (Co‐IP)

Tissues were lysed in Co‐IP buffers, followed by centrifugation and clarifying through 1.5‐h incubation with 25 μL protein A/G agarose at 4°C. At the same temperature, FYN antibody or p‐Drp1 and Drp1 antibodies were cultured in pre‐clarified supernatants for overnight IP. Subsequently, the protein complexes were obtained through 2‐h incubation with 30 μL protein A/G gel at 4°C and then isolated with SDS‐PAGE, followed by western blot analysis.

### 2.20 RT‐qPCR

The RT of total RNA was performed with the RT kits. A fluorescence quantitation PCR instrument was utilized to monitor the expression of genes with reaction conditions as per manuals of the fluorescence quantitation PCR kits (Yesen Biotechnology, Shanghai, China). With β‐actin as the internal reference, the $2^{-\Delta \Delta C_{t}}$ method was employed for data analyses (Table 1).

**TABLE 1:** Primer sequences.

Name of primers	Sequences
FYN‐F	TCGTGGCAAAAGAGCTTGGA
FYN‐R	GCTTCCCACCAGTCTCCTTC
β‐Actin‐F	TGAGCTGCGTTTTACACCCT
β‐Actin‐R	TTTGGGGGATGTTTGCTCCA

Abbreviations: F, forward; R, reverse.

### 2.21 Immunofluorescence

Tissue sections were dewaxed and placed in citric acid buffers, permeated with 0.3% Triton X100, and then cultured in QuickBlock™ immunofluorescence blocking solution, followed by overnight incubation with anti‐FYN and anti‐p‐Drp1 antibodies. Following 3 washes of PBS, samples were probed for 1 h with a secondary antibody, after which they were counterstained for 20 min with 4′,6‐diamidino‐2‐phenylindole in dark at ambient temperature. Subsequent to sealing with the antifluorescence quenching reagent, a fluorescence microscope (Olympus, Tokyo, Japan) was adopted to visualize protein expression, and ImageJ software was used to calculate the positive cells in microscope images, with five randomly selected visual fields (×200).

### 2.22 Statistical analysis

SPSS21.0 (IBM‐SPSS Inc., Chicago, IL, USA) was applied for analyses, and measurement data were depicted as mean ± standard deviation. For cell experiments, the sample size was 3, and data were tested using nonparametric tests. The Mann–Whitney test was used for comparisons between two groups, and Kruskal–Wallis for those among multiple groups. For animal experiments, the sample size was five, and Shapiro–Wilk test was used to test the normality of the data. For samples conforming to normal distribution, the dependent sample *t*‐test was used for comparisons between two groups and one‐way ANOVA for those among multiple groups. Samples that do not conform to the normal distribution are tested by nonparametric tests. *p* < 0.05 was regarded as significantly different.

## 3 RESULTS

### 3.1 FTO overexpression inhibited mitochondrial fission and MCAO/R cerebral injury

**FIGURE 1:**
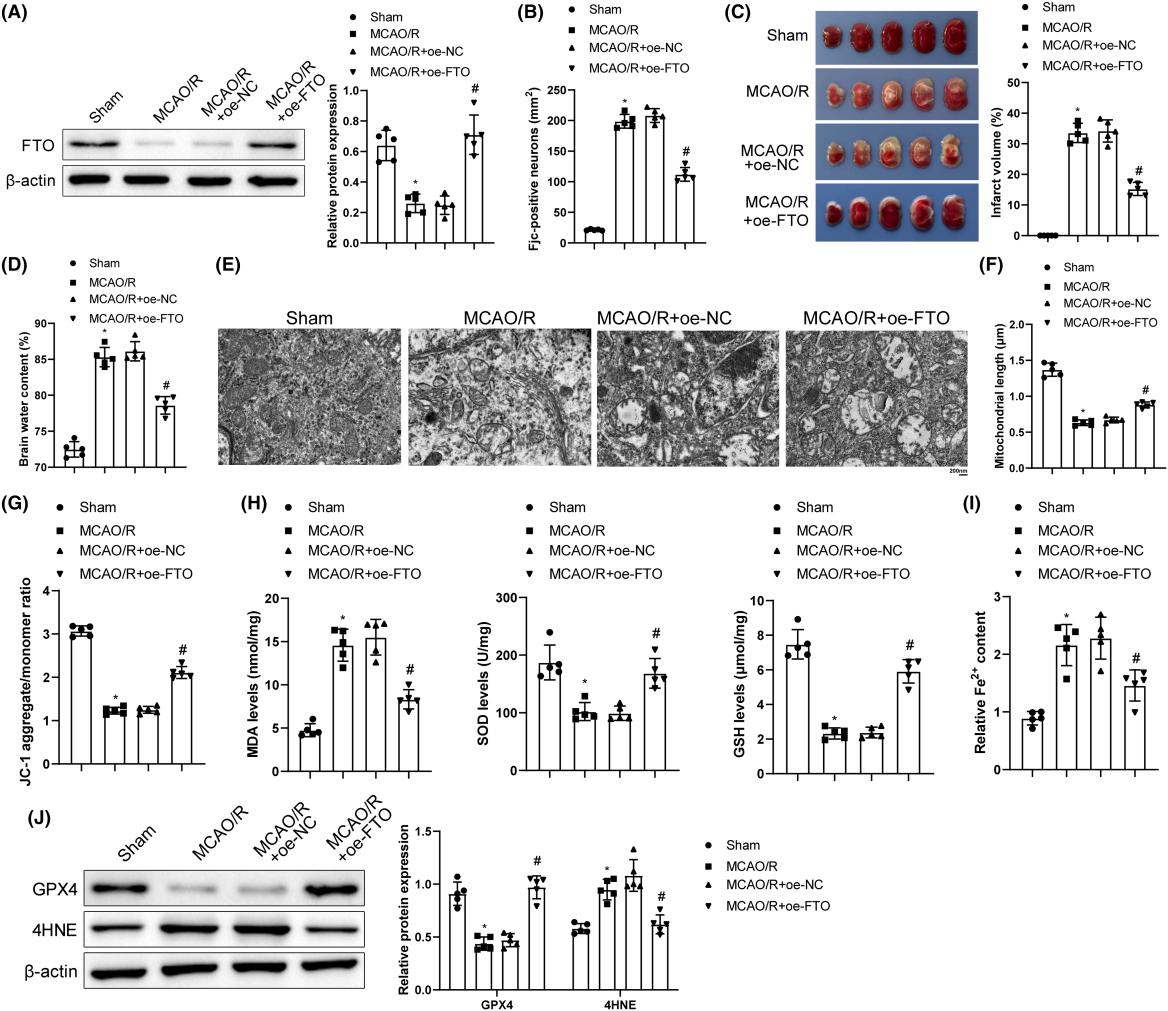
Overexpression of FTO inhibits mitochondrial fission to mitigate MCAO/R cerebral injury. (A) Western blot to detect the levels of FTO; (B) FJC staining to monitor the neuron degeneration; (C) TTC staining to examine the infarction area of brain; (D) Brain water content test to assess brain edema; (E) TEM to monitor mitochondrial ultrastructure; (F) Analysis of mitochondrial length; (G) JC‐1 kits to measure mitochondrial membrane potential; (H) ELISA to examine MDA, SOD, and GSH levels; (I) Iron detection kits to check Fe^2+^ content in brain tissues; (J)Western blot to assess GPX4 and 4HNE expression levels. *p < 0.05 versus the sham group; ^#^ *p* < 0.05 versus the MCAO/R + oe‐NC group. These values were measurement data and presented as mean ± standard deviation. One‐way ANOVA or Kruskal‐Wallis test was adopted for data comparisons among multiple groups. *N* = 5.

To explore the impact of FTO on cerebral I/R, we established a cerebral I/R mouse model and overexpressed FTO, followed by related detections. In comparison with the sham group, FTO expression was markedly decreased in the MCAO/R group, which was prominently elevated in the MCAO/R + oe‐FTO group when compared with the MCAO/R + oe‐NC group (Figure 1A). We provided complete uncropped blots of all western blot experiments (Supplemental Files). FJC staining was designed for the assessment of neuronal degeneration and showed that the MCAO/R group had a significant increase in FJC‐positive cells (vs. the sham group), while the MCAO/R + oe‐FTO group had reduced FJC‐positive cells (vs. the MCAO/R + oe‐NC group) (Figure 1B). Results of TTC staining manifested increased infarction volume in the MCAO/R group (vs. the sham group) but decreased infarction volume in the MCAO/R + oe‐FTO group (vs. the MCAO/R + oe‐NC group) (Figure 1C). As reflected in Figure 1D, the MCAO/R group had higher brain water content than the sham group while the MCAO/R + oe‐FTO group had lower brain water content than the MCAO/R + oe‐NC group. TEM observations revealed normal mitochondrial structure and clear mitochondrial cristae in the sham group but injured and cracked mitochondrial cristae and shortened mitochondrial length in neurons surrounding ischemic areas in the MCAO/R group. However, cotreatment of MCAO/R and FTO overexpression mitigated mitochondrial cristae damage and rupture and increased mitochondrial length (Figure 1E,F). In addition, downgraded ΔΨm levels (presented green) were noted in the MCAO/R group (vs. the sham group), which indicated weakened mitochondrial functions. Compared with the MCAO/R + oe‐NC group, the MCAO/R + oe‐FTO group showed increased ΔΨm levels (presented in red), indicating a significant increase in mitochondrial membrane potential (Figure 1G). ELISA unveiled increased MDA level and decreased SOD and GSH levels in the MCAO/R group (vs. the sham group) while opposite trends of these indicators were noted in the MCAO/R + oe‐FTO group when compared with the MCAO/R + oe‐NC group (Figure 1H). The MCAO/R group had higher Fe2^+^ content and 4HNE expression and lower GPX4 than those in the sham group, while Fe2^+^ content and 4HNE expression were diminished and GPX4 expression was enhanced following MCAO/R treatment and FTO overexpression (Figure 1I,J). The abovementioned results clarified that cerebral I/R induced mitochondrial fission, increased oxidative stress and ferroptosis, and decreased FTO expression. FTO overexpression suppressed OS and ferroptosis by subduing mitochondrial fission to restrain cerebral I/R injury.

### 3.2 Overexpression of FTO suppressed mitochondrial fission and ferroptosis in OGD/R hippocampal neurons

**FIGURE 2:**
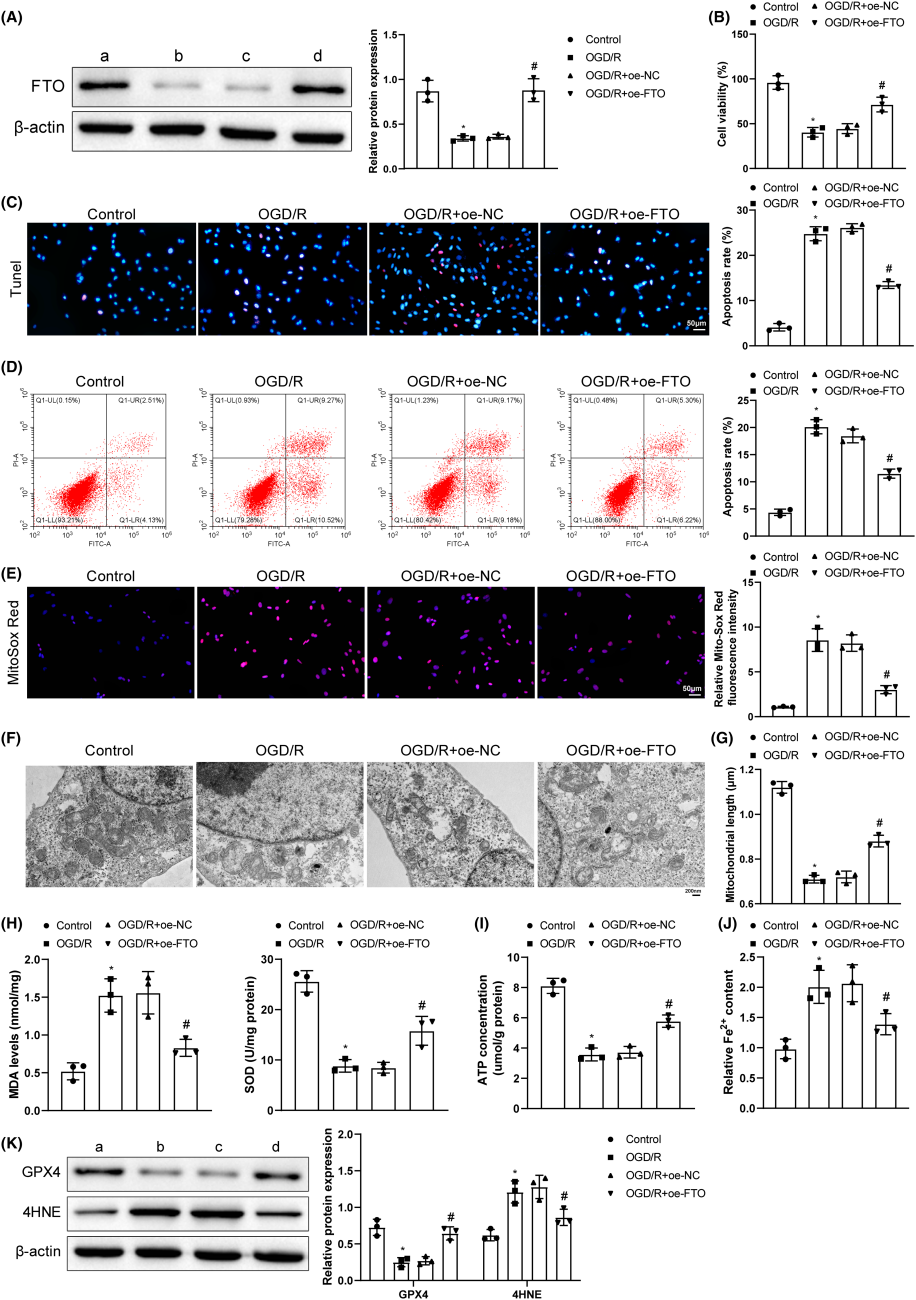
Overexpression of FTO reduces mitochondrial fission and ferroptosis and cell apoptosis in OGD/R cells. (A) Western blot measurement of FTO expression; (B) CCK‐8 detection of cell viability; (C) TUNEL observation of cell apoptosis; (D) Flow cytometry to detect cell apoptosis; (E) Mito‐Sox fluorescence probe determination of ROS generation; (F) TEM observation of mitochondrial conditions; (G) Mitochondrial length; (H) ELISA testing of MDA and SOD expression levels; (I) ATP detection kits of ATP levels; (J) Iron detection kits of Fe^2+^ content; (K) Western blot analysis of GPX4 and 4HNE expression. *p < 0.05 versus the control group, ^#^ *p* < 0.05 versus the OGD/R + oe‐NC group. These values were measurement data and presented as mean ± standard deviation. Kruskal–Wallis test was adopted for data comparisons among multiple groups. The experiment was repeated thrice.

For the exploration of the impact of FTO on OGD/R hippocampal neurons, the OGD/R cell models were induced. Western blot exhibited decreased FTO expression after OGD/R treatment and increased FTO expression after further treatment of FTO overexpression (Figure 2A). There were also decreased cell viability, increased cell apoptosis (Figure 2B–D), augmented ROS level, and worsened mitochondrial cristae injury and shortened mitochondrial length (Figure 2E–G) after OGD/R treatment, which was reversed by additional FTO overexpression. Furthermore, further overexpressing FTO offset the impacts of OGD/R treatment on cells. Specifically, SOD, ATP, and GPX4 levels were lifted and Fe^2+^ content and MDA and 4HNE levels declined (Figure 2H–K). Clearly, FTO overexpression curbed mitochondrial fission and ferroptosis, enhanced cell survival, and inhibited cell apoptosis.

### 3.3 Upregulation of FTO suppressed FYN expression by regulating FYN m6A modification

**FIGURE 3:**
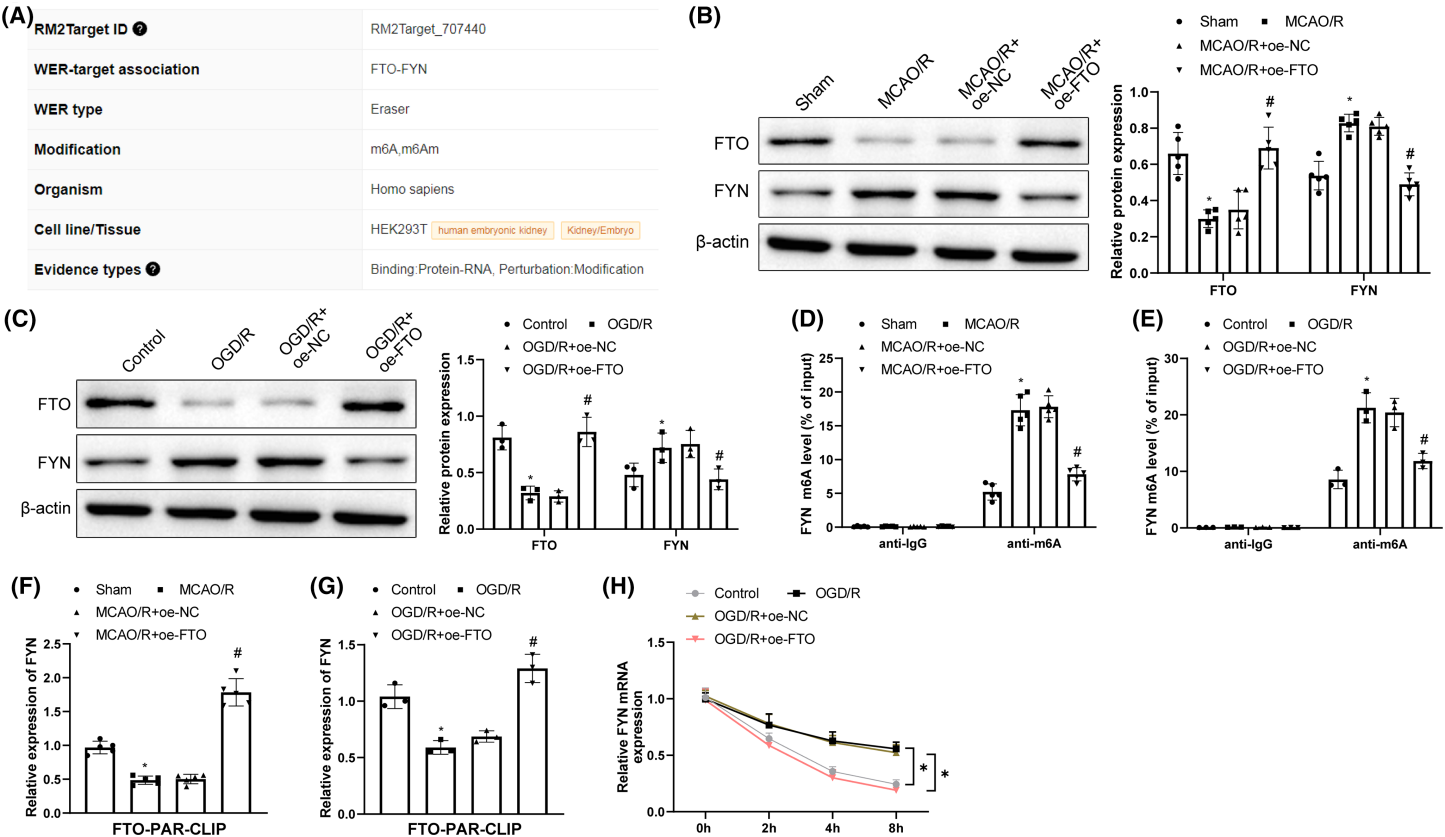
Overexpressing FTO suppresses FYN expression via the m6A modification of FYN. (A) RM2Target analysis of the binding relationship between FTO and FYN; (B, C) Western blot evaluation of FTO and FYN expression in cerebral I/R models; (D, E) Me‐RIP detection of FYN m6A modification levels; (F, G) PAR‐CLIP measurement of FTO binding to FYN mRNA; (H) Detection of stability of FYN mRNA using actinomycin D treatment. *p < 0.05 versus the sham or control group, ^#^ *p* < 0.05 versus the MCAO/R + oe‐NC or OGD/R + oe‐NC group. These values were measurement data and presented as mean ± standard deviation. One‐way ANOVA or Kruskal–Wallis test was adopted for data comparisons among multiple groups. *N* = 5 in animal experiments; cell experiments were conducted three times.

RM2Target bioinformatics analysis discovered that FTO might mediate the m6A modification of FYN (Figure 3A). To investigate the impact of FTO on the m6A modification of FYN, FTO and FYN expression were detected in mice with cerebral I/R. MCAO/R treatment decreased FTO expression and increased FYN expression while additional oe‐FTO treatment upregulated FTO and downregulated FYN (Figure 3B). In hippocampal neurons, compared with the control group, the OGD/R group showed a notable decrease in FTO expression and a notable increase in FYN expression; compared with the OGD/R + oe‐NC group, the OGD/R + oe‐FTO group had increased FTO and decreased FYN (Figure 3C). Me‐RIP uncovered that MCAO/R‐enhanced m6A modification level of *FYN* was reduced by further overexpressing FTO in mice (Figure 3D); in hippocampal neurons, compared with the control group, the OGD/R group showed m6A levels of *FYN*; compared with the OGD/R + oe‐NC group, the OGD/R + oe‐FTO group had decreased m6A levels of *FYN* (Figure 3E). PAR‐CLIP demonstrated a lowered volume of binding of FTO to *FYN* mRNA after MCAO/R, which was neutralized by further overexpression of FTO; similar results were also noted in hippocampal neurons (Figure 3F,G). The degradation rate of FYN mRNA notably reduced in the OGD/R group but accelerated by further overexpression of FTO (Figure 3H). It can be inferred that FYN was highly expressed in cerebral I/R and FTO overexpression inhibited the expression of *FYN* by reducing *FYN* mRNA m6A modification.

### 3.4 FYN overexpression aggravated MCAO/R cerebral injury in mice with overexpressed FTO

**FIGURE 4:**
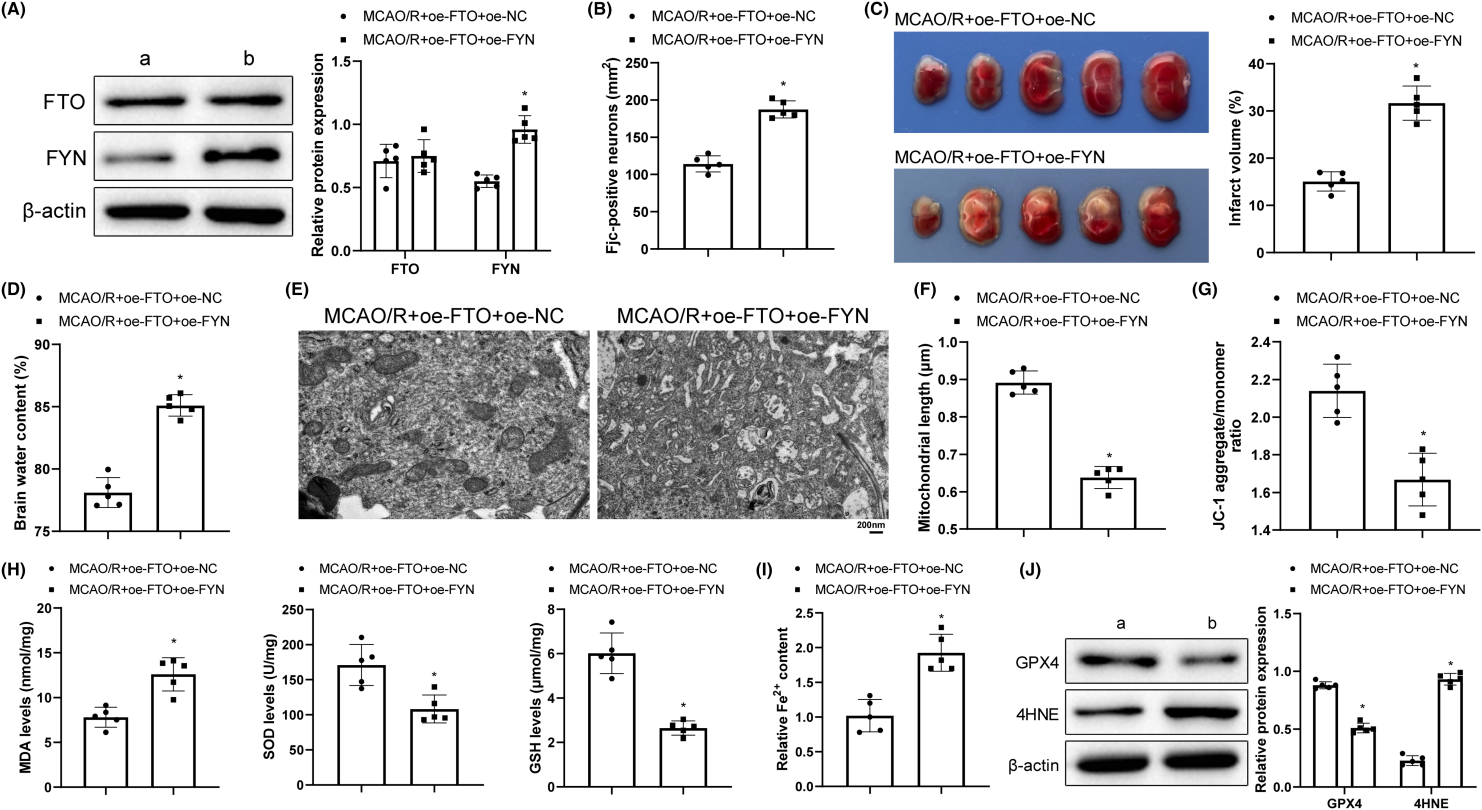
FYN overexpression exacerbates MCAO/R cerebral injury in mice with overexpressed FTO. **(**A) Western blot to measure FTO and FYN expression; (B) FJC staining to observe neuron degeneration; (C) TTC staining to monitor infraction size of brain; (D) Analysis of brain water content to evaluate brain edema; (E) TEM to monitor mitochondrial ultrastructure; (F) The length of mitochondria; (G) JC‐1 kits to assess mitochondrial membrane potentials; **(**H) ELISA to test MDA, SOD, and GSH levels; (I) Iron detection kits to assess Fe^2+^ content in brain tissues; (J) Western blot to determine GPX4 and 4HNE expression levels. *p < 0.05 versus the MCAO/R + oe‐FTO + oe‐NC group. These values were measurement data and presented as mean ± standard deviation. Unpaired t‐test or Mann–Whitney test was adopted for data comparisons between two groups. *N* = 5.

FTO and FYN were overexpressed simultaneously to search the impact of FTO/FYN on cerebral I/R injury, after which FYN was notably upregulated but FTO showed no significant difference compared with the single transfection of FTO overexpression (Figure 4A), together with increased FJC‐positive cells (Figure 4B), infarction size (Figure 4C), and brain water content (Figure 4D), worsened mitochondrial cristae damage and rupture, decreased mitochondrial length (Figure 4E,F), reduced levels of ΔΨm (Figure 4G), SOD, GSH, and GPX4, enhanced levels of MDA, and 4HNE, and augmented Fe^2+^ content (Figure 4H–J). Overall, co‐overexpression of FTO and FYN promoted mitochondrial fission and ferroptosis and aggravated cerebral I/R injury.

### 3.5 FTO upregulation restrained the activation of Drp1 pathway by inhibiting FYN expression and Drp1 phosphorylation

**FIGURE 5:**
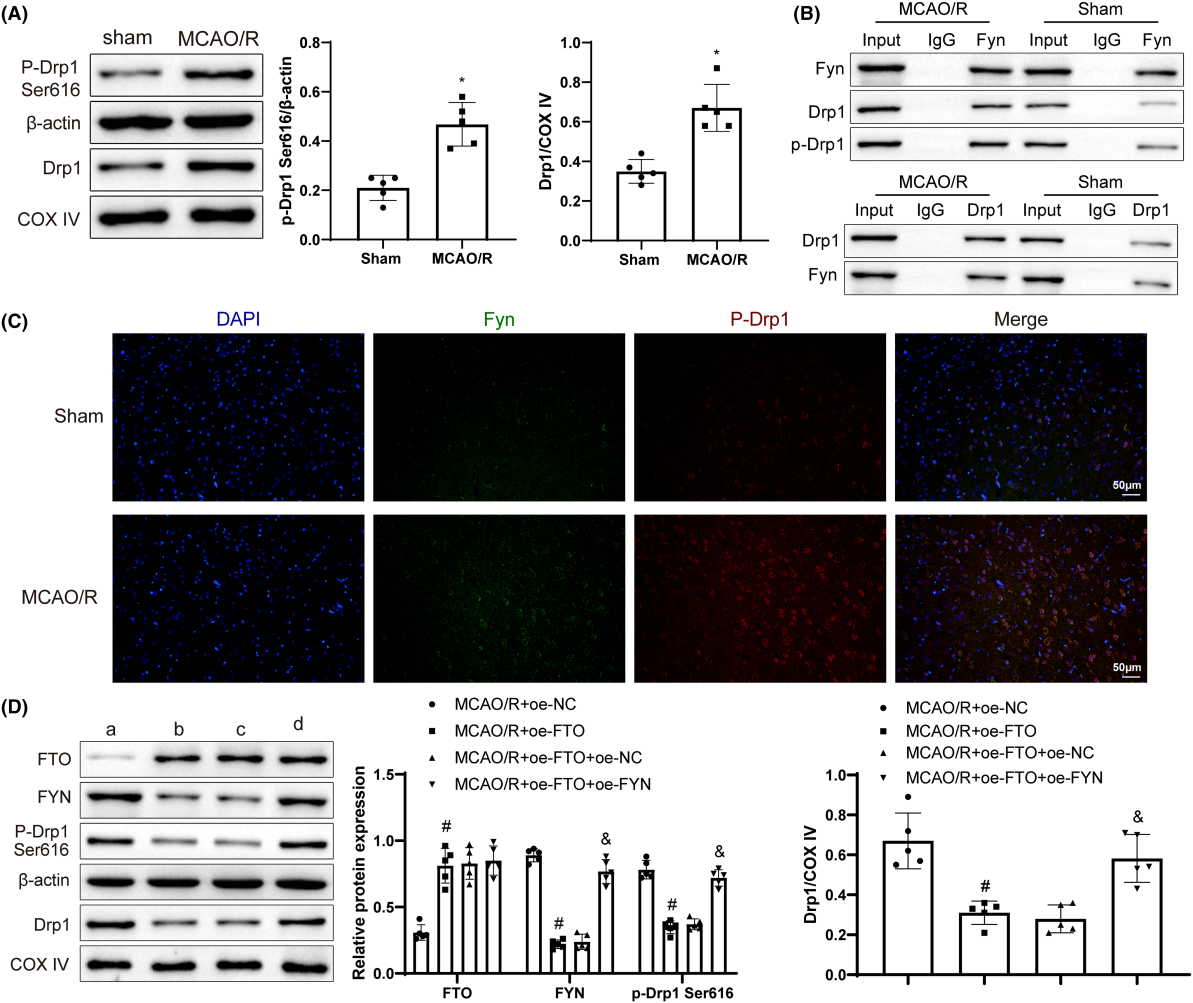
FTO upregulation represses FYN expression and Drp1 phosphorylation to inhibit the activation of Drp1 pathway. **(**A) Western blot to measure p‐Drp1 Ser616 and mitochondrial Drp1 levels; (B) Co‐IP to test the interaction between FYN and Drp1; (C) Immunofluorescence to detect FYN and p‐Drp1 Ser616 levels; (D) Western blot to measure FTO, FYN, p‐Drp1 Ser616, and mitochondrial Drp1 levels. *p < 0.05 versus the sham group, ^#^ *p* < 0.05 versus the MCAO/R + oe‐NC group, ^&^ *p* < 0.05 versus the MCAO/R + oe‐FTO + oe‐NC group. These values were measurement data and presented as mean ± standard deviation. Unpaired t‐test or Mann–Whitney test was adopted for data comparisons between two groups. One‐way ANOVA or Kruskal–Wallis test was adopted for data comparisons among multiple groups. *N* = 5.

For the objective of investigating Drp1 levels in cerebral I/R, p‐Drp1 Ser616/Drp1 and mitochondrial Drp1 level were tested. After MCAO/R treatment, p‐Drp1 Ser616/Drp1 level and mitochondrial Drp1 level were obviously increased (Figure 5A), the binding of FYN to p‐Drp1 enhanced (Figure 5B), and FYN and p‐Drp1 Ser616 levels augmented (Figure 5C). Further overexpression of FYN caused marked increases in FYN, p‐Drp1 Ser616/Drp1, and mitochondrial Drp1 levels but no conspicuous difference in FTO expression (Figure 5D). Altogether, overexpressed FTO inhibited FYN expression and Drp1 phosphorylation to repress the activation of Drp1 pathway.

### 3.6 FTO overexpression depressed mitochondrial functions in OGD/R cells via the FYN/Drp1 axis

**FIGURE 6:**
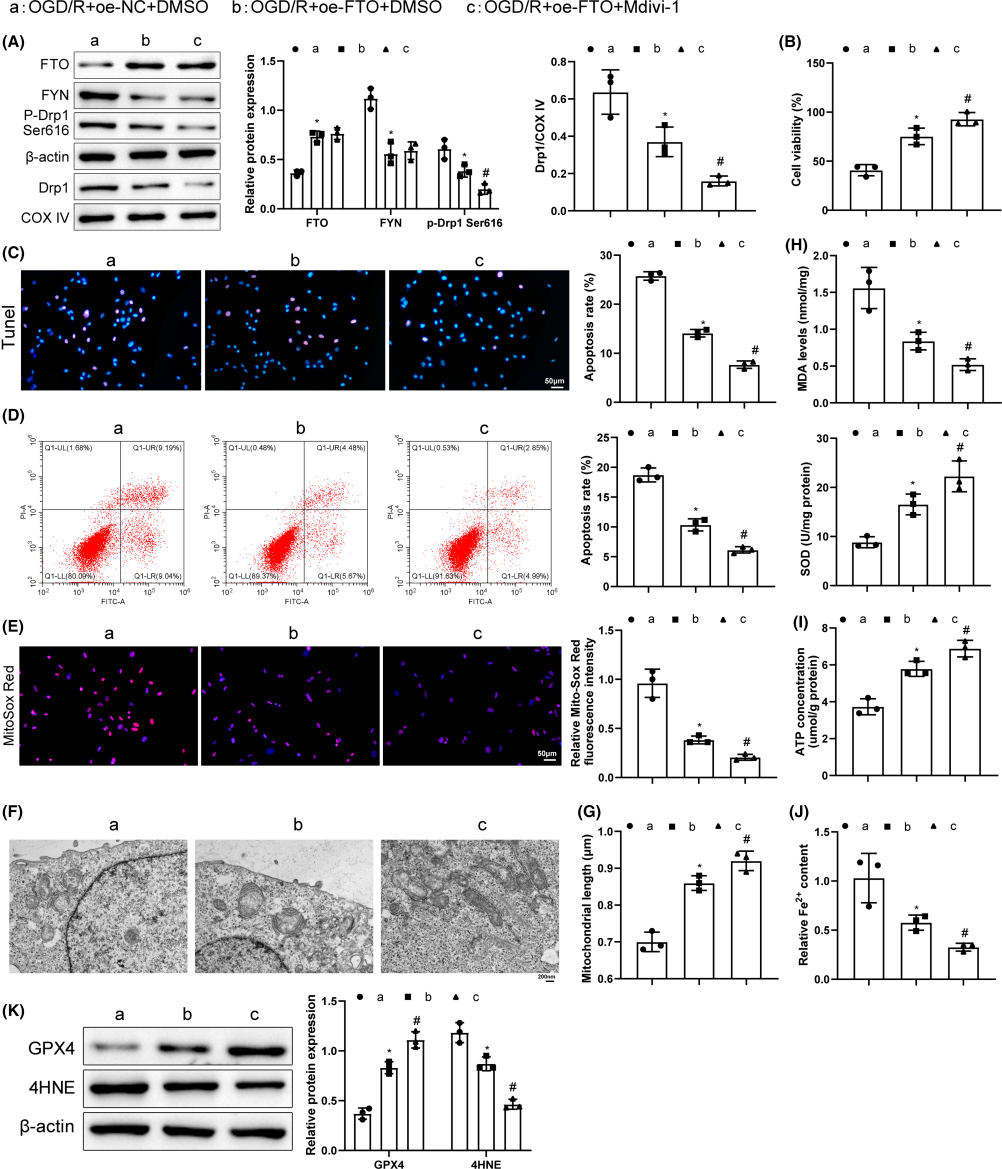
FTO overexpression relieves OGD/R cell injury by inhibiting mitochondrial functions via the FYN/Drp1 axis. (A) Western blot to measure FYN, p‐Drp1 Ser616, and mitochondrial Drp1 levels; (B) CCK‐8 to examine the cell viability; (C) TUNEL to detect the cell apoptosis; (D) Flow cytometry to detect cell apoptosis; (E) Mito‐Sox staining to test mitochondrial ROS levels; (F) TEM to assess mitochondrial conditions; (G) Mitochondrial length detection; (H) ELISA to detect MDA and SOD expression; (I) ATP detection kits to check ATP levels; (J) Iron content kits to examine Fe^2+^ content; (K) Western blot to measure GPX4 and 4HNE expression. *p < 0.05 versus the OGD/R + oe‐NC + DMSO group, ^#^ *p* < 0.05 versus the OGD/R + oe‐FTO + DMSO group. Kruskal–Wallis test was adopted for data comparisons among multiple groups. The experiment was repeated thrice.

To survey the effects of FTO/FYN/Drp1 on OGD/R cells, FTO overexpression and treatment of Drp1 inhibitor Mdivi‐1 were carried out in OGD/R cells, followed by related detections. FTO overexpression notably upregulated FTO and downregulated FYN, p‐Drp1 Ser616, and mitochondrial Drp1. Mdivi‐1 treatment further downregulated p‐Drp1 Ser616 and mitochondrial Drp1 levels (Figure 6A). Meanwhile, subsequent to FTO overexpression, cell viability elevated, cell apoptosis, ROS and mitochondrial cristae damage reduced, mitochondrial length increased, along with upgraded levels of SOD, ATP, and GPX4 and downgraded MDA and 4HNE levels and Fe^2+^ content. These trends were further augmented after the additional inhibition of Drp1 (Figure 6B–K). Collectively, FTO blocked the functions of mitochondria in OGD/R cells via the FYN/Drp1 axis.

### 3.7 FTO restrained mitochondrial fission via the FYN/Drp1 axis to inhibit MCAO/R cerebral injury

**FIGURE 7:**
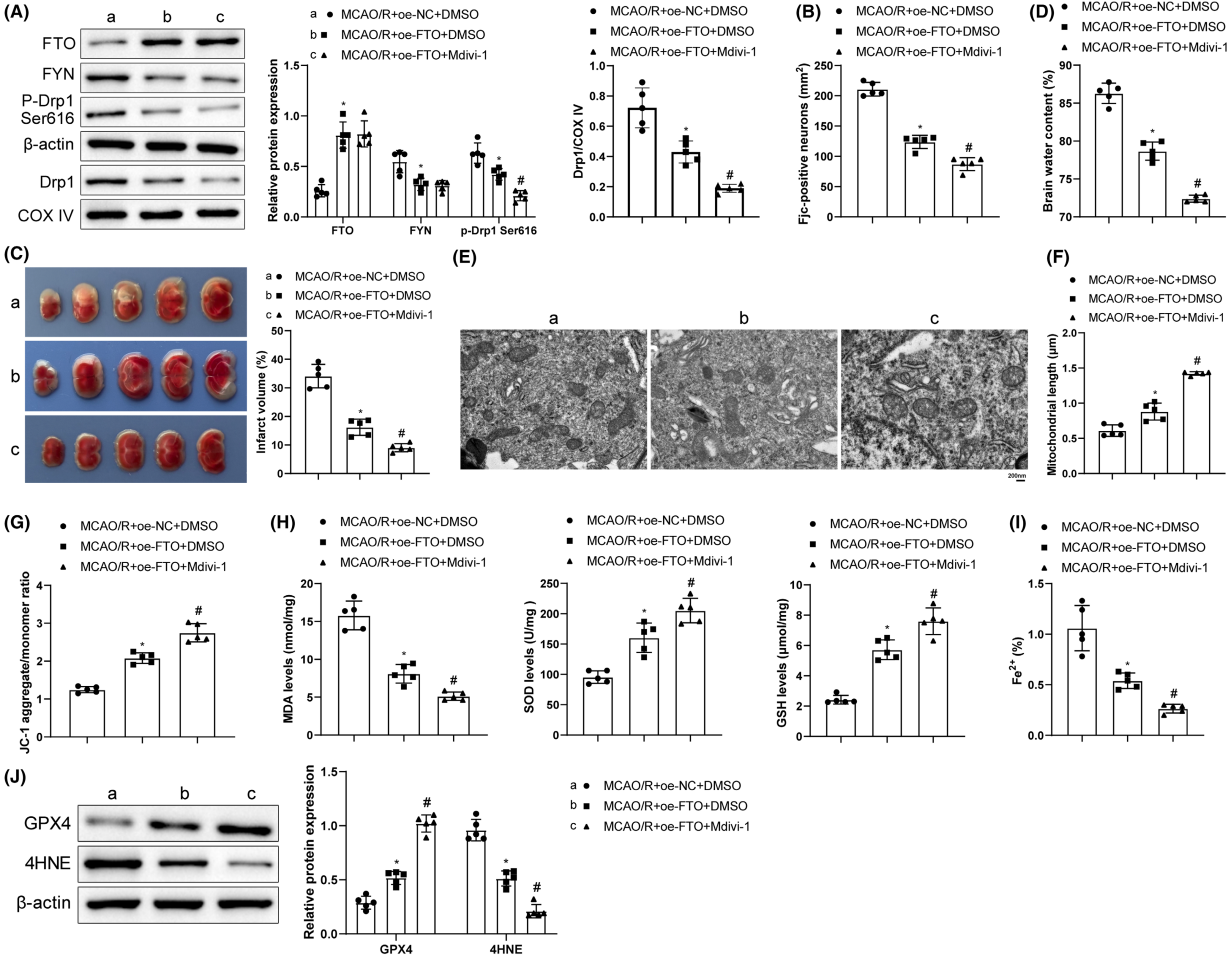
FTO suppresses mitochondrial fission to alleviate MCAO/R cerebral injury via the FYN/Drp1 axis. (A) Western blot analysis of FTO, FYN, p‐Drp1 Ser616, and mitochondrial Drp1 levels; (B) FJC staining observations of neuron degeneration; (C) TTC staining monitoring of infarction area; (D) Brain water content assessment of brain edema; (E) TEM observation of mitochondrial ultrastructure; (F) Measurement of mitochondrial length; (G) JC‐1 kit measurement of mitochondrial membrane potentials; (H) ELISA determination of MDA, SOD, and GSH levels; (I) Iron measurement kit examination of Fe^2+^ content in brain tissues; (J) Western blot analysis of GPX4 and 4HNE levels. *p < 0.05 versus the MCAO/R + oe‐NC + DMSO group, ^#^ *p* < 0.05 versus the MCAO/R + oe‐FTO + DMSO group. These values were measurement data and presented as mean ± standard deviation. One‐way ANOVA or Kruskal–Wallis test was adopted for data comparisons among multiple groups. *N* = 5.

We conducted FTO overexpression and Drp1 inhibition to dissect out the effects of FTO/FYN/Drp1 on cerebral I/R. Detection results unveiled that FTO overexpression greatly lifted FTO level and declined FYN, p‐Drp1 Ser616, and mitochondrial Drp1 levels, and that further Mdivi‐1 treatment signally lowered p‐Drp1/Drp1 and mitochondrial Drp1 levels (Figure 7A). After the transfection of FTO overexpression, FJC‐positive cells, cerebral infarction area, and brain water content were decreased; mitochondrial cristae injury and rupture were mitigated and mitochondrial length and ΔΨm level were augmented (Figure 7B–G), which were further enhanced by additional inhibition of Drp1. Moreover, after FTO overexpression, MDA, Fe^2+^, and 4HNE contents were markedly reduced and SOD, GSH, and GPX4 levels were elevated. These effects of FTO upregulation were enhanced by further treatment of Mdivi‐1 (Figure 7H–J). Overall, FTO upregulation inactivated Drp1 by silencing FYN to subdue OS and ferroptosis, thereby relieving cerebral I/R injury.

## 4 DISCUSSION

Cerebral I/R is a phenomenon of complexity, which needs large amount of experimental explorations to elucidate underlying regulatory mechanisms.^28^ FTO emerges as a novel research target in lately study and bioinformatics analysis of cerebral I/R.^29^, ^30^ To supplement detailed evidence of this protein affecting cerebral I/R injury to enrich the related theoretical basis, we carried out a series of operations at animal and cell levels and concluded that FTO overexpression reduced m6A modification of FYN to suppress FYN expression, thus inactivating Drp1 and reducing cerebral I/R injury.

OS is related to the pathogenesis of cerebral I/R injury.^31^ Inhibition of OS is important for protecting progranulin against cerebral I/R injury.^32^ Ferroptosis is another crucial inducer of cerebral I/R injury^33^ and its suppression could relieve cerebral I/R injury.^34^ In addition, mitochondrial fission appears as a marker of neural mitochondrial dysfunction, resulting from cerebral I/R.^35^, ^36^ Based on these, we detected mitochondrial functions (ATP, ΔΨm), and OS‐ and ferroptosis‐related factors (ROS, MDA, GSH, SOD; Fe^2+^, GPX4, 4HNE) in the established tMCAO/R mouse models and OGD/R cell models. Both models were commonly used in the research of cerebral I/R injury and other ischemic cerebrovascular diseases.^37^, ^38^, ^39^, ^40^ Our observations exhibited increased neuronal degeneration and brain water content, mitochondrial fission, and aggravated OS and ferroptosis after tMCAO/R in mice and inhibited cell viability and promoted apoptosis, mitochondrial fission, OS, and ferroptosis after OGD/R in cells. These trends indicate the successful model establishment.

FTO was reported to be downregulated in cerebral I/R injury,^21^ consistent with our findings that FTO expression declined after tMCAO/R or OGD/R treatment. FTO downregulation facilitated OS and cell apoptosis in myocardial I/R injury.^41^ We found that overexpression of FTO decreased MDA levels and increased SOD and GSH levels in cerebral I/R models. Moreover, FTO is a part of the regulation of ferroptosis in many diseases.^19^, ^42^ Our experiments unveiled reduced Fe^2+^ and 4HNE contents and enhanced GPX4 levels after overexpressing FTO in cerebral I/R models, along with reduced cerebral infarction and edema, and improved mitochondrial functions, as well as enhanced cell viability and weakened cell apoptosis in OGD/R neurons.

Earlier research discovered that FTO inhibits OS in cerebral I/R injury by mediating m6A modification of Nrf2 mRNA.^30^ As a demethylase, FTO is noted to suppress m6A modification of proteins to afflict their stability.^43^, ^44^ Our data proved that FYN was upregulated after tMCAO/R treatment and FTO overexpression reduced FYN expression by decreasing FYN mRNA m6A modification. Considering that FYN may regulate the process of cerebral I/R,^14^ we also designed related experiments to ascertain the specific effects of FYN on cerebral I/R injury. It was reported that the shortage of FYN contributed to the suppression of OS in diabetic renal fibrosis.^45^ Moreover, FYN nuclear expression was decreased when the OS‐induced injuries of endothelial cells were alleviated in ischemic stroke by oridonin.^46^ Our experimental recordings revealed that additional FYN overexpression reversed the anti‐OS effects of FTO overexpression on cerebral I/R injury. Furthermore, we also observed enhanced mitochondrial fission and ferroptosis in tMCAO/R‐treated mice after the co‐transfection of FTO overexpression and FYN overexpression. Likewise, previous research concluded that FYN plays a role in LPS‐induced acute kidney injury via the modulation of mitochondrial biogenesis and that its blockage ameliorated mitochondrial dysfunction in mice.^47^ FYN knockdown reduced brain edema, mitigated neuronal damage, and diminished apoptosis after intracerebral hemorrhage.^12^

Drp1 is a key mediator in mitochondrial dynamics and responsible for mitochondrial fission.^48^ Changes in Drp1 expression are important in OS injury, and cerebral I/R injury could elevate Drp1‐Ser616/Drp1 levels; after activation of Drp1 phosphorylation, it translocates to mitochondria to promote mitochondrial fission.^10^ FYN could motivate Drp1 signing via the phosphorylation of Drp1 at serine 616 to increase apoptosis in an intracerebral hemorrhage model.^12^ Therefore, we performed related assays to identify the role of the FYN/Drp1 axis and its association with FTO in the regulatory mechanism underlying cerebral I/R injury. Interestingly, the promotion of Drp1 phosphorylation was mentioned to aggravate OS‐induced neuron death.^49^ Moreover, the usage of Drp1 inhibitor alleviated the O‐GlcNAc transferase knockdown‐induced cerebral damage and infarction.^50^ Our study revealed elevated p‐Drp1 Ser616/Drp1 levels in MCAO/R models. Meanwhile, overexpressing FTO lessened FYN, p‐Drp1 Ser616/Drp1 and mitochondrial Drp1 levels, which was mitigated by further overexpression of FYN. Moreover, Drp1 inhibition enhanced the protective effects of FTO overexpression on mitochondrial fission, OS, ferroptosis, and cell viability on cerebral injury in animal and cell models. Drp1 was a downstream target of FTO; overexpression of FTO ameliorated hepatic I/R injury and repressed liver OS and mitochondrial fragmentation by inhibiting Drp1, and overexpression of Drp1 abrogated the protective effect of FTO.^51^ Drp1‐Ser616 phosphorylation and Drp1 translocation to the mitochondria can result in massive reactive oxygen species accumulation and cardiomyocyte necrosis.^11^ It was clear that FTO affects the FYN/Drp1 axis via the regulation of Drp1 phosphorylation to protect the brain against I/R injury.

**FIGURE 8:**
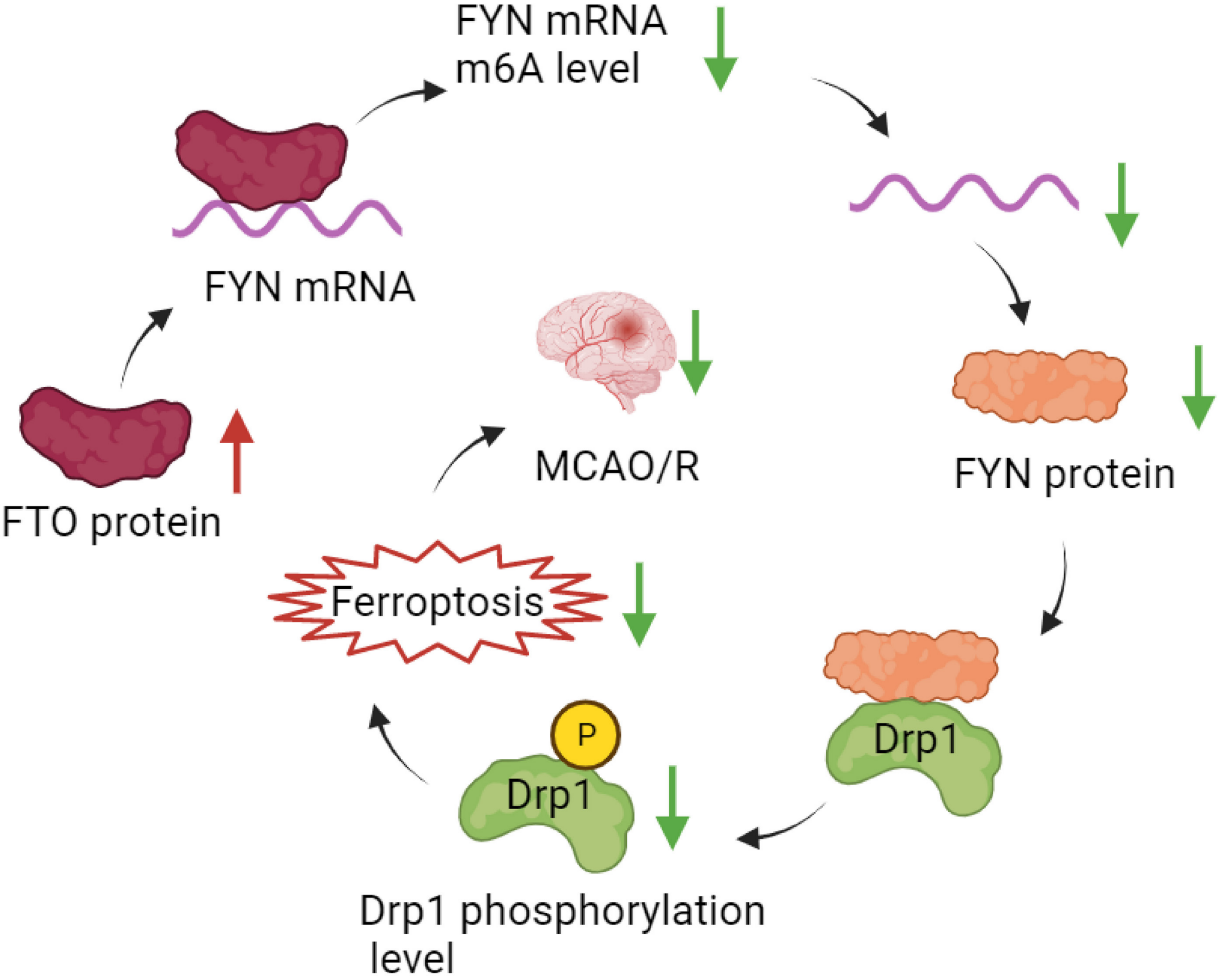
FTO downregulates the m6A level of FYN mRNA, inhibits FYN expression, downregulates FYN protein expression, inhibits phosphorylation of Drp1 protein bound to FYN, inhibits neuronal ferroptosis, and ultimately alleviates MCAO/R cerebral injury.

To sum up, we elucidated through animal and cell experiments that FTO overexpression inhibited FYN by reducing m6A modification of FYN to inactivate Drp1 and ameliorate mitochondrial fission, OS, and ferroptosis in cerebral I/R injury (Figure 8). Our research findings might help improve the current understanding of the molecular mechanisms underlying this condition and provide targets for I/R injury treatment. In addition, this study mainly studied the protective effect of FTO‐mediated FYN/Drp1 axis on neurons, and whether it will have similar effects on other cells of brain tissue (such as glial cells and endothelial cells) remains to be further verified. While encouraging results have been obtained in experimental studies, these benefits have not yet translated into clinical settings. Therefore, more studies are needed in the future to further characterize this molecular mechanism.

## AUTHOR CONTRIBUTIONS

ZY and GX conceived the ideas. ZY and GX designed the experiments. ZY performed the experiments. ZY and GX analyzed the data. ZY provided critical materials. ZY and GX wrote the manuscript. GX supervised the study. All the authors have read and approved the final version for publication.

## FUNDING INFORMATION

None.

## CONFLICT OF INTEREST STATEMENT

The authors declare there is no conflict of interests.

## CONSENT FOR PUBLICATION

All authors have consented to the submission of the manuscript.

## CONSENT TO PARTICIPATE

Not applicable.

## Supporting information


Figures S1–S7

## Data Availability

The datasets used or analyzed during the current study are available from the corresponding author on reasonable request.
