# Supplementary material for: Fat mass and obesity associated protein inhibits neuronal ferroptosis via the FYN/Drp1 axis and alleviate cerebral ischemia/reperfusion injury
Source: CNS Neurosci Ther. 2024 Mar 2;30(3):e14636. doi: 10.1111/cns.14636 (PMC10908355; doi:10.1111/cns.14636)

Full unedited gel/blot for Figure 1A-FTO

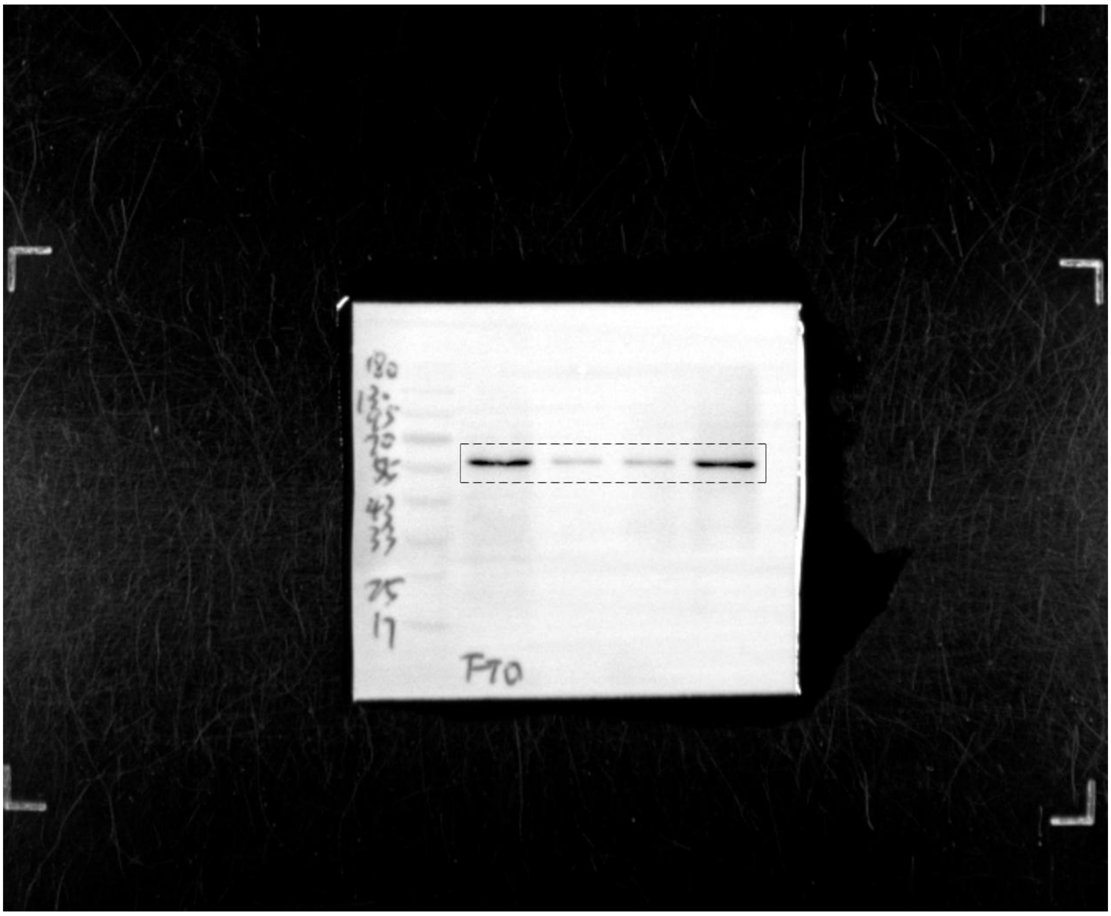

Full unedited gel/blot for Figure 1A-β-actin

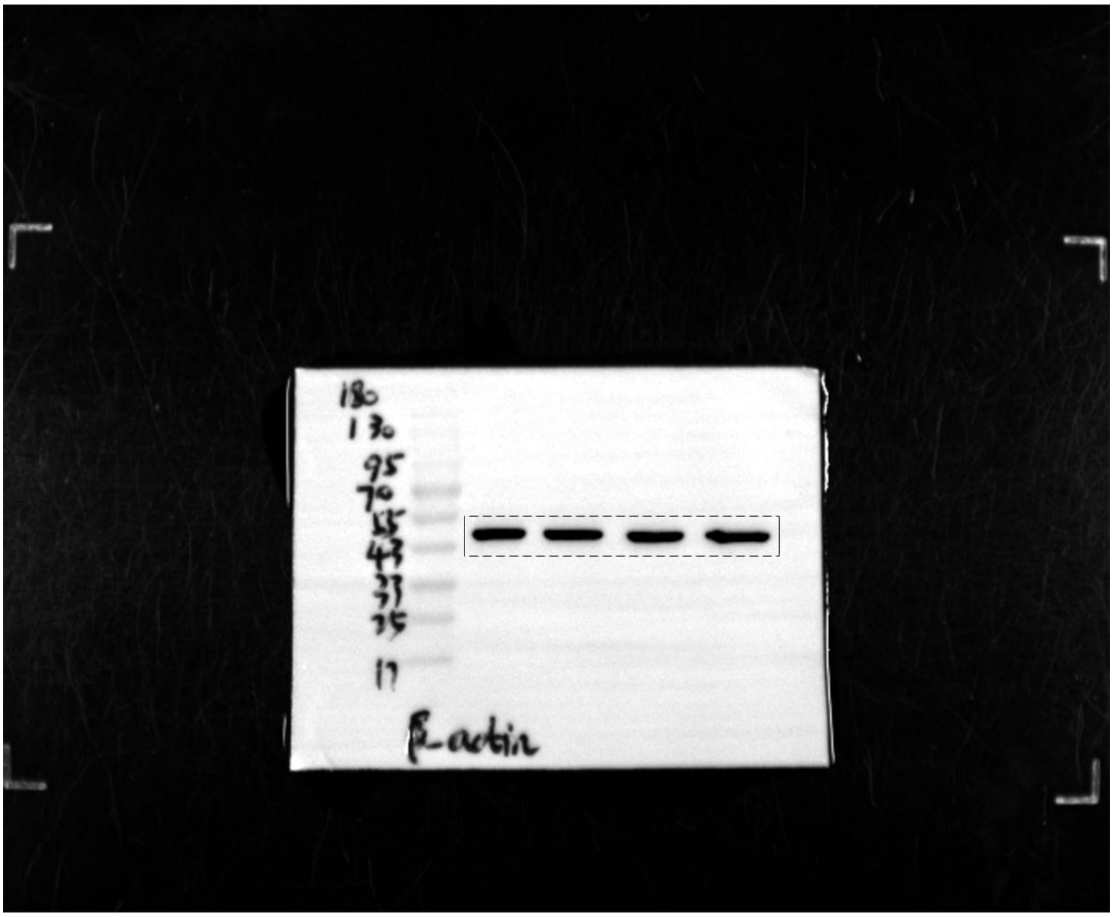

Full unedited gel/blot for Figure 1J-4-HNE

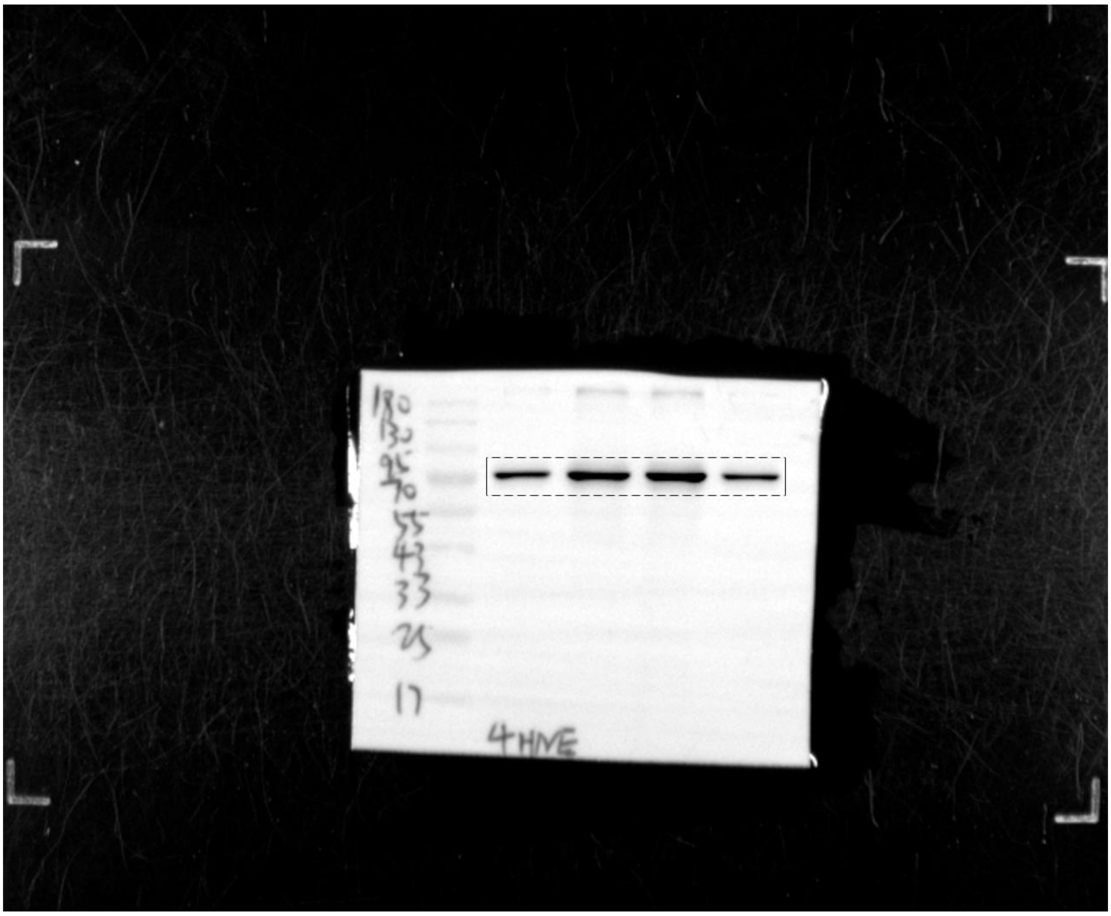

Full unedited gel/blot for Figure 1J-GPX4

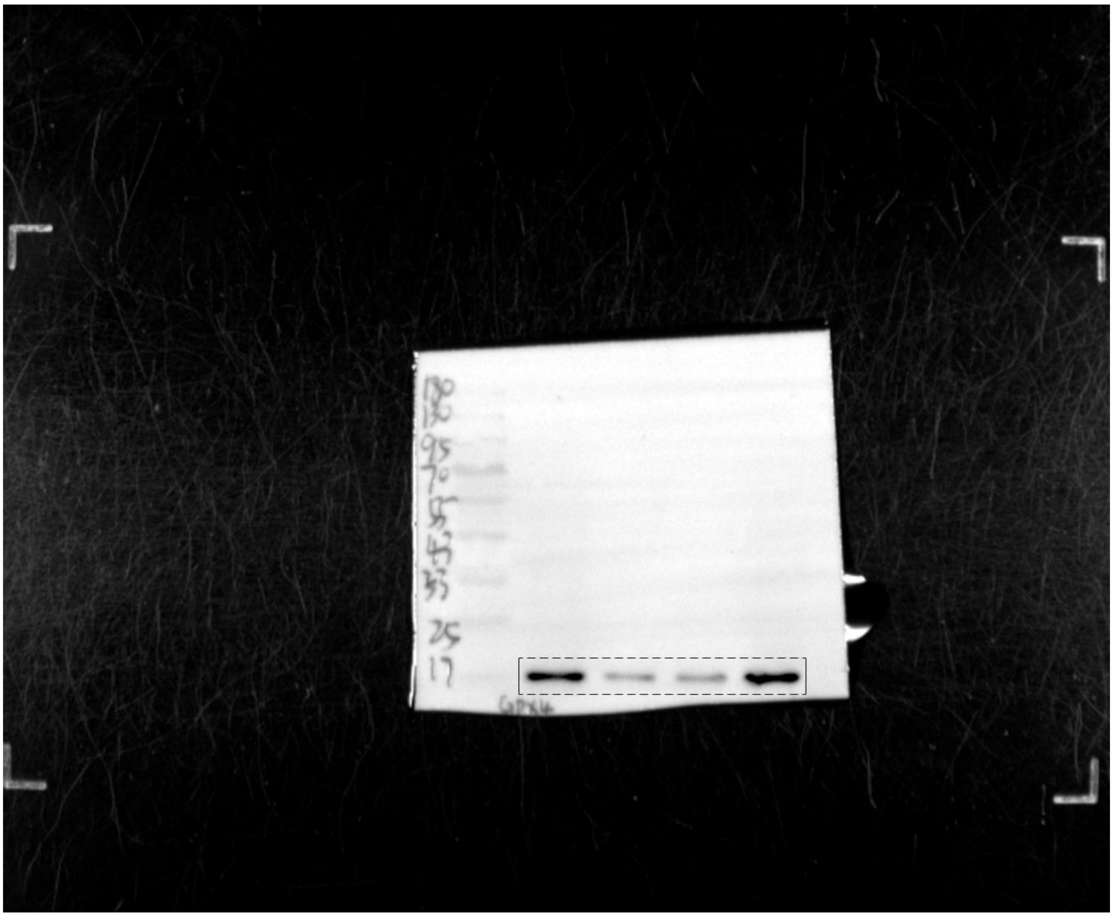

Full unedited gel/blot for Figure 1J-β-actin

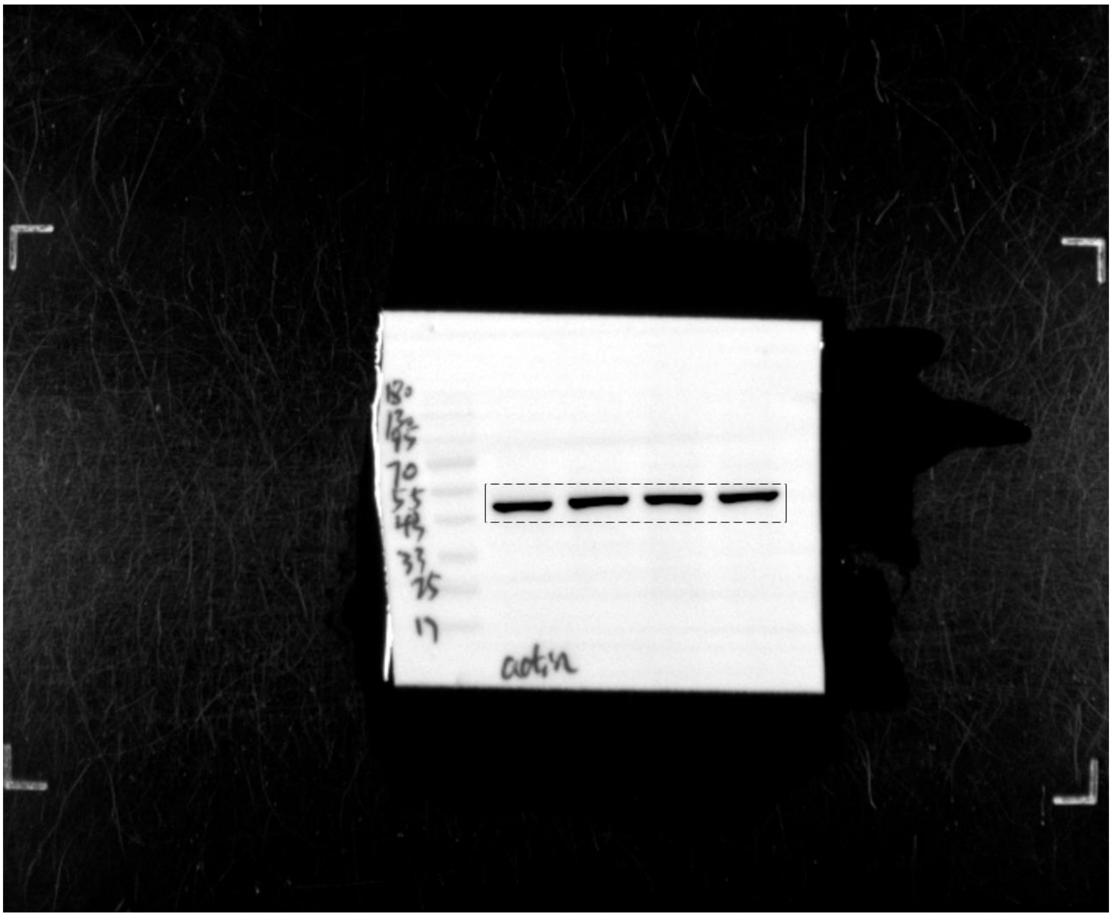

Full unedited gel/blot for Figure 2A-FTO

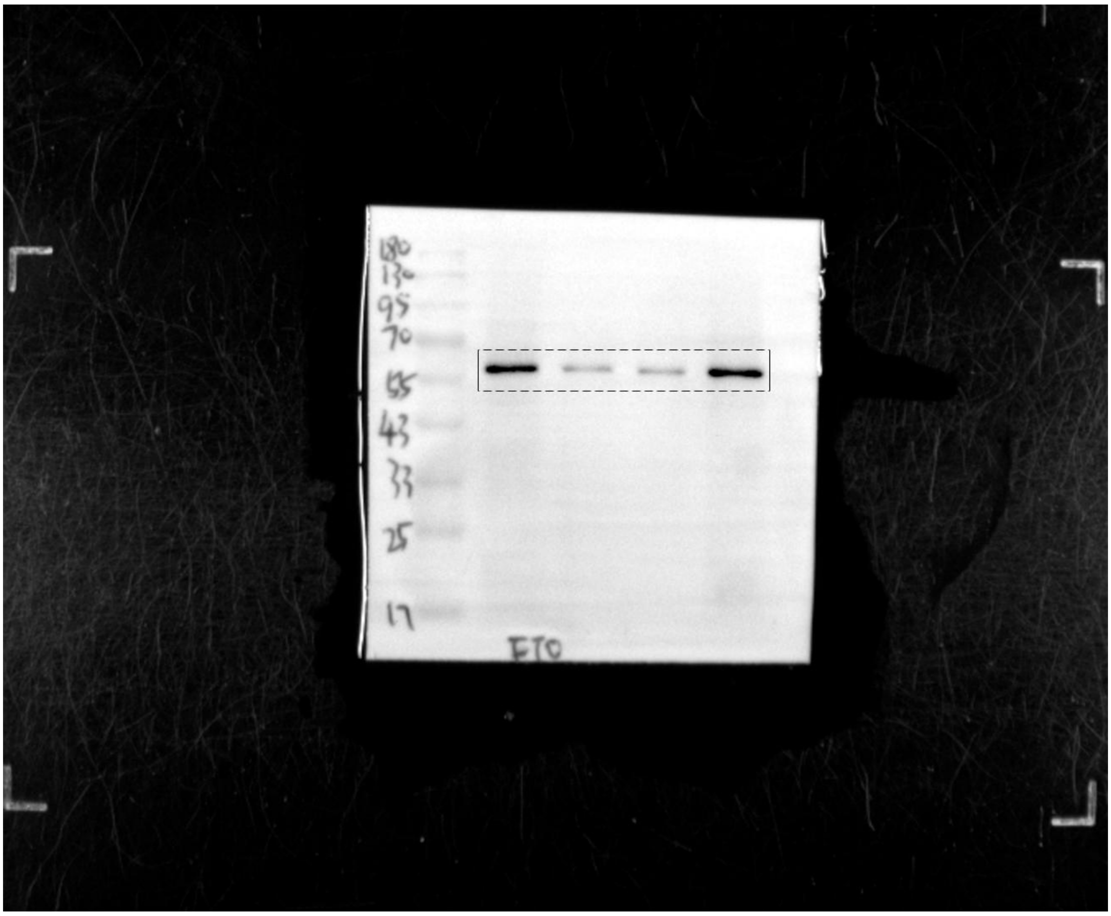

Full unedited gel/blot for Figure 2A-β-actin

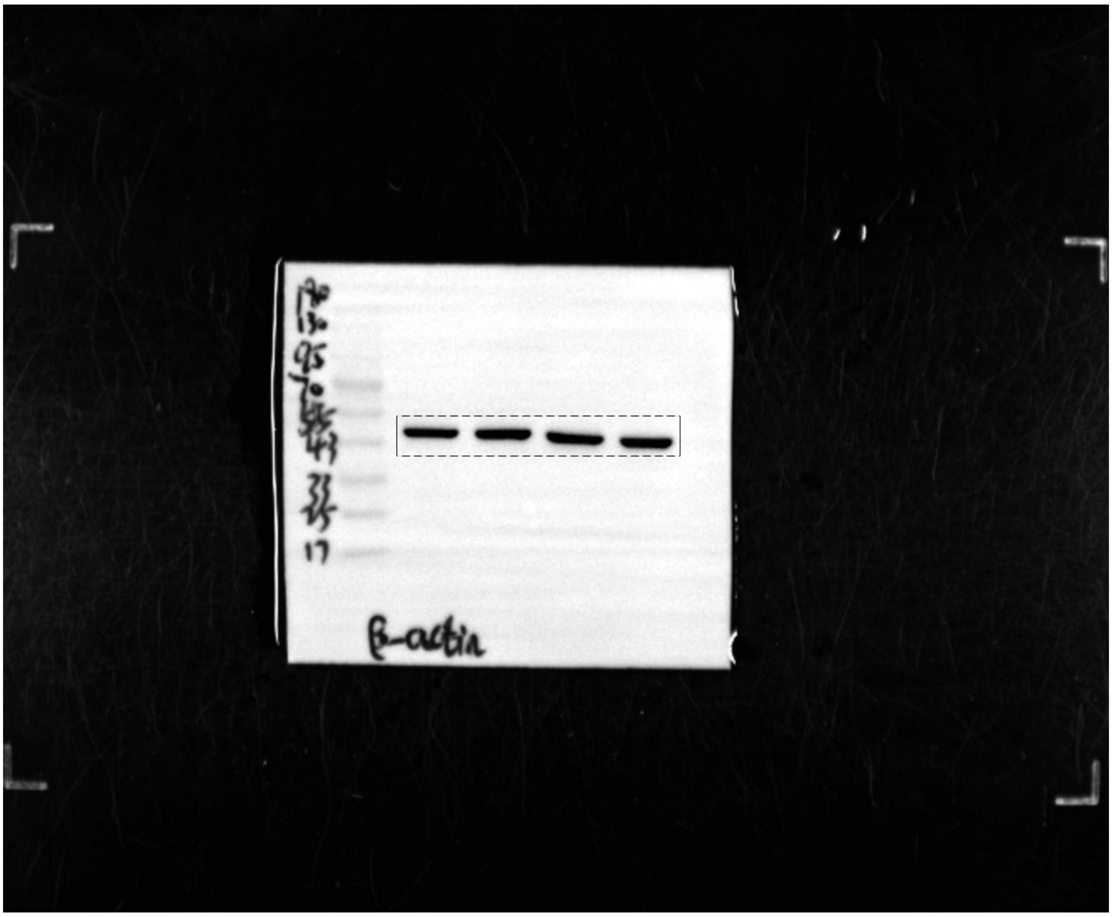

Full unedited gel/blot for Figure 2K-4-HNE

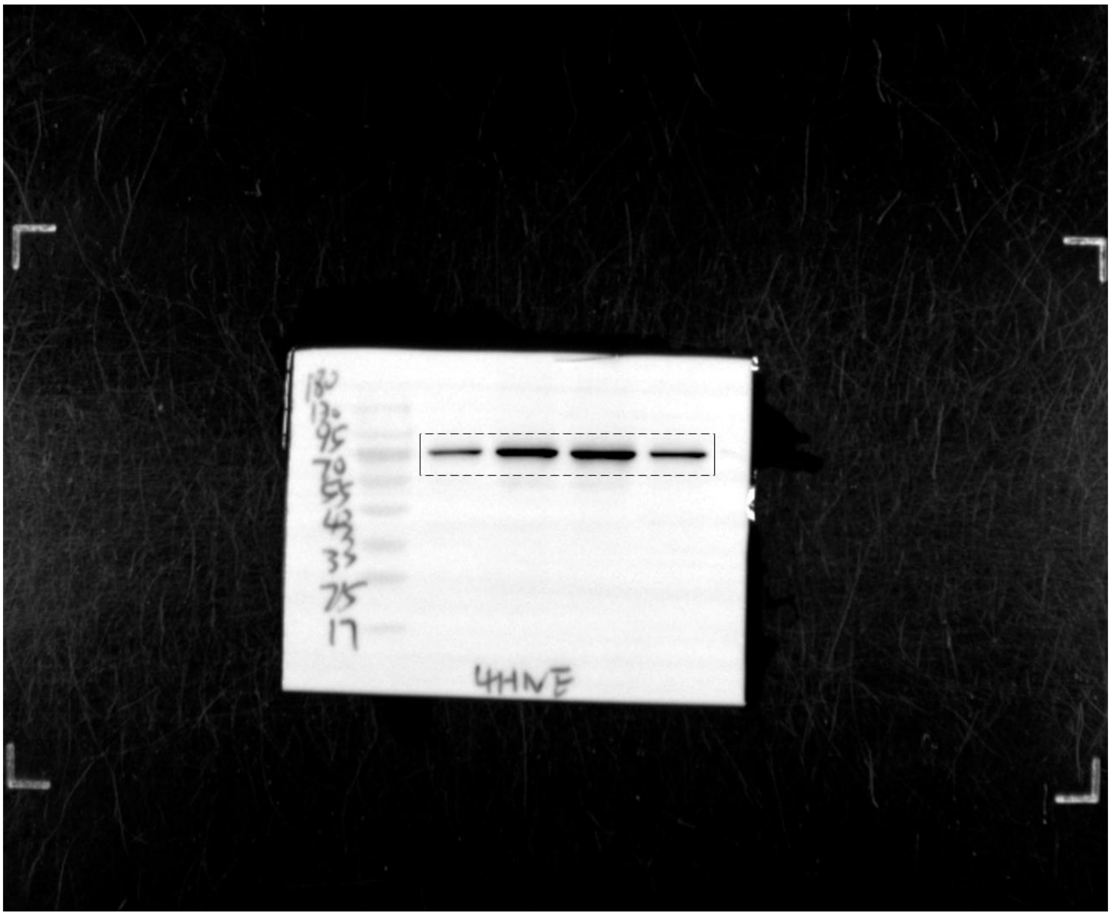

Full unedited gel/blot for Figure 2K-GPX4

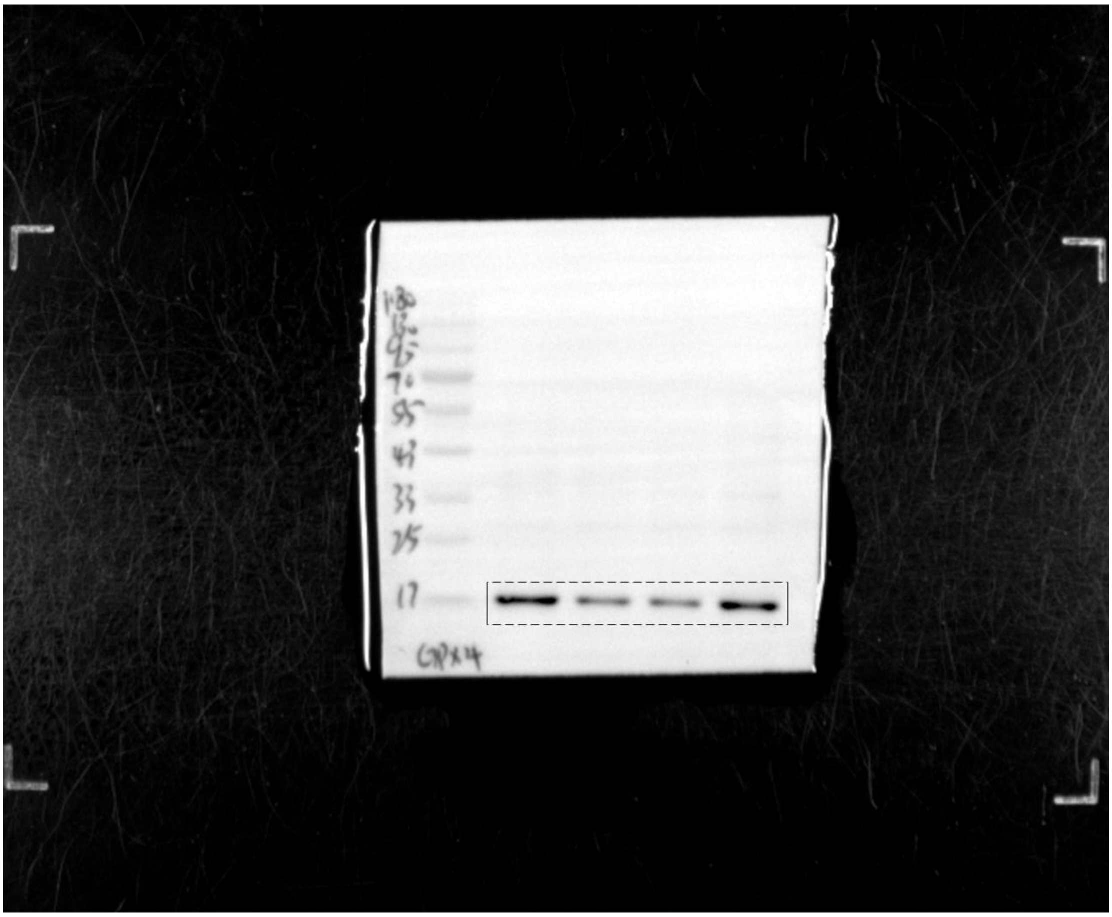

Full unedited gel/blot for Figure 2K-β-actin

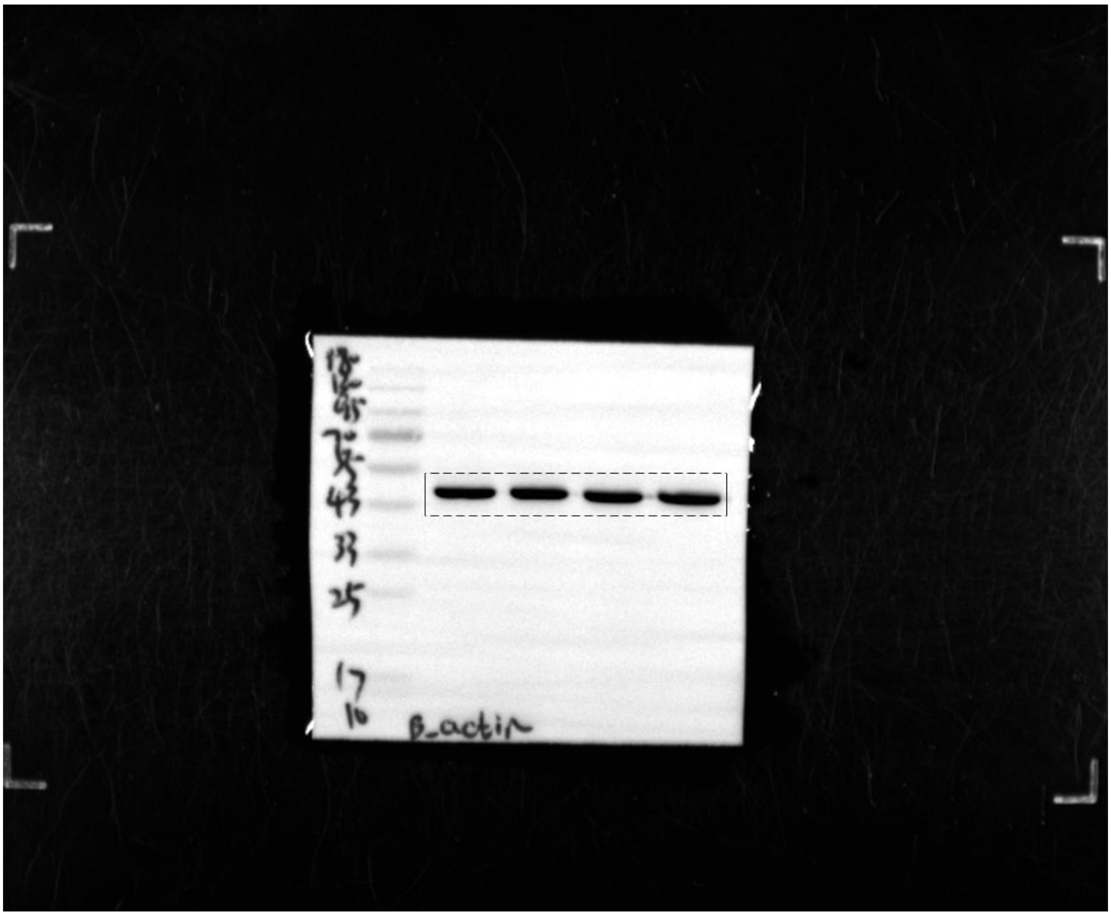

Full unedited gel/blot for Figure 3B-FTO

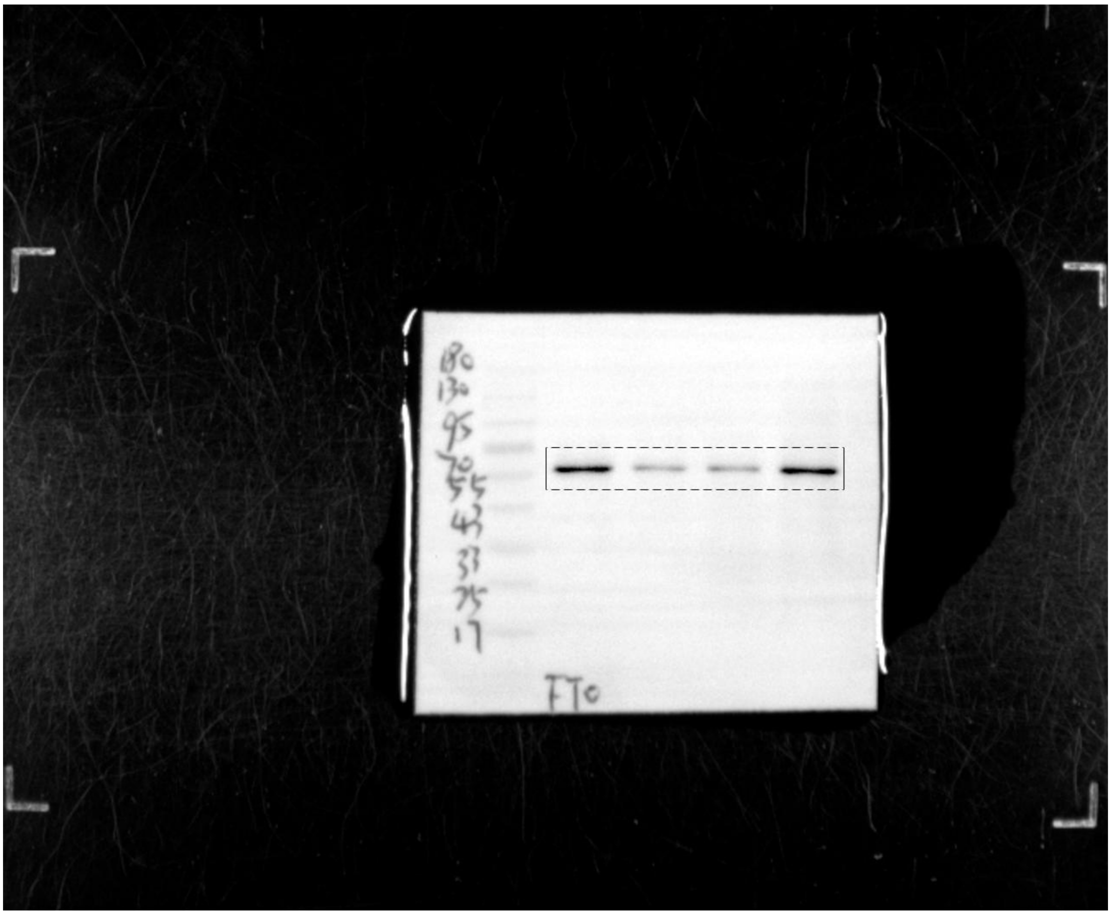

Full unedited gel/blot for Figure 3B-FYN

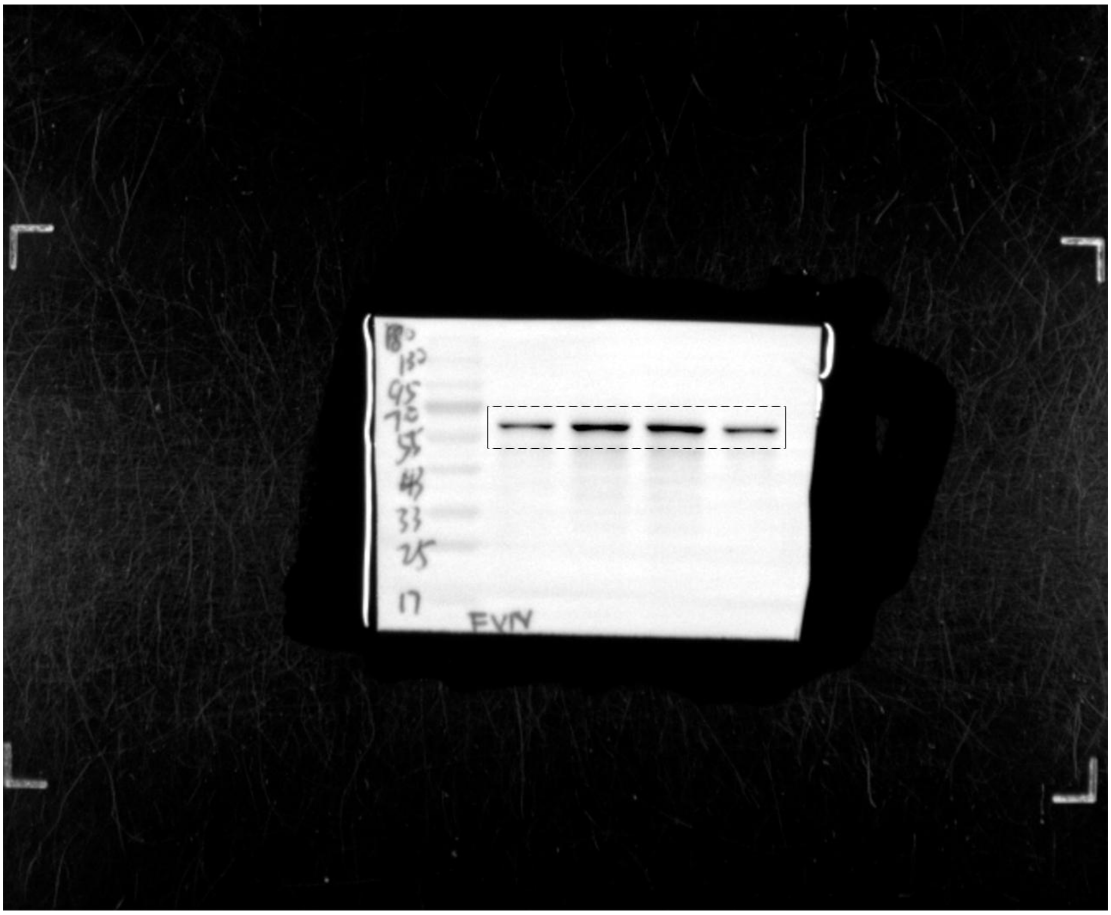

Full unedited gel/blot for Figure 3B-β-actin

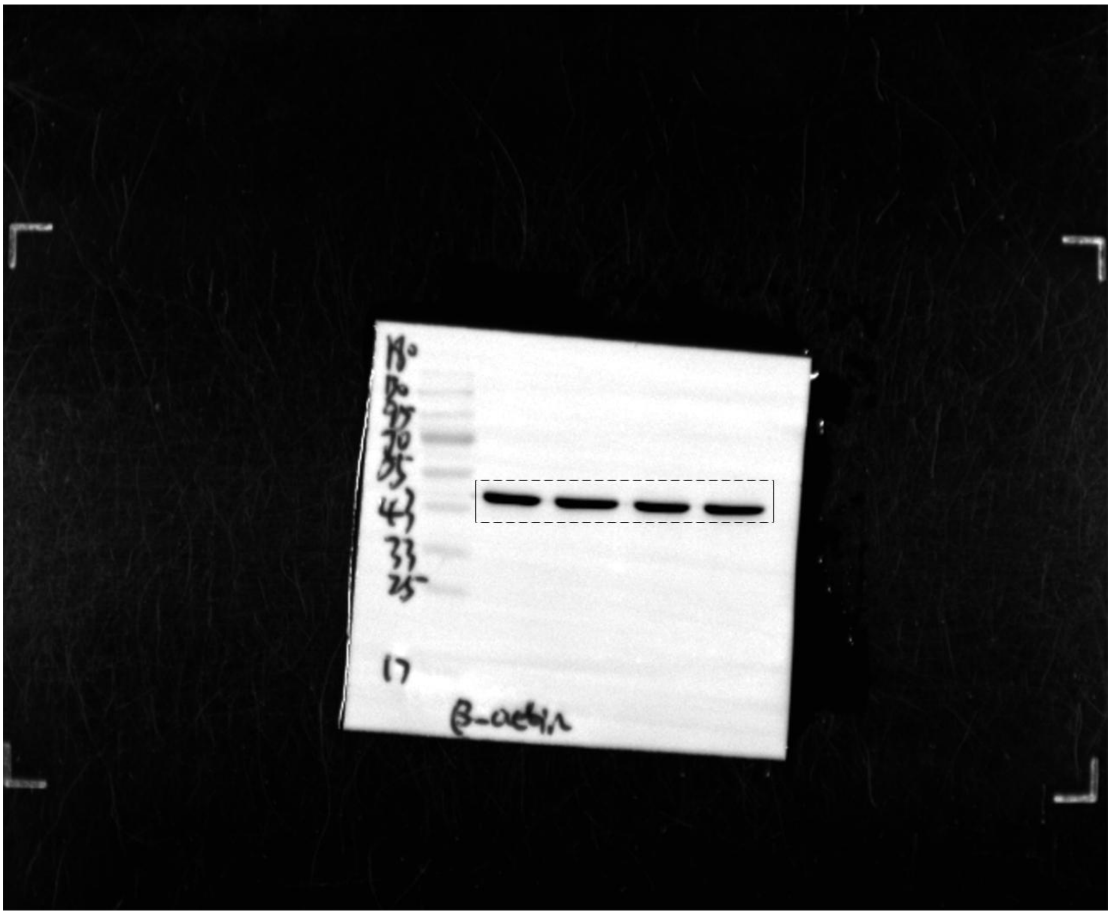

Full unedited gel/blot for Figure 3C-FTO

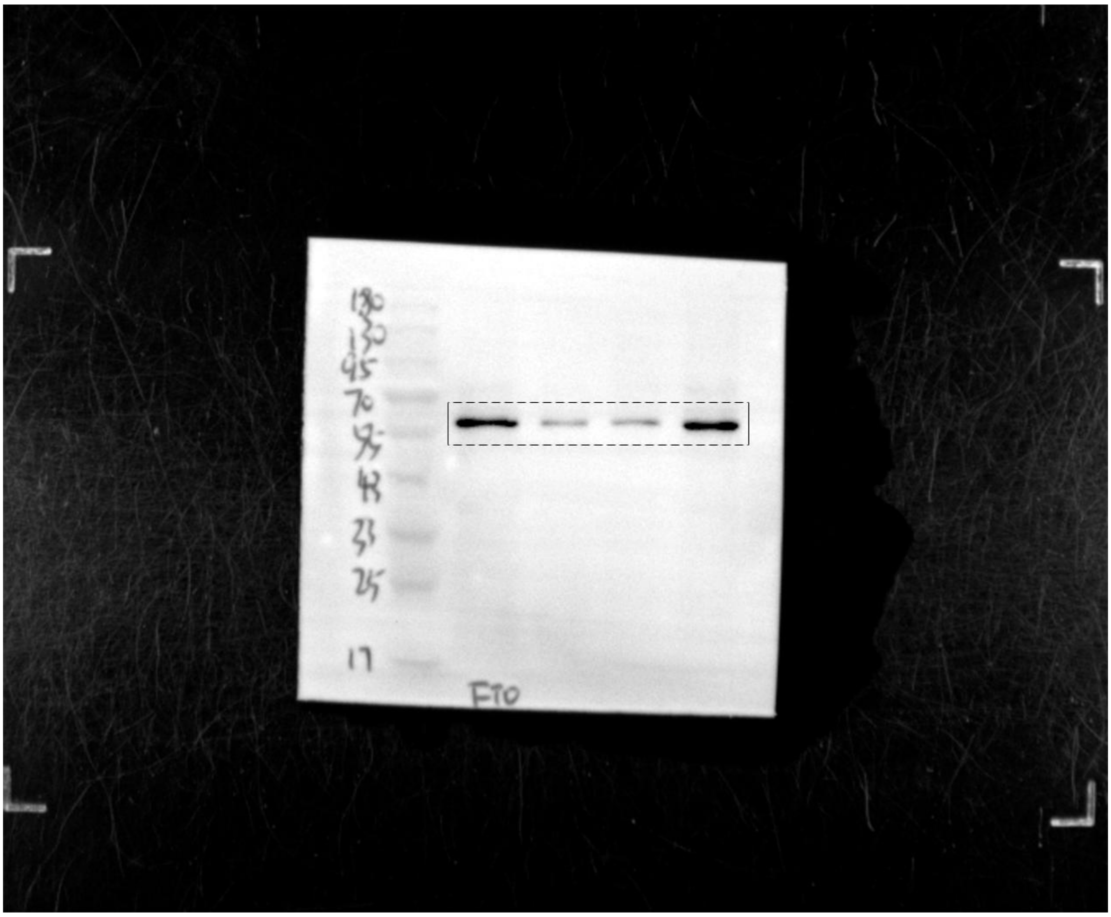

Full unedited gel/blot for Figure 3C-FYN

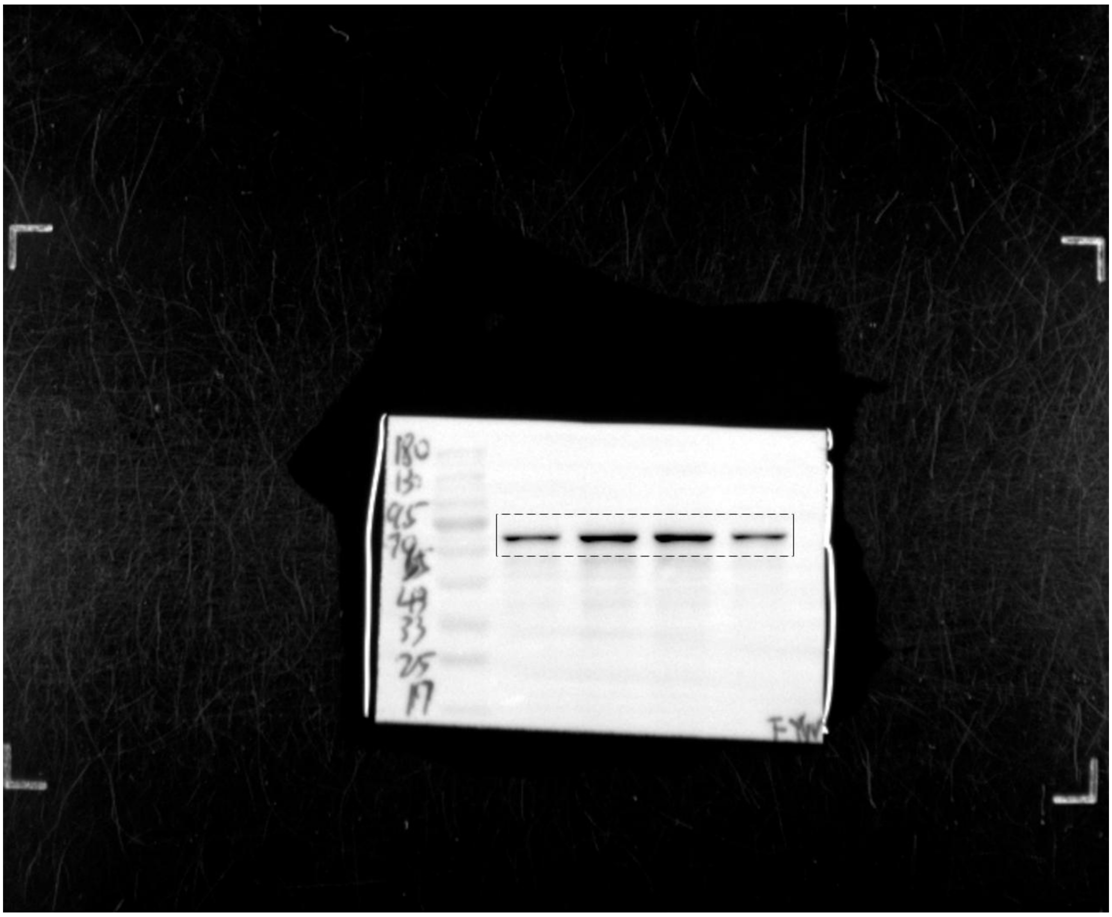

Full unedited gel/blot for Figure 3C-β-actin

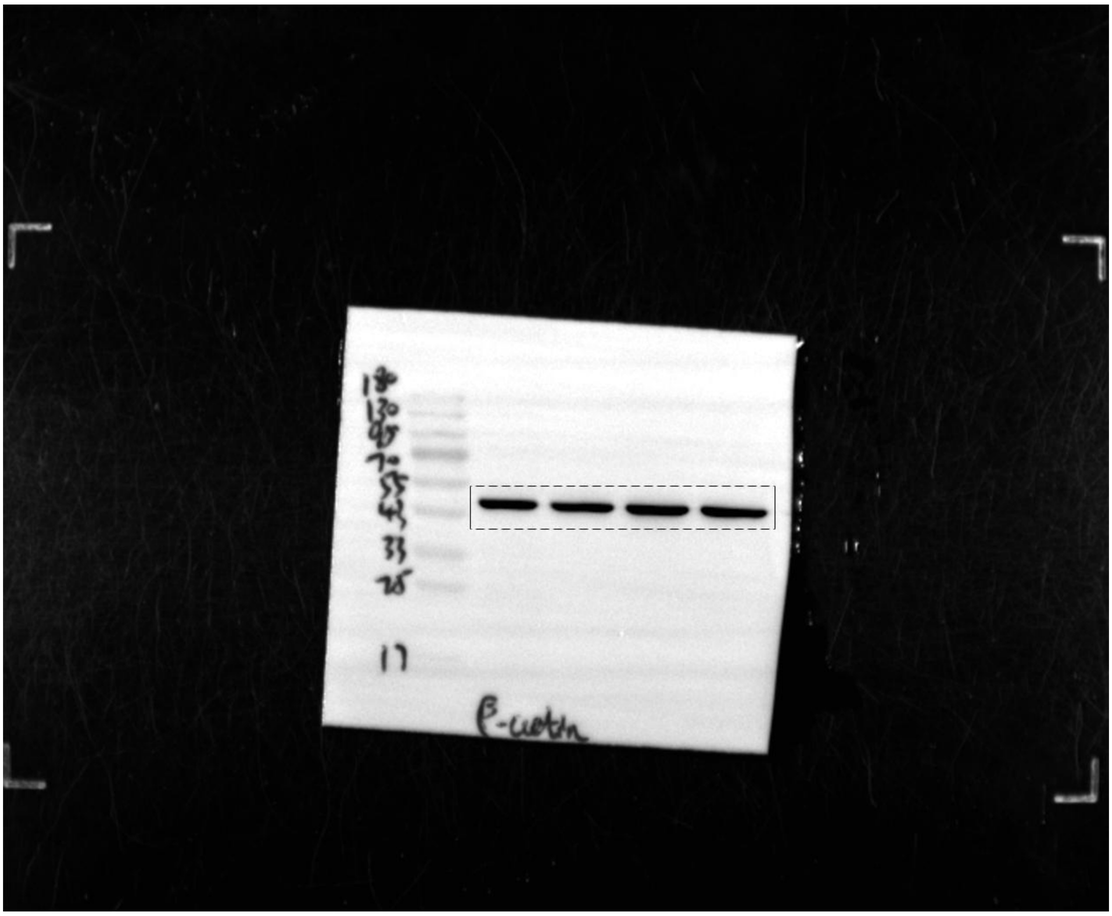

Full unedited gel/blot for Figure 4A-FTO

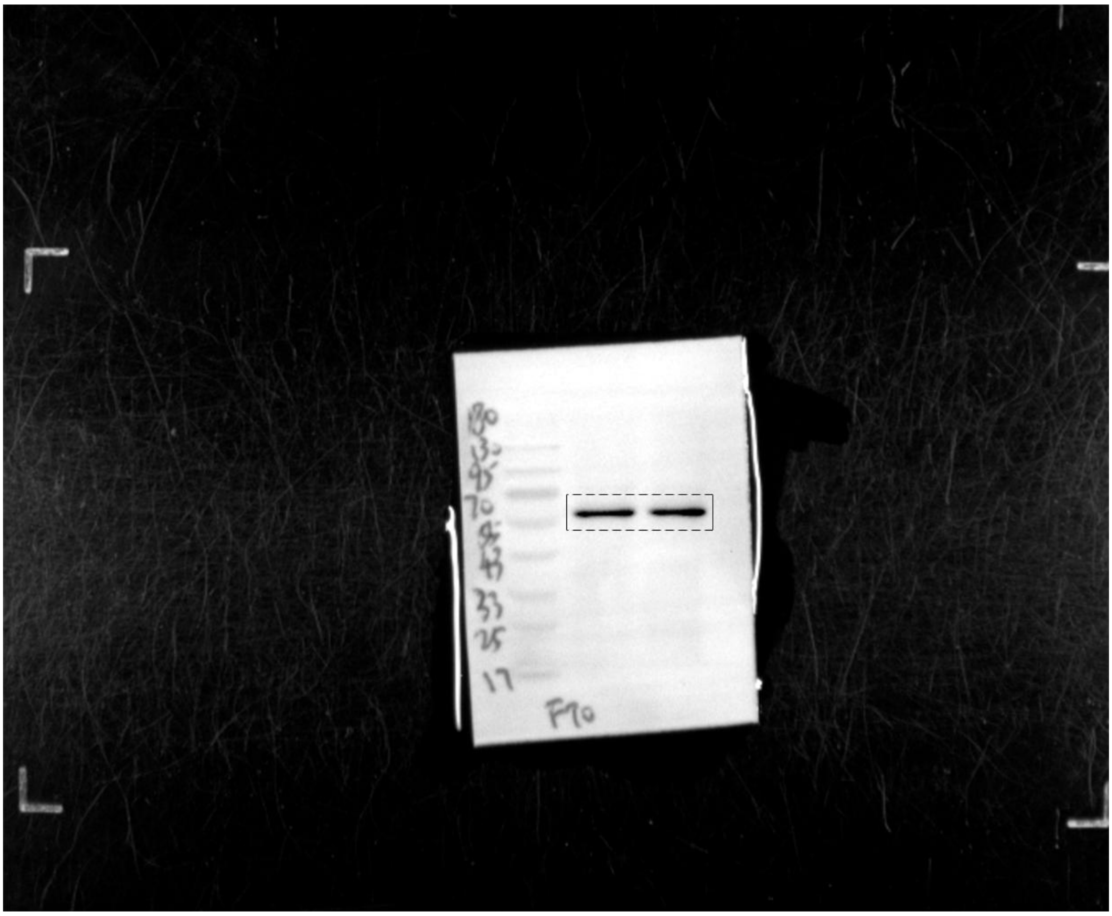

Full unedited gel/blot for Figure 4A-FYN

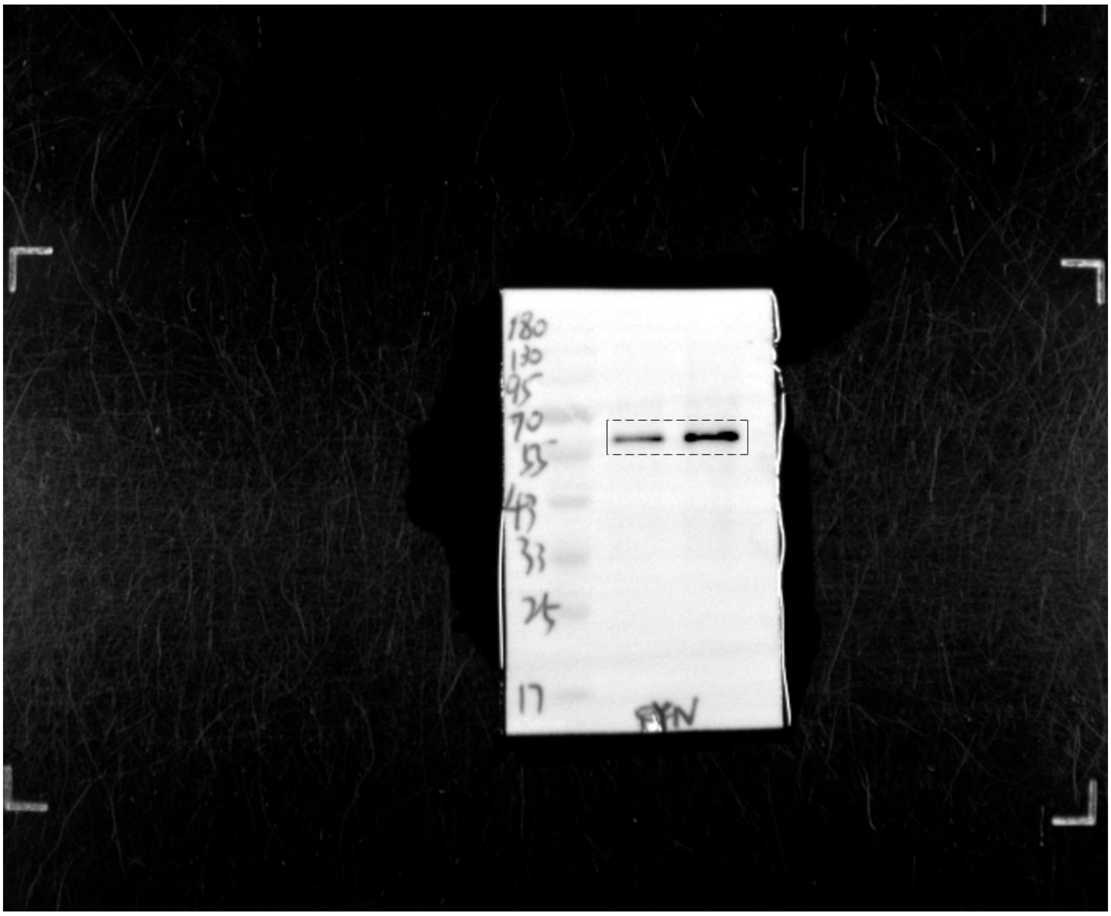

Full unedited gel/blot for Figure 4A-β-actin

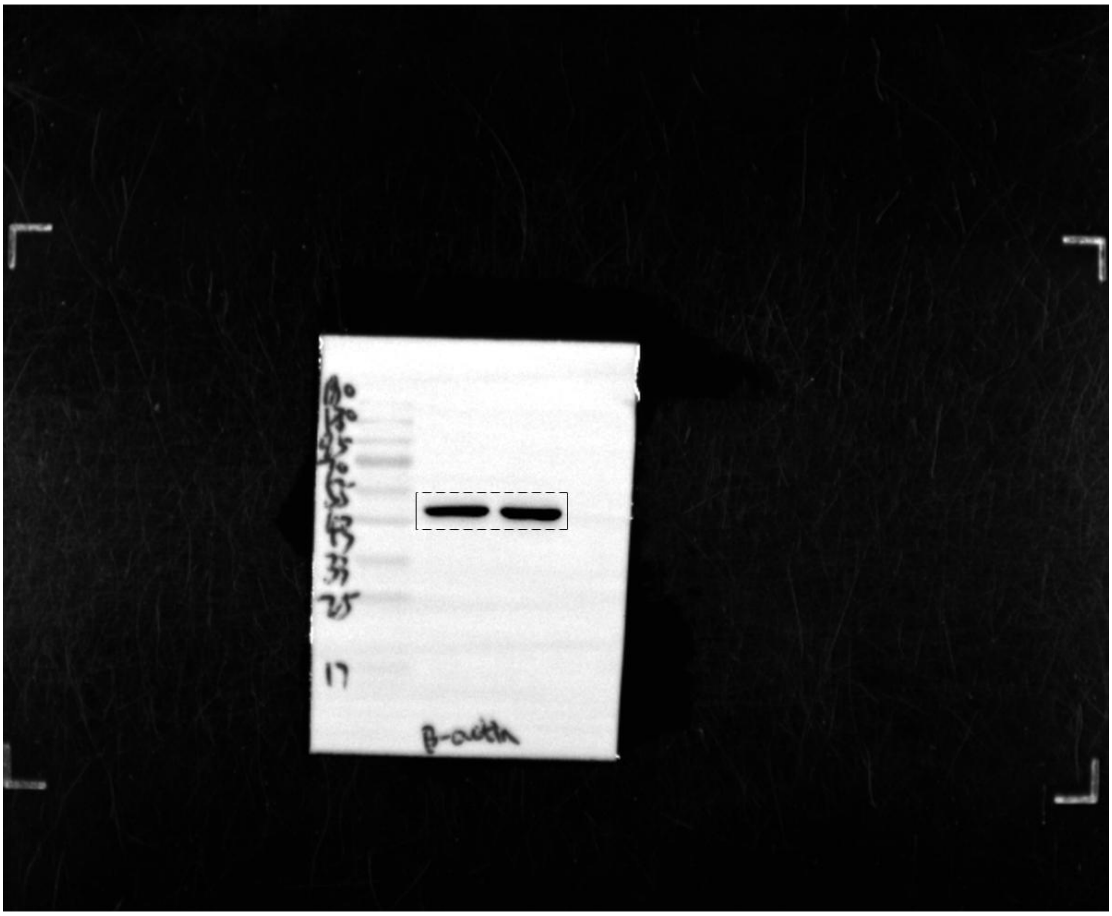

Full unedited gel/blot for Figure 4J-4-HNE

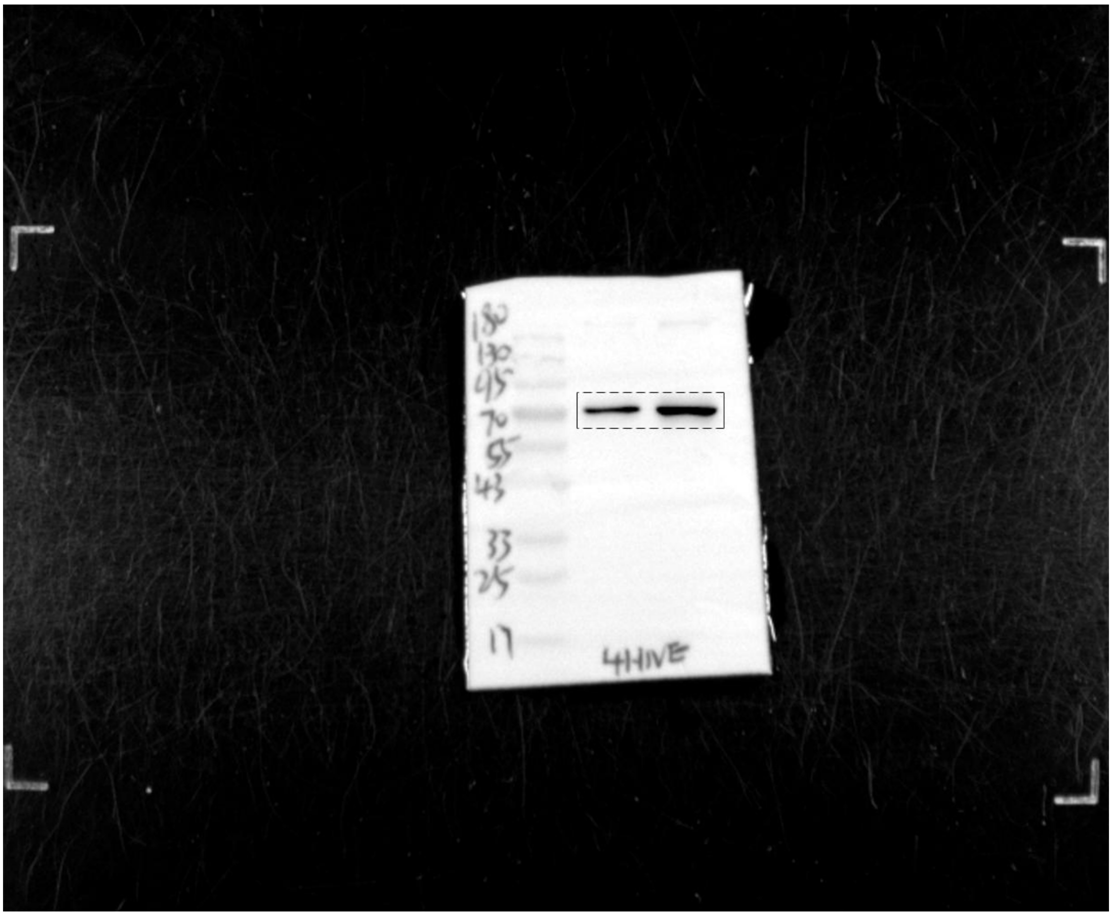

Full unedited gel/blot for Figure 4J-GPX4

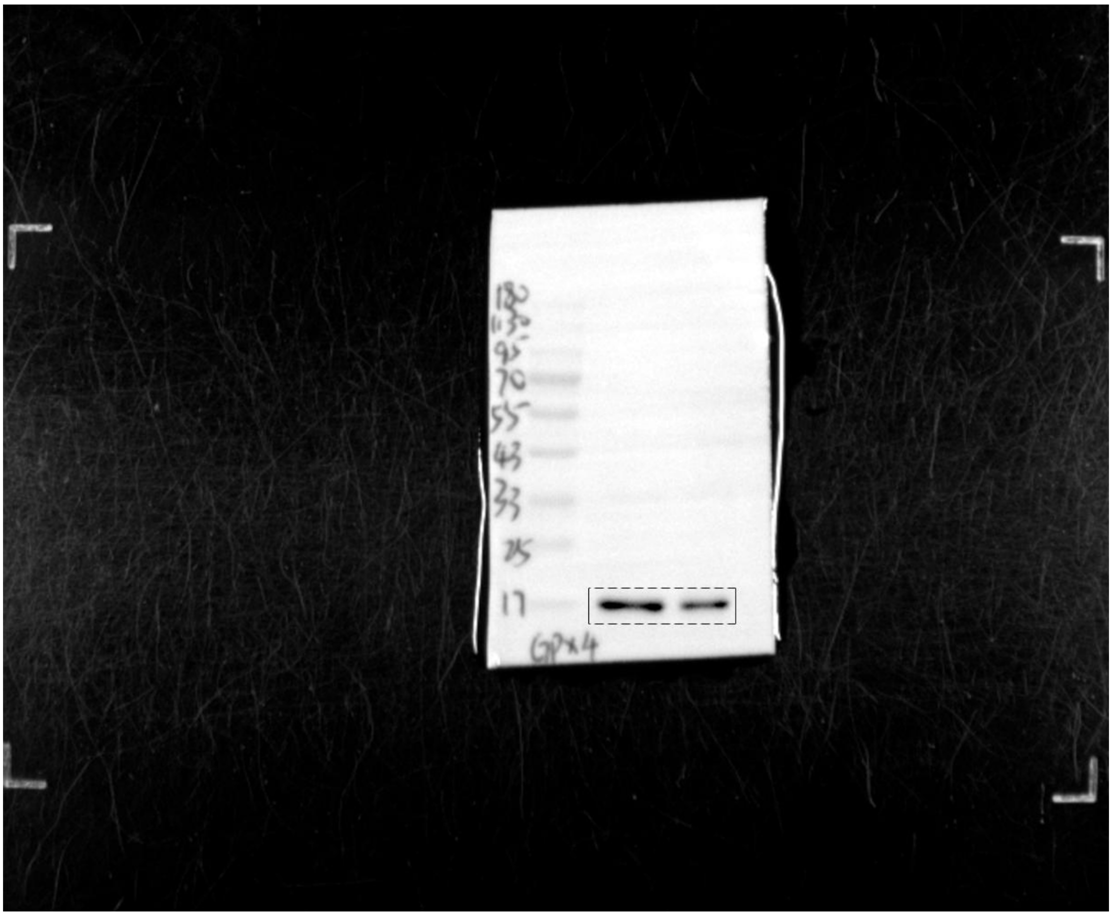

Full unedited gel/blot for Figure 4J-β-actin

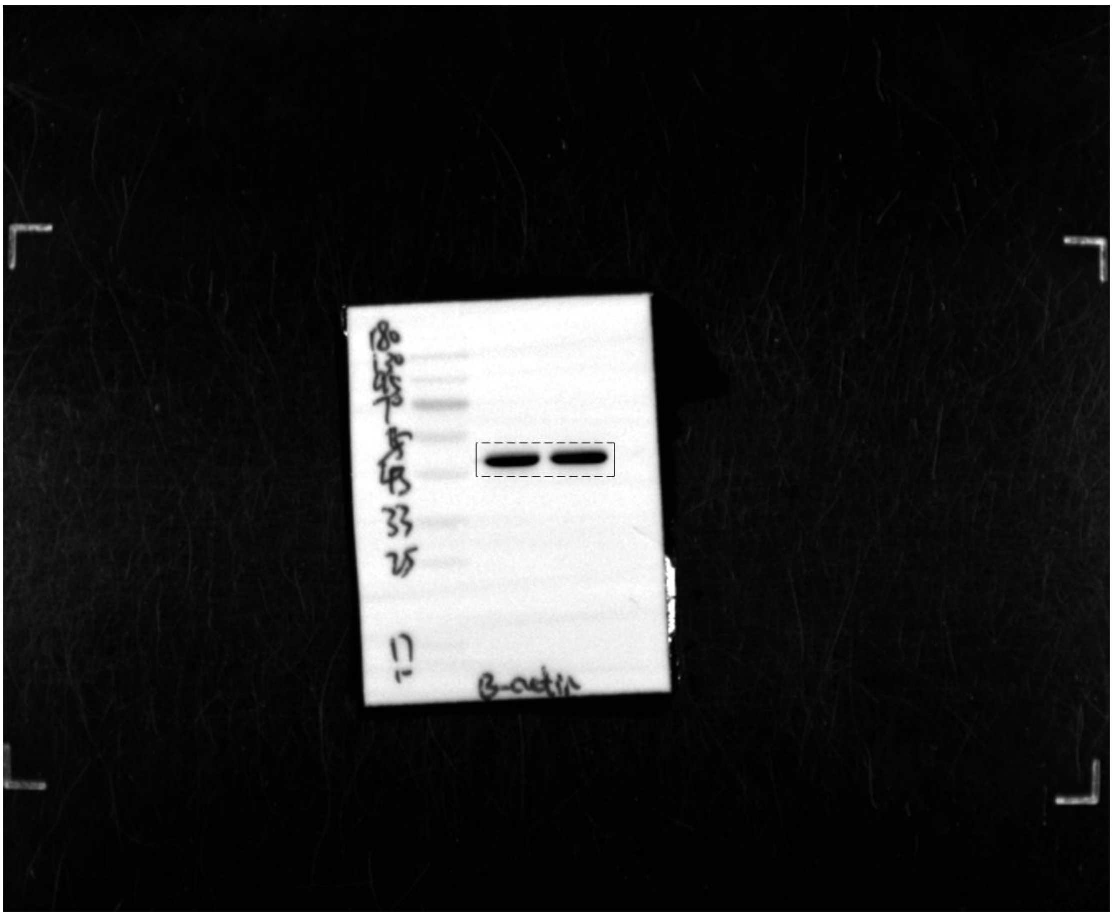

Full unedited gel/blot for Figure 5A-COX IV

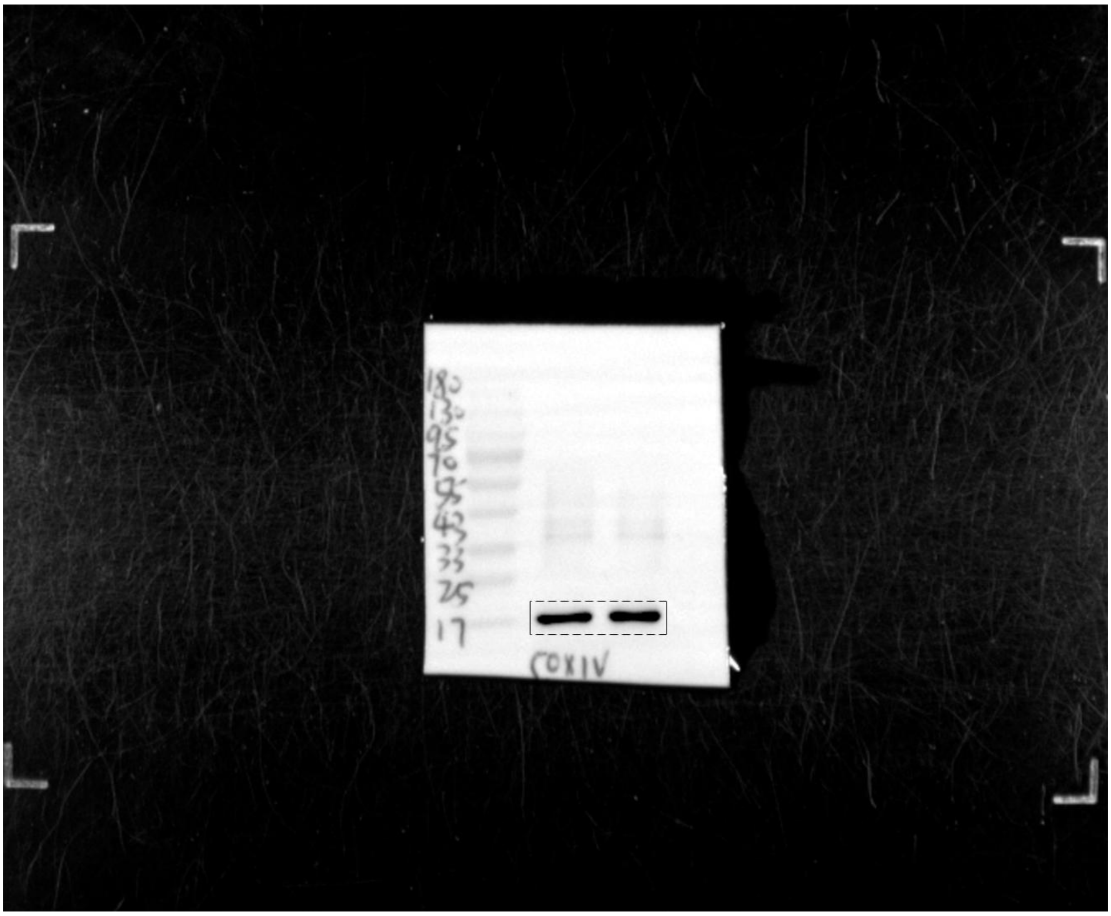

Full unedited gel/blot for Figure 5A-Drp1

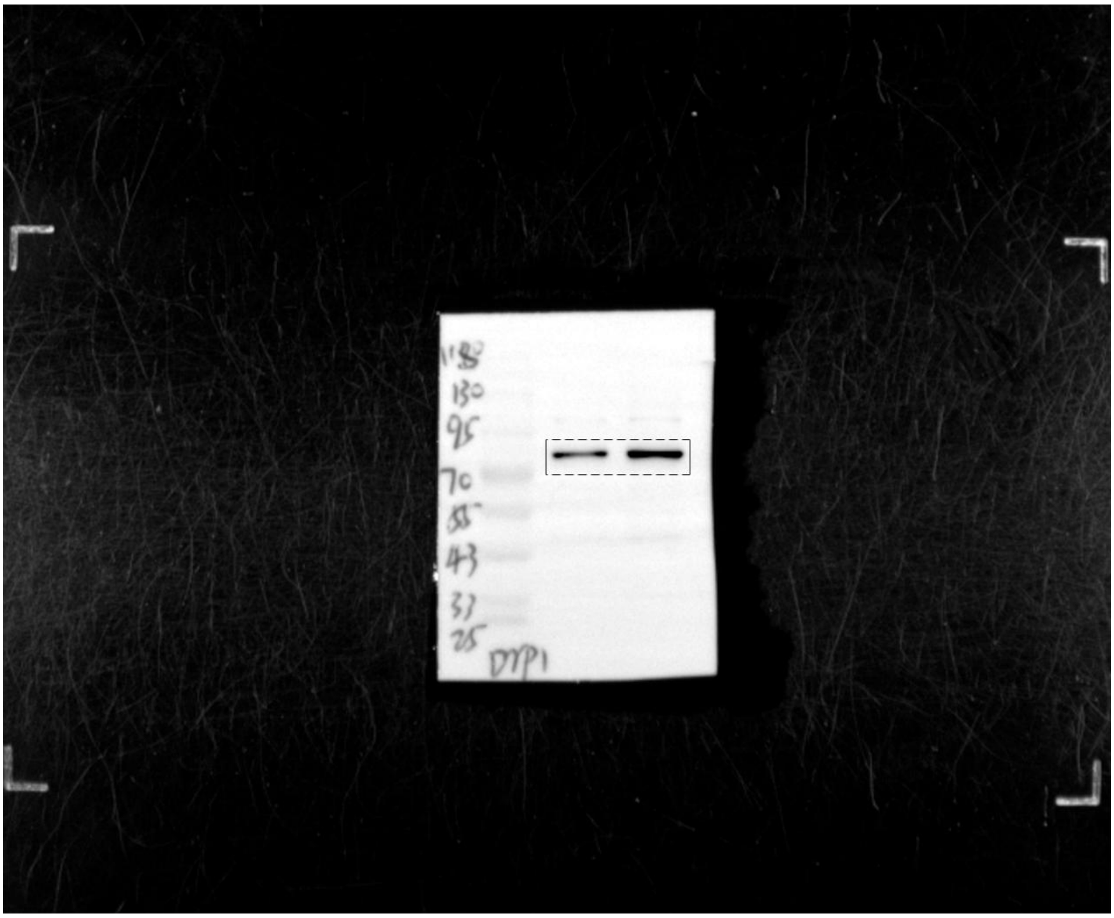

Full unedited gel/blot for Figure 5A-p-Drp1

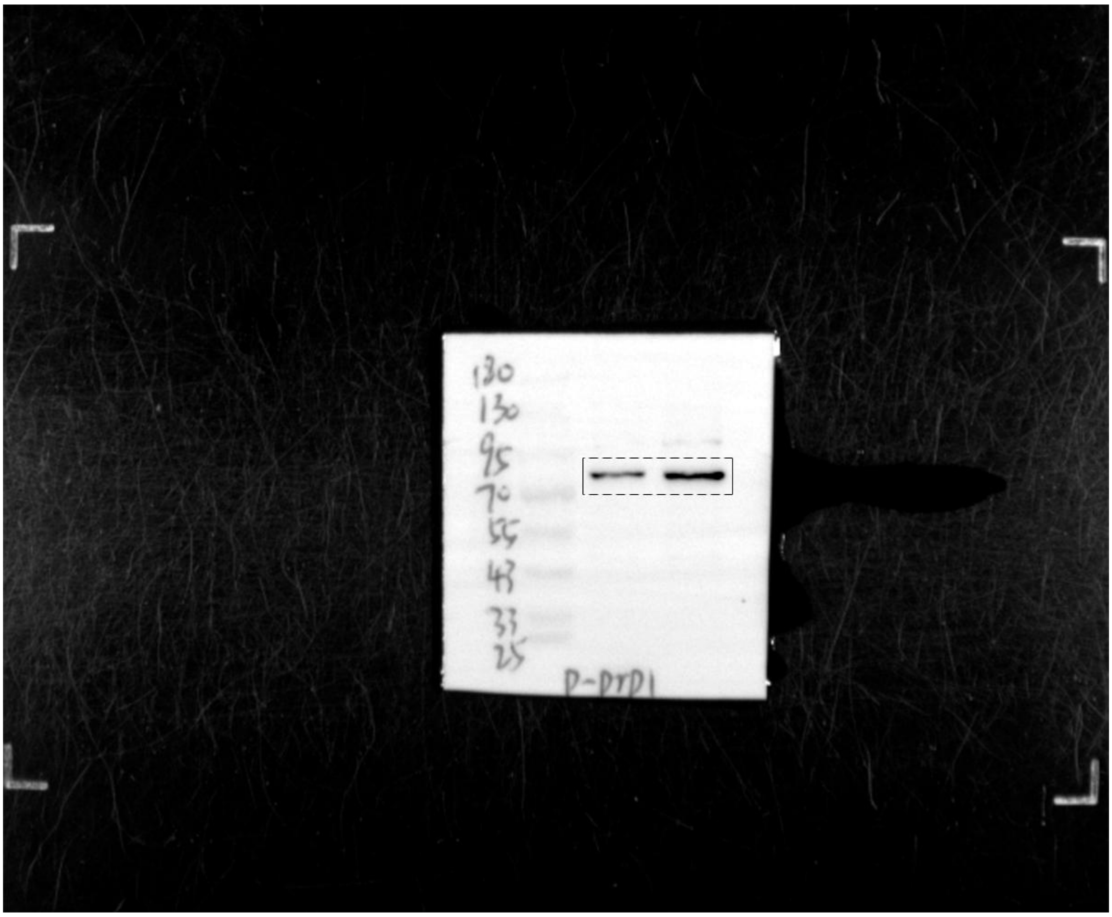

Full unedited gel/blot for Figure 5A-β-actin

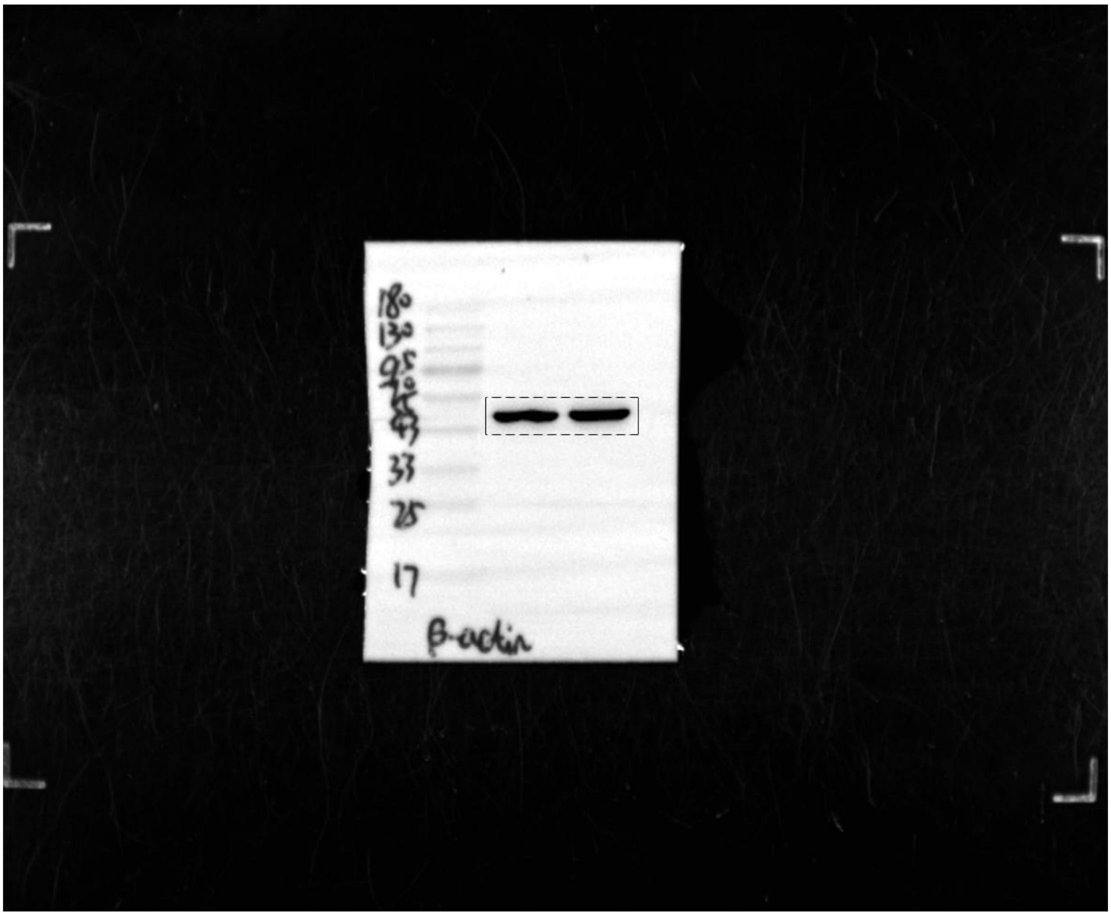

Full unedited gel/blot for Figure 5B-Drp1 IP:Fyn

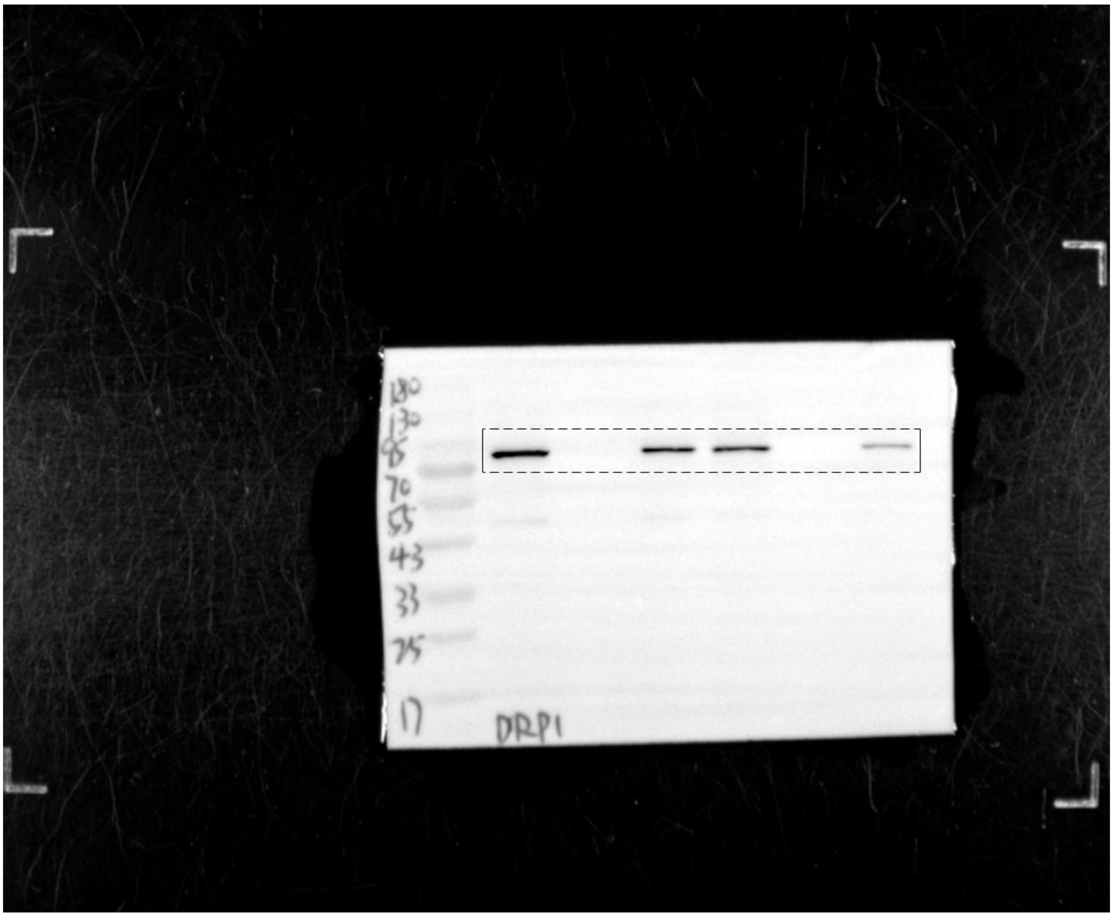

Full unedited gel/blot for Figure 5B-Drp1 IP:Drp1

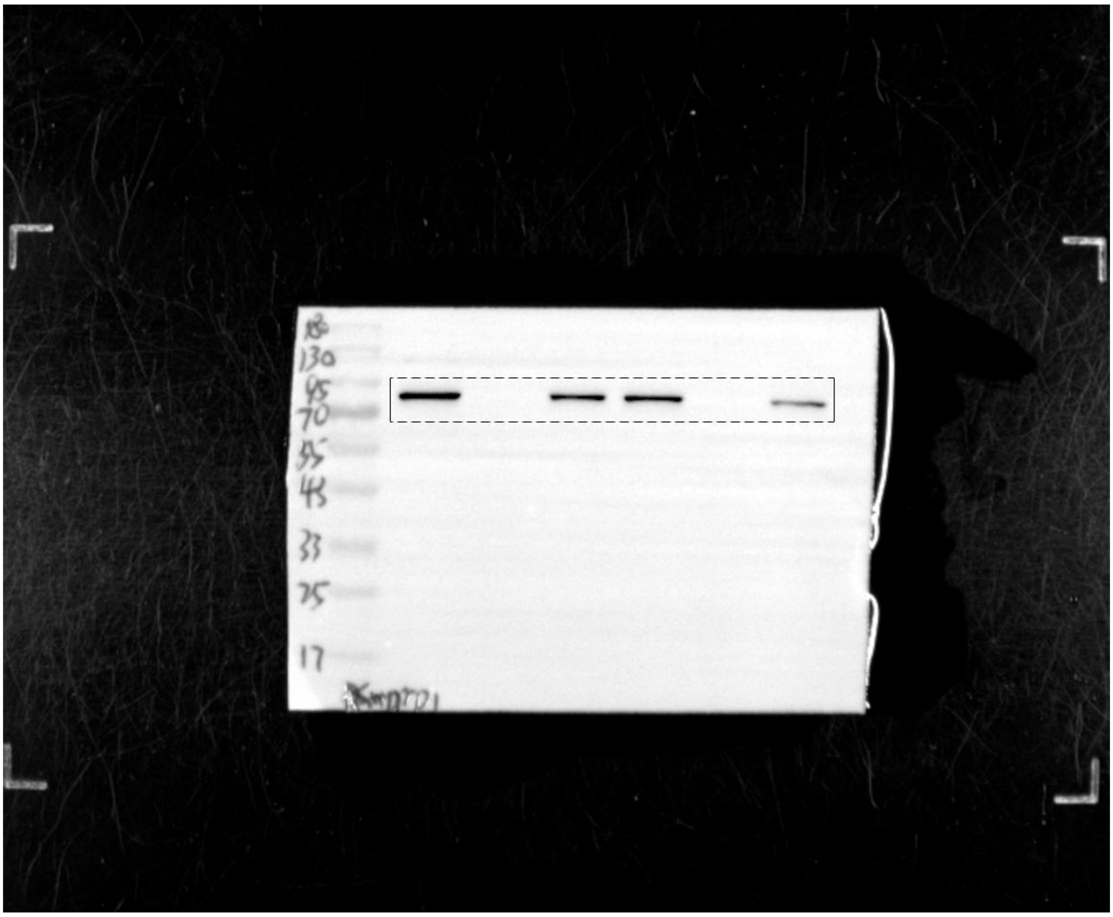

Full unedited gel/blot for Figure 5B-FYN IP:FYN

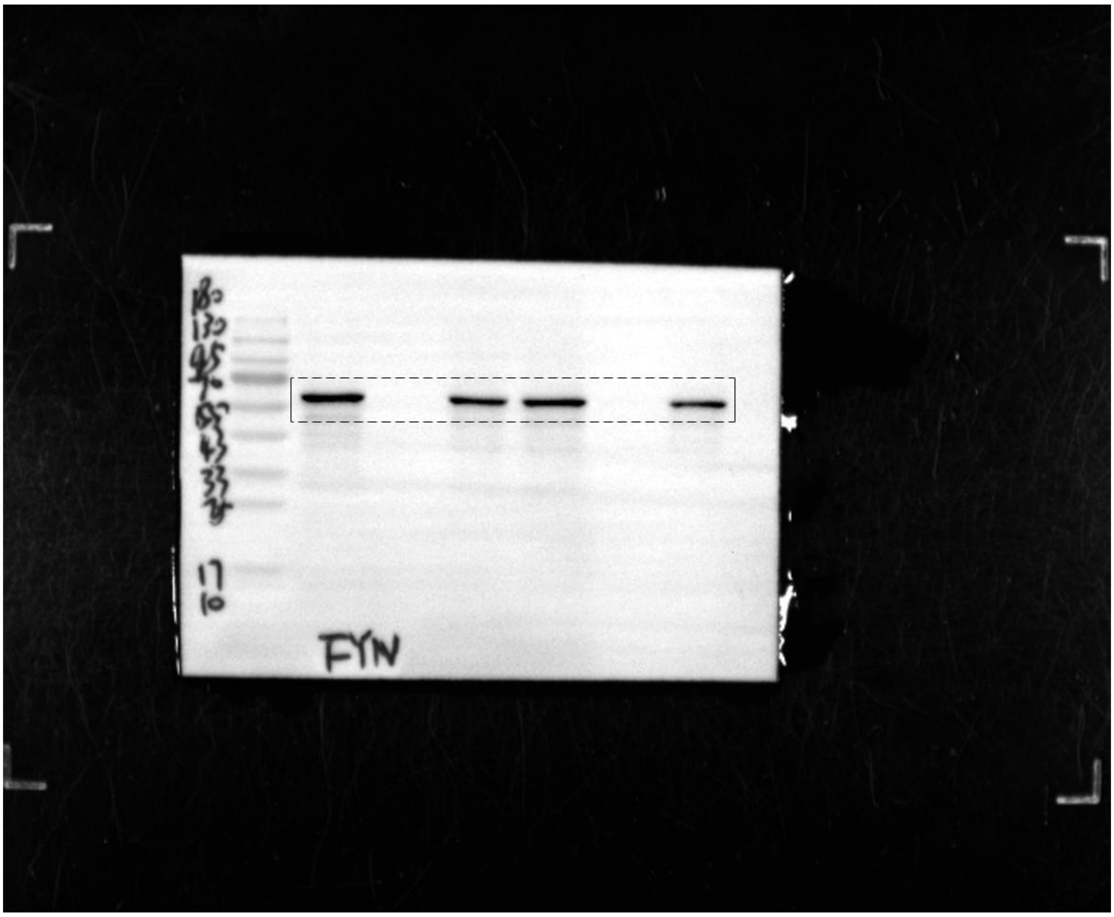

Full unedited gel/blot for Figure 5B-FYN IP:Drp1

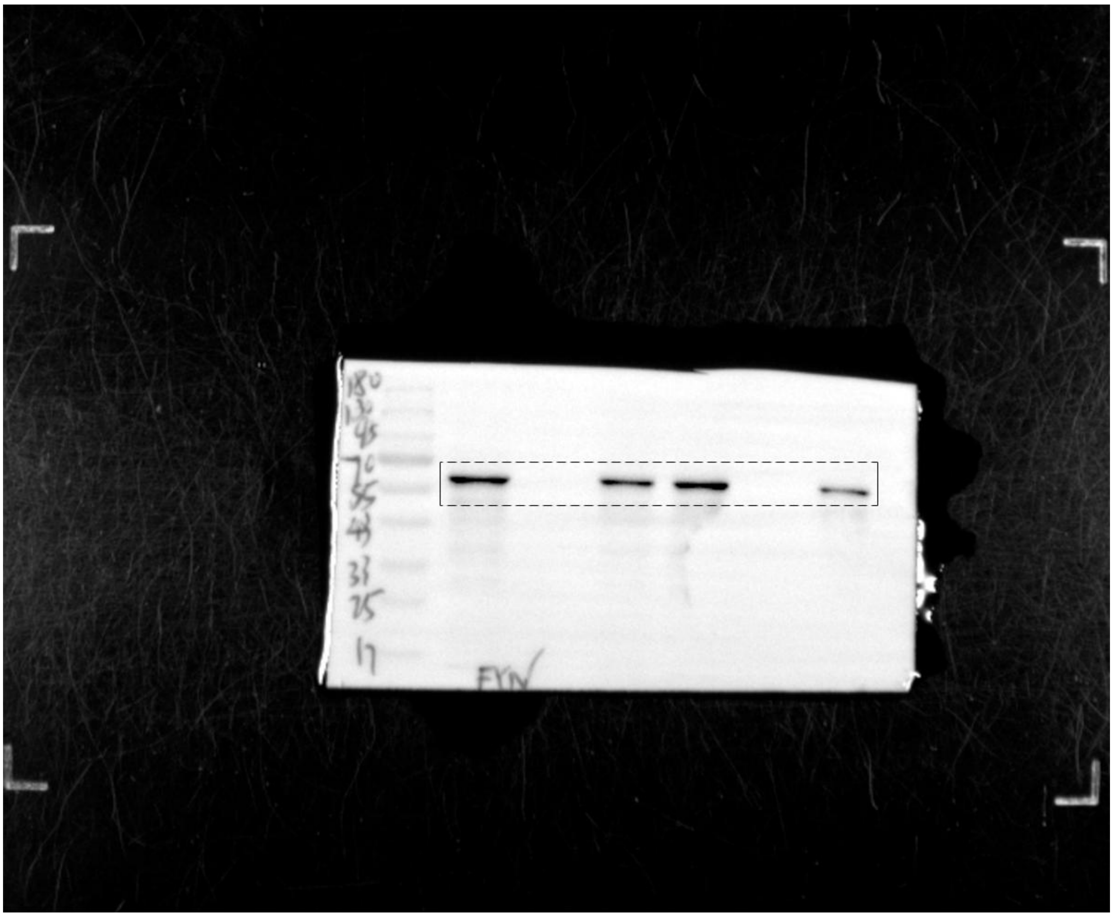

Full unedited gel/blot for Figure 5B-p-Drp1 IP:FYN

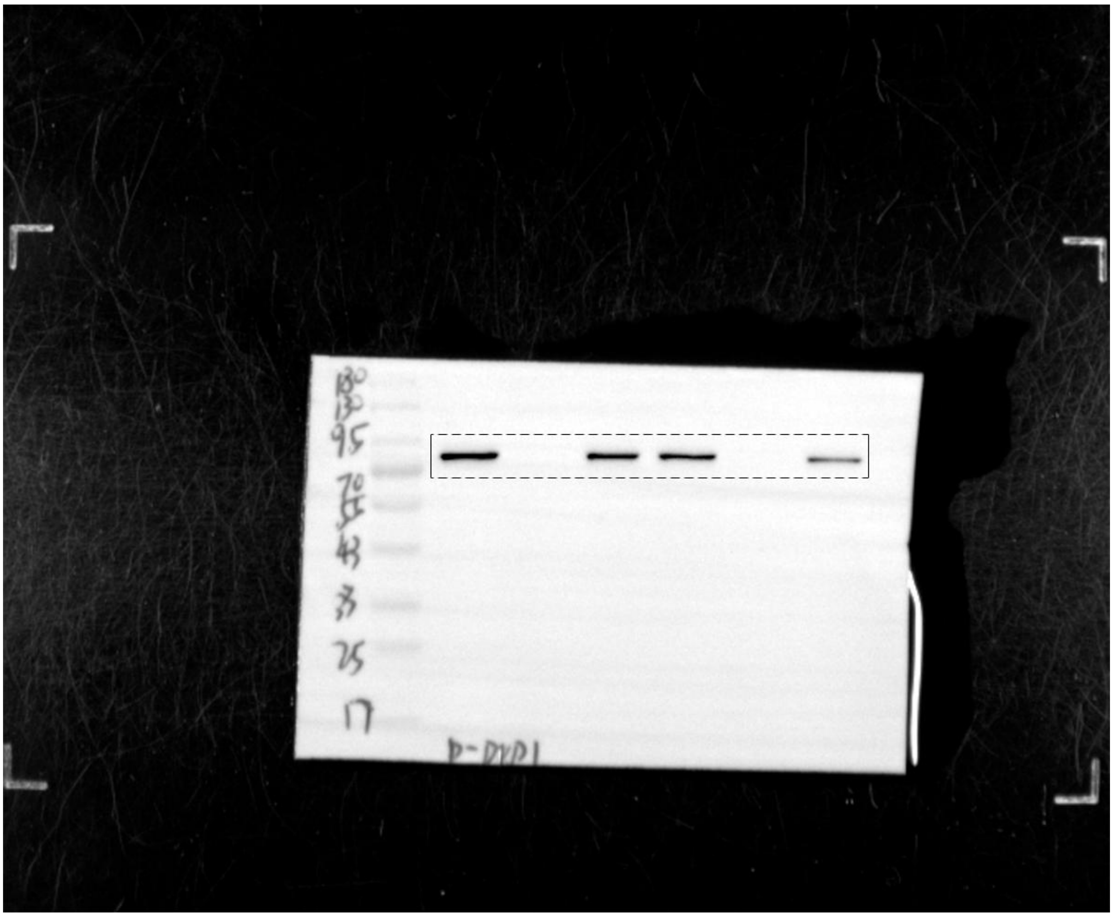

Full unedited gel/blot for Figure 5D-COX IV

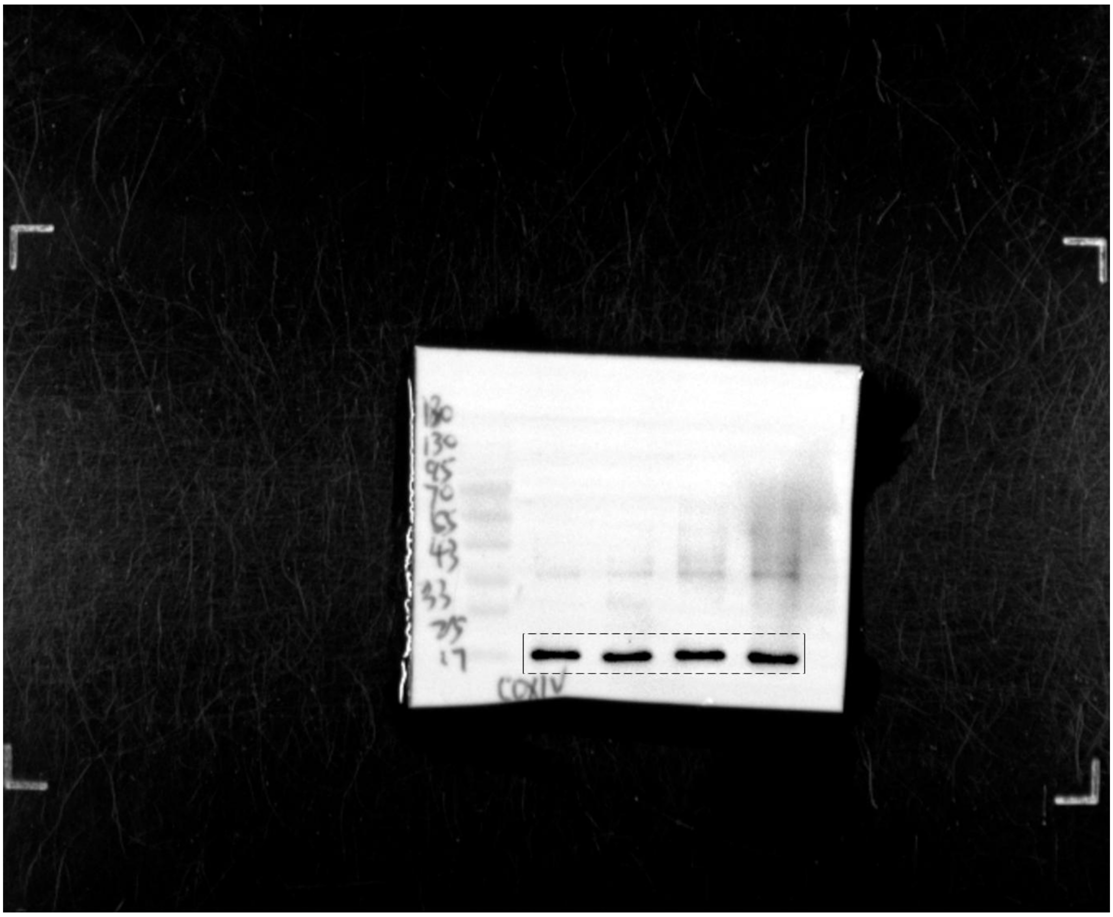

Full unedited gel/blot for Figure 5D-Drp1

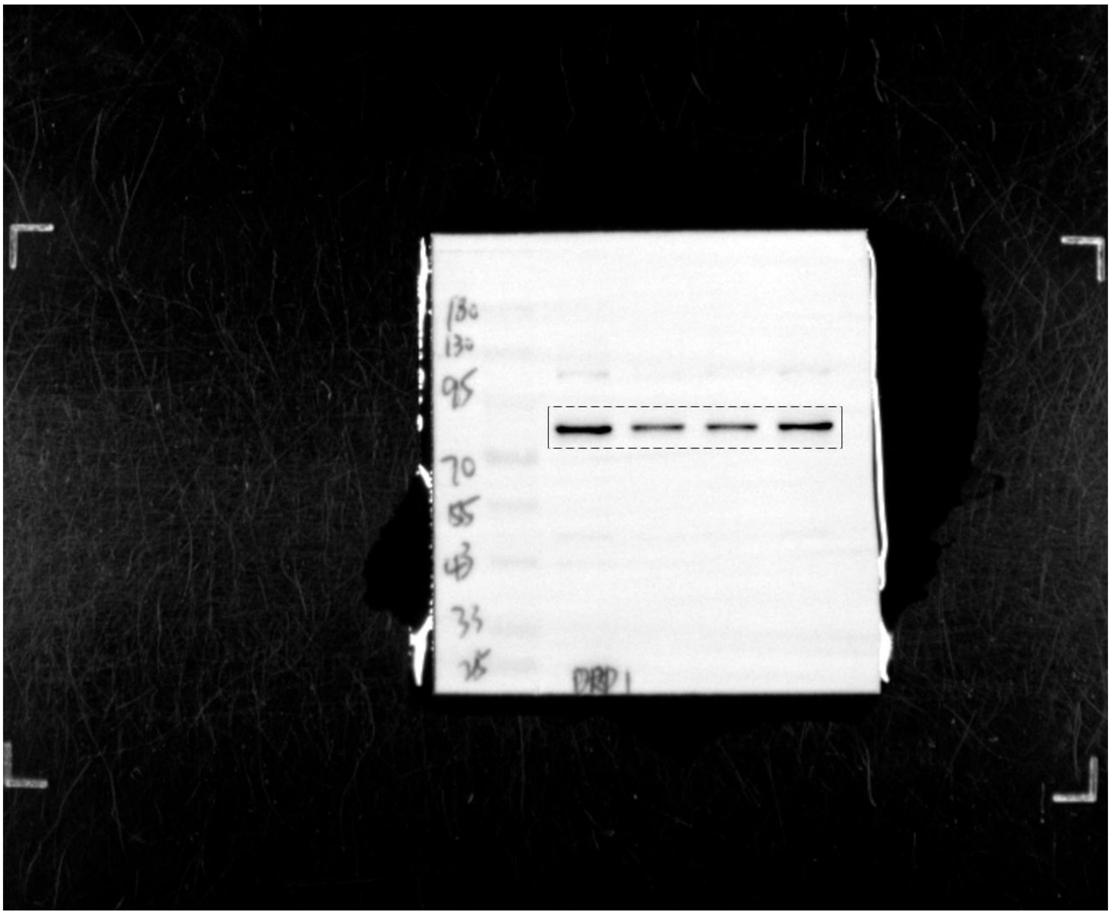

Full unedited gel/blot for Figure 5D-FTO

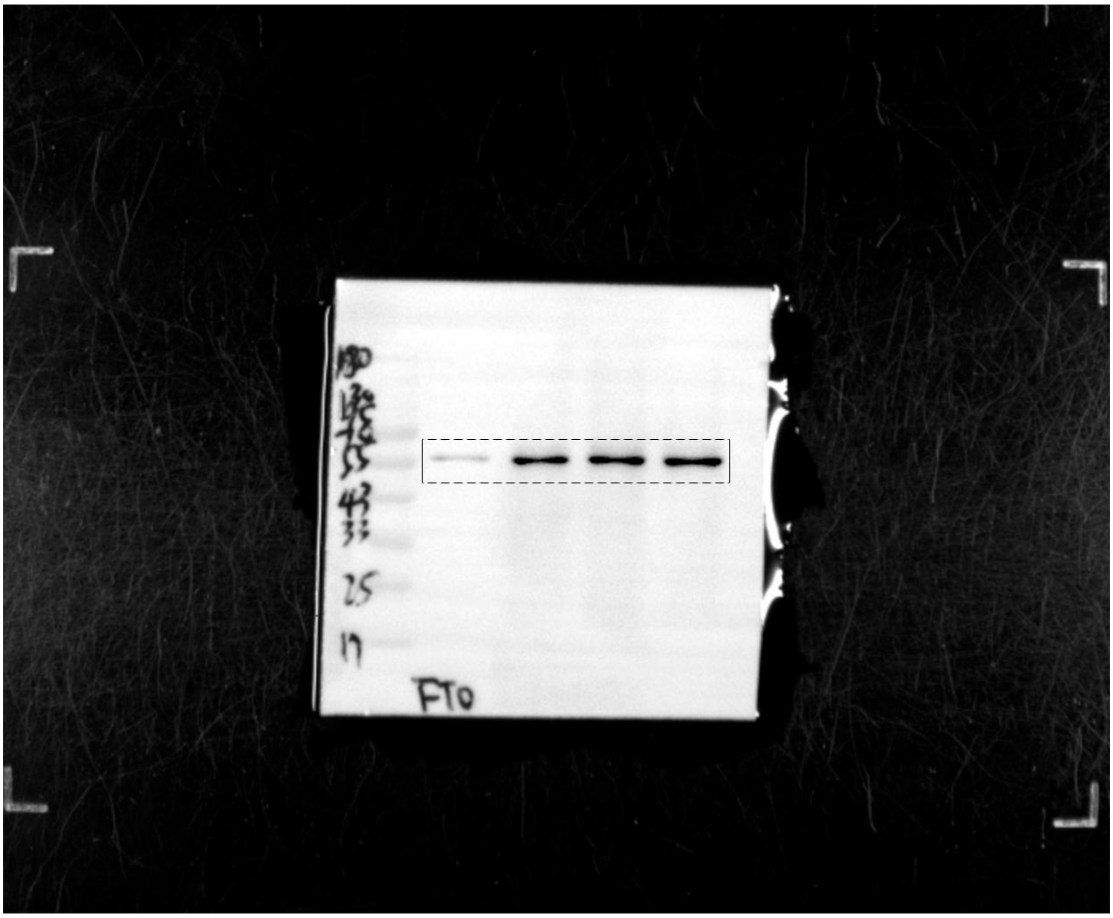

Full unedited gel/blot for Figure 5D-FYN

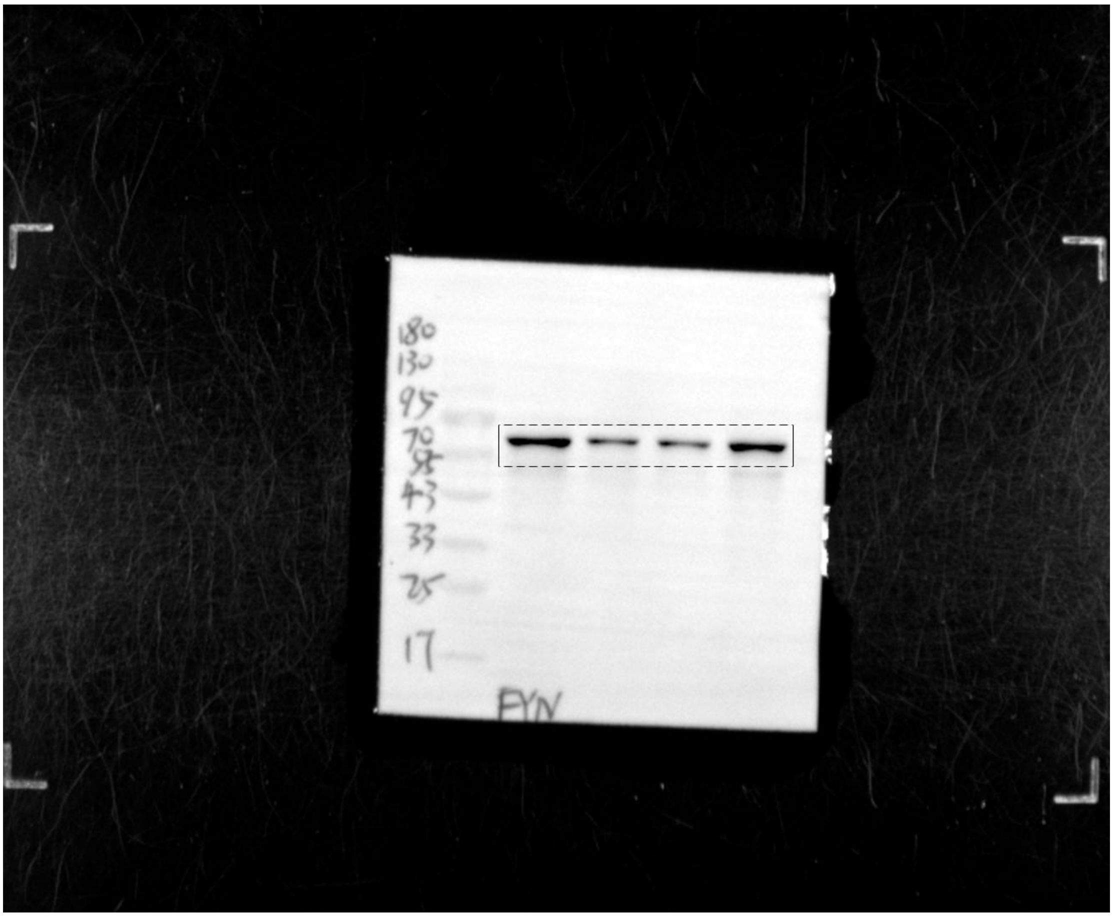

Full unedited gel/blot for Figure 5D-p-Drp1

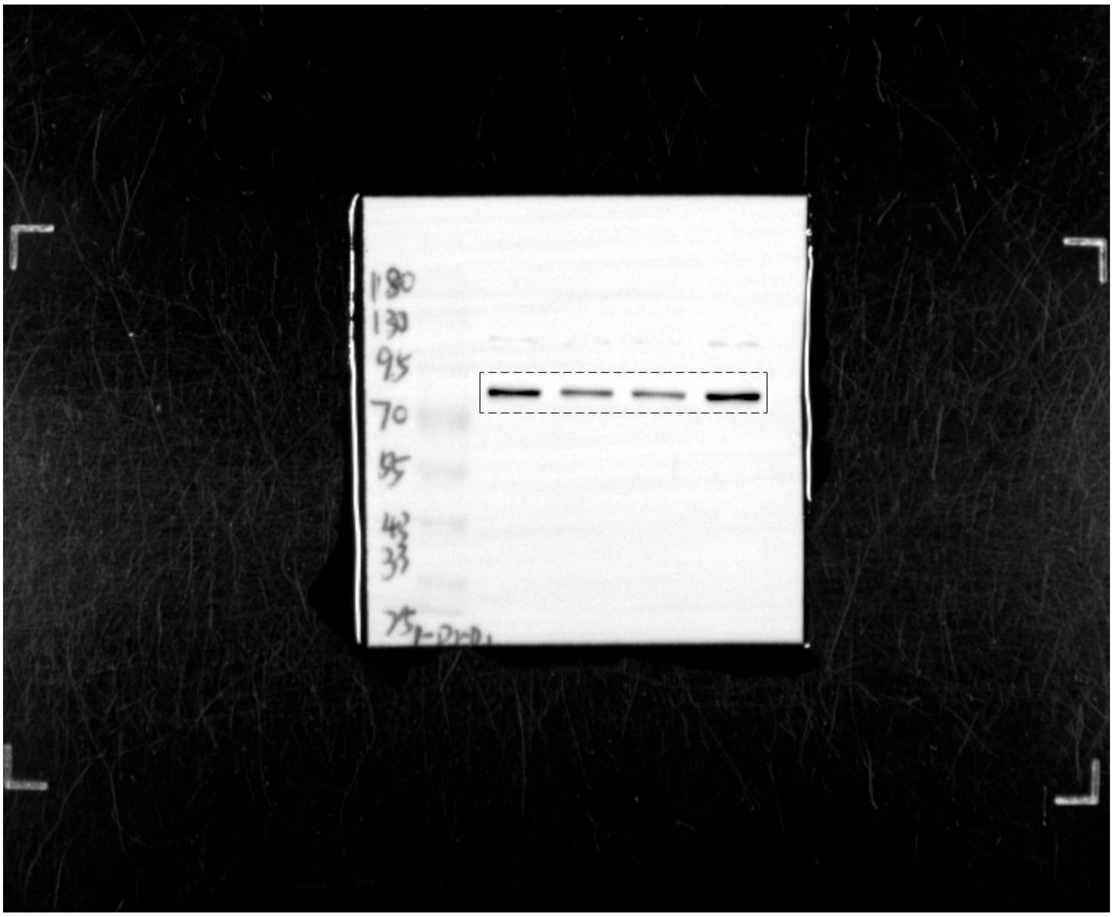

Full unedited gel/blot for Figure 5D-β-actin

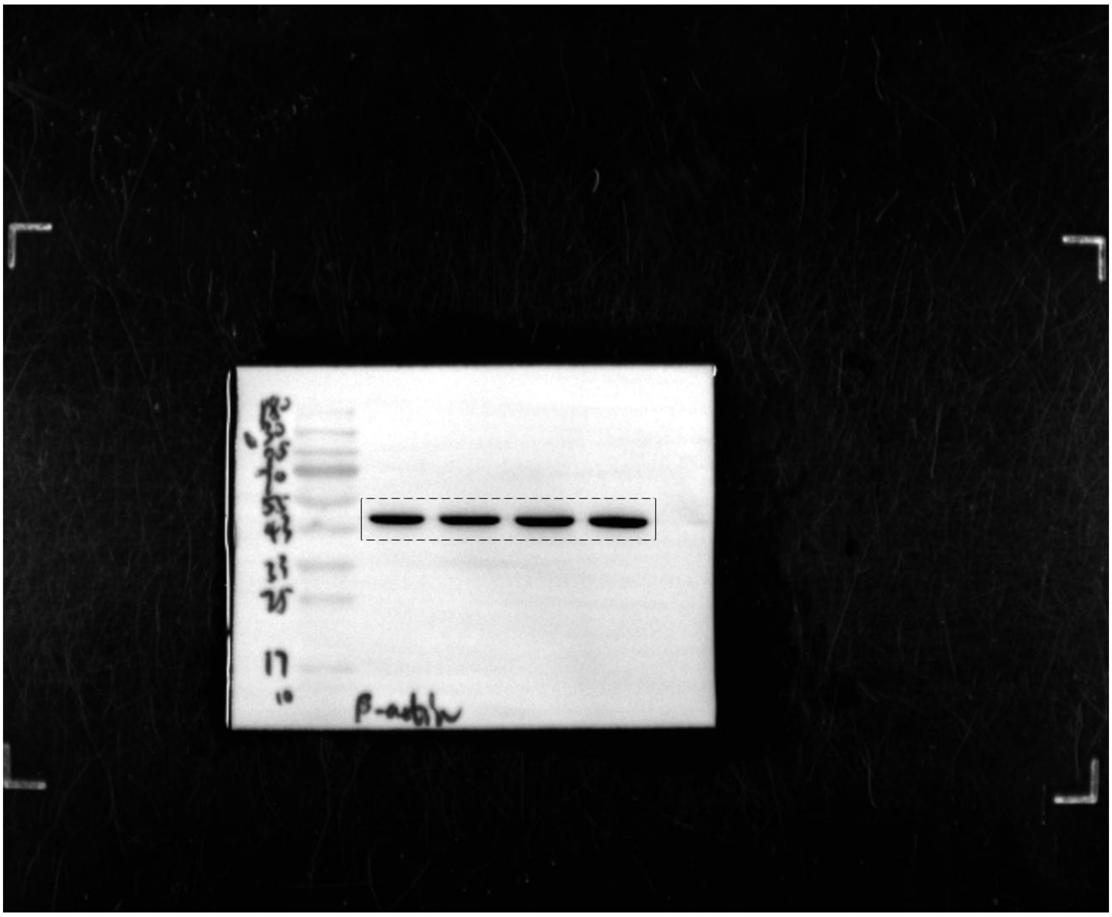

Full unedited gel/blot for Figure 6A-COX IV

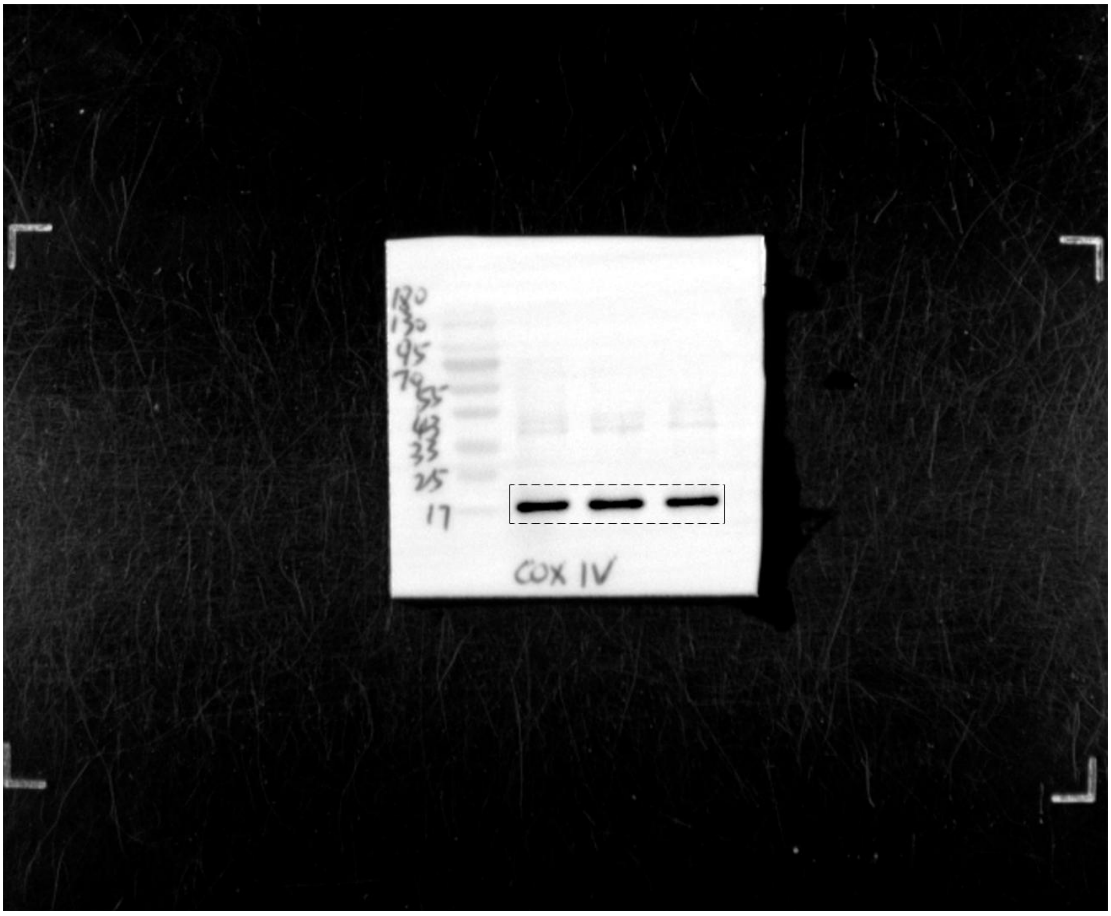

Full unedited gel/blot for Figure 6A-Drp1

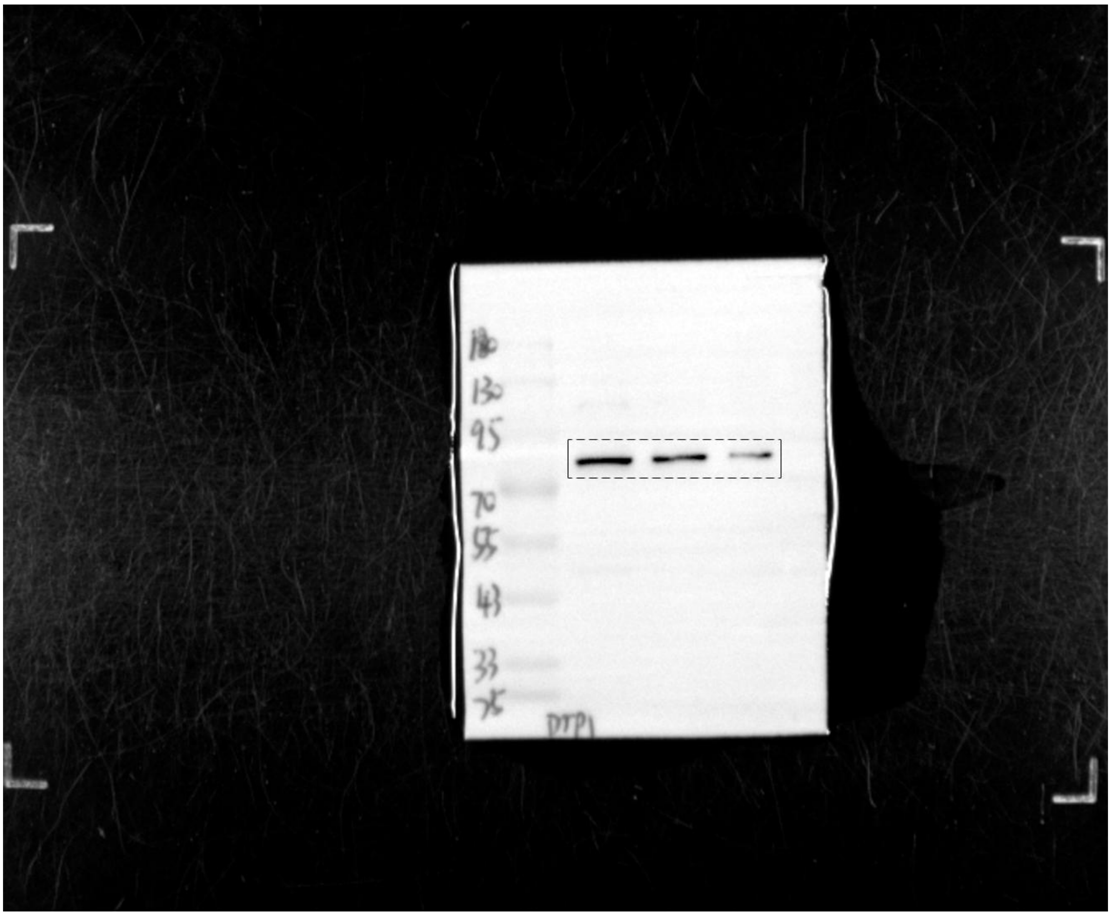

Full unedited gel/blot for Figure 6A-FTO

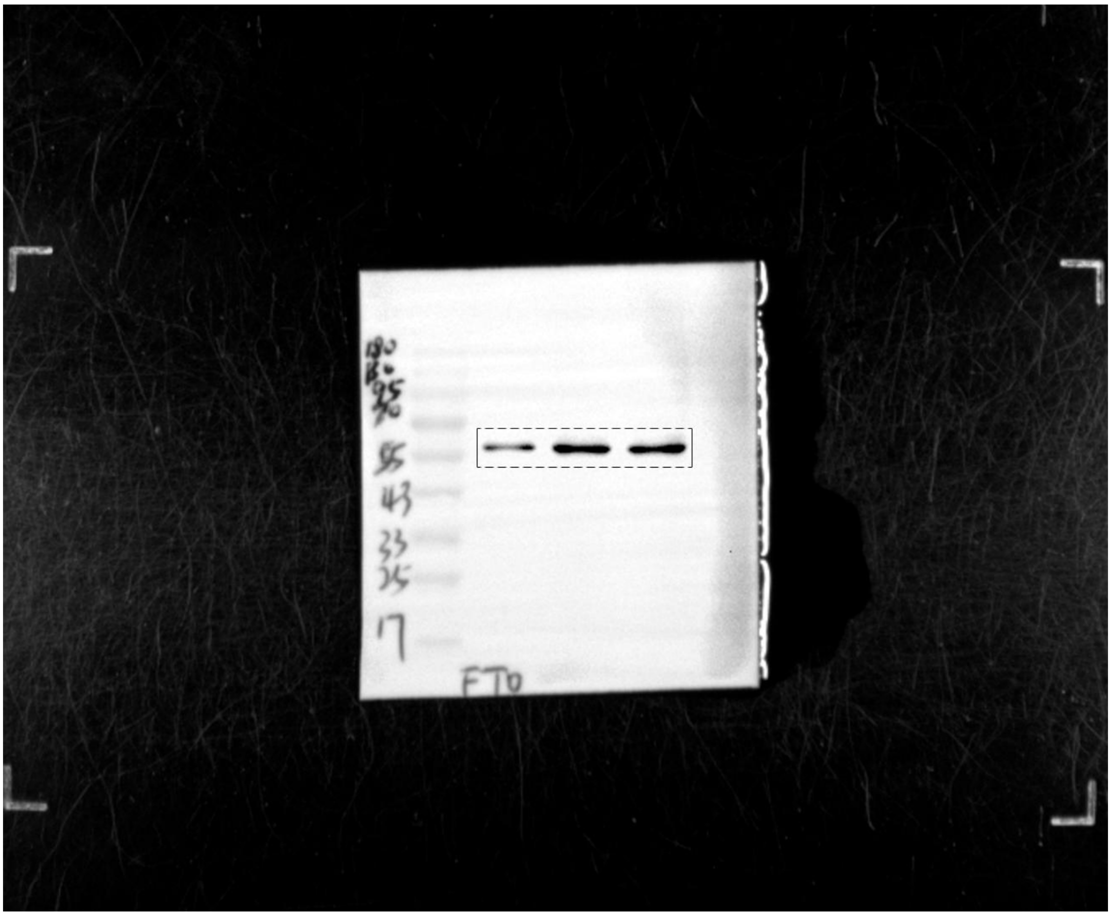

Full unedited gel/blot for Figure 6A-FYN

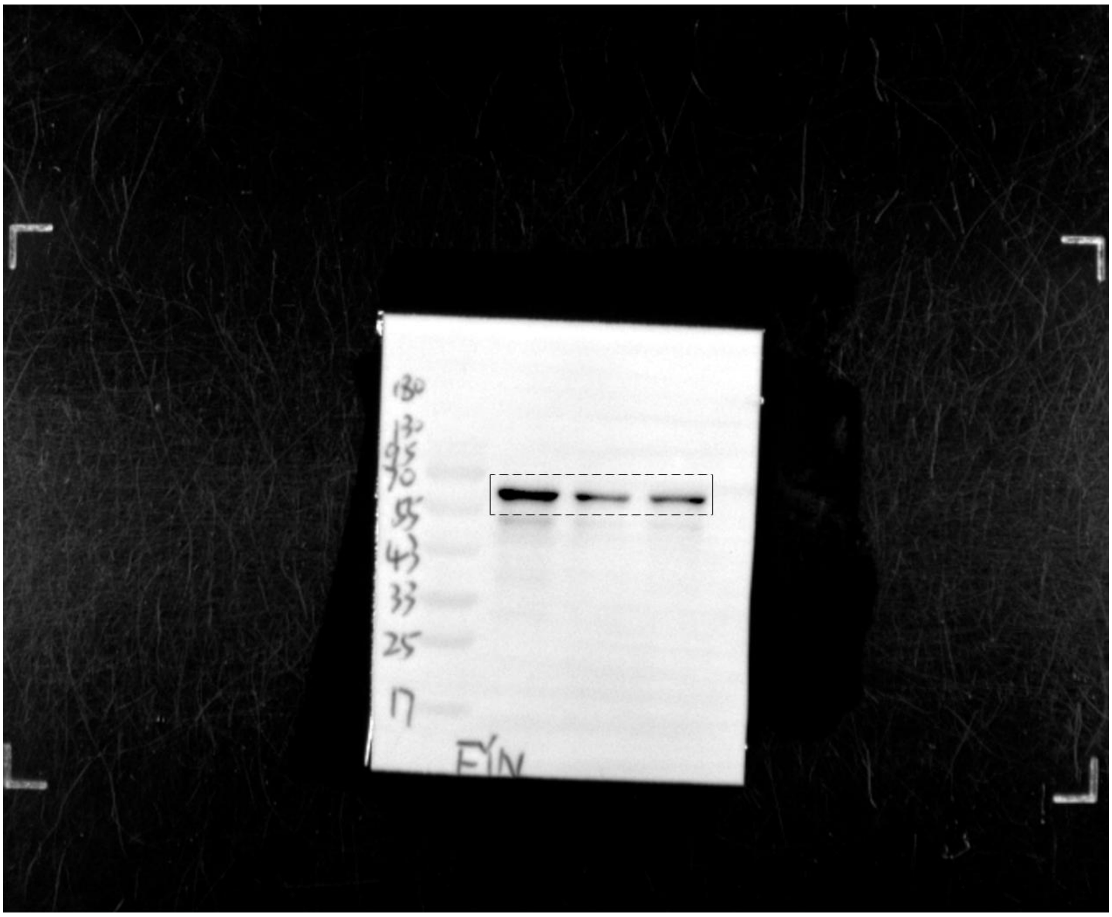

Full unedited gel/blot for Figure 6A-p-Drp1

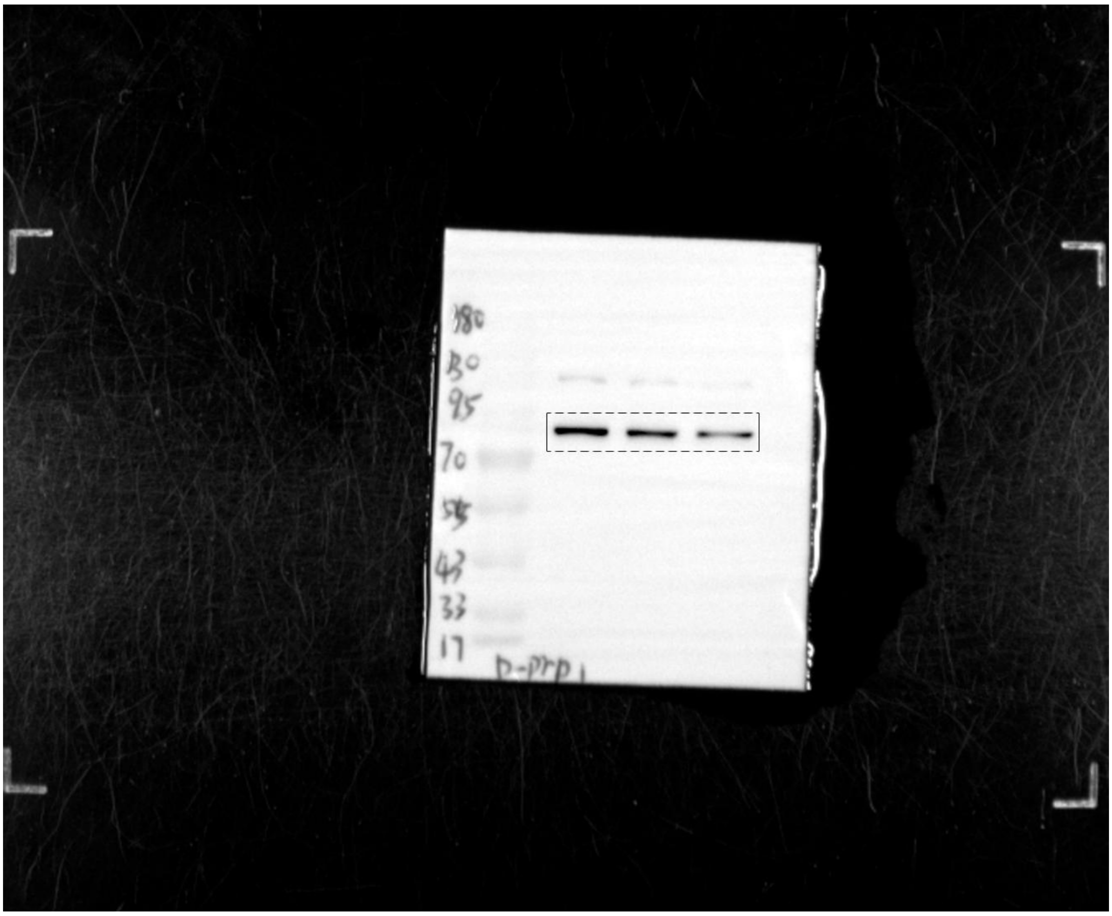

Full unedited gel/blot for Figure 6A-β-actin

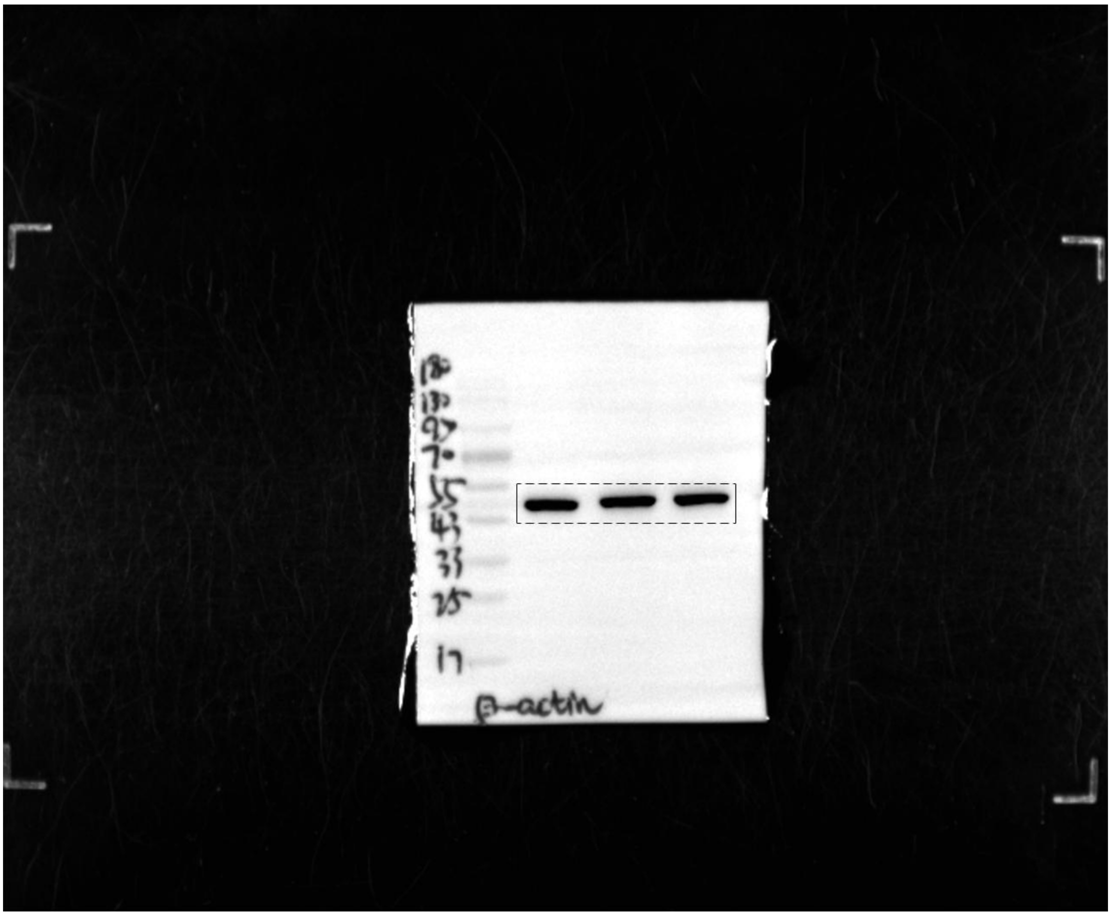

Full unedited gel/blot for Figure 6J-4-HNE

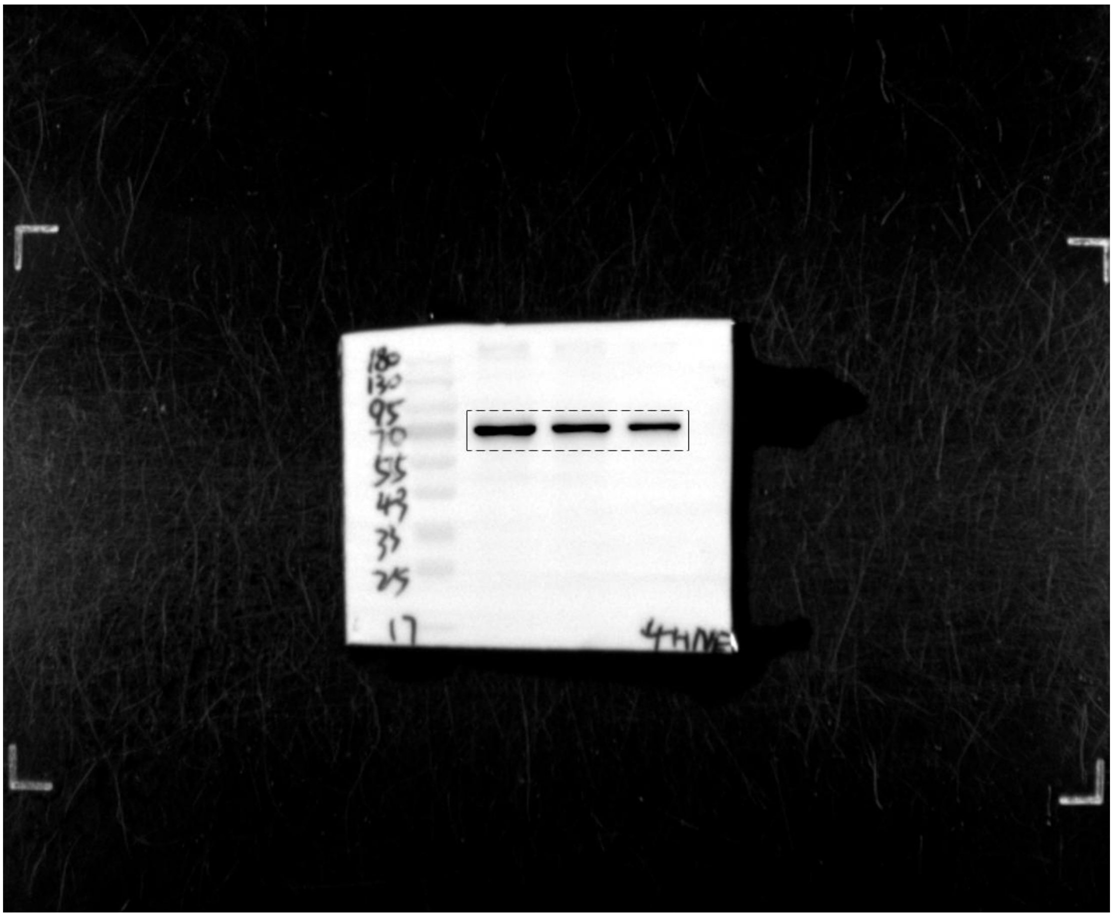

Full unedited gel/blot for Figure 6J-GPX4

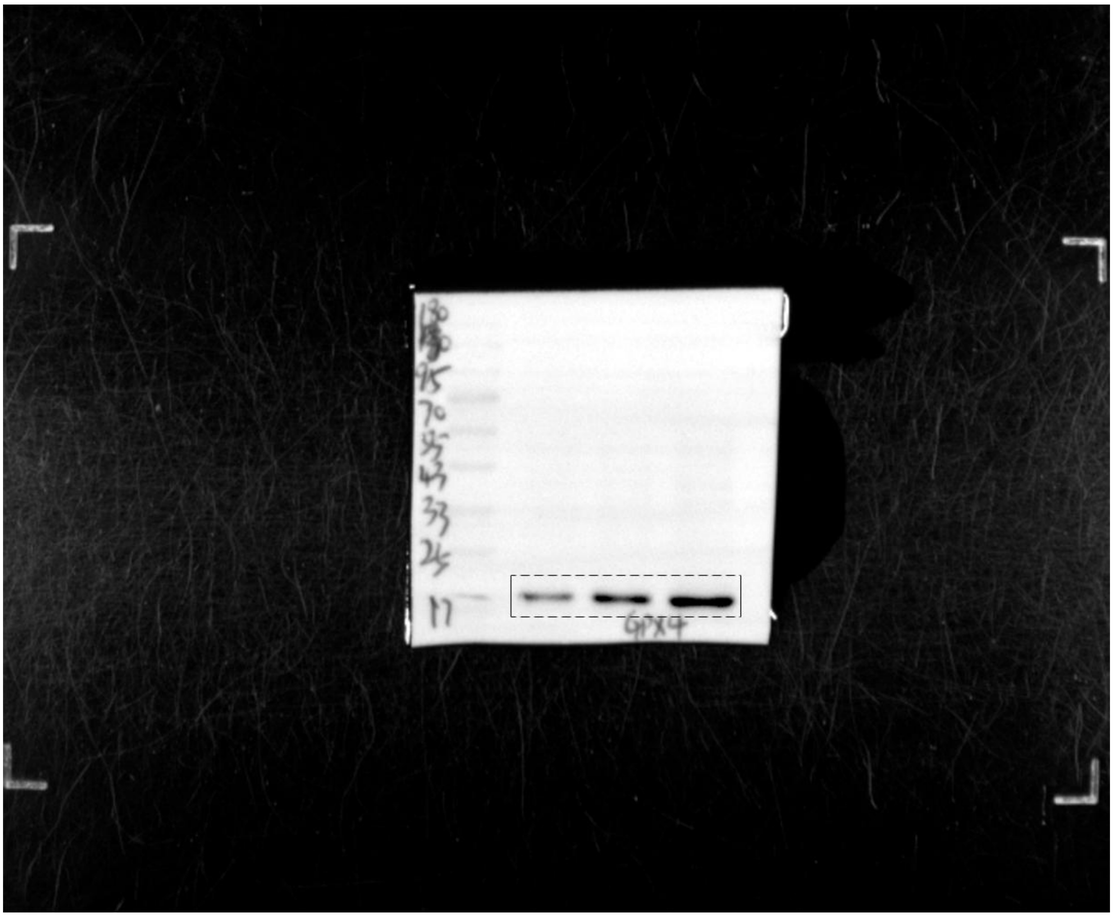

Full unedited gel/blot for Figure 6J-β-actin

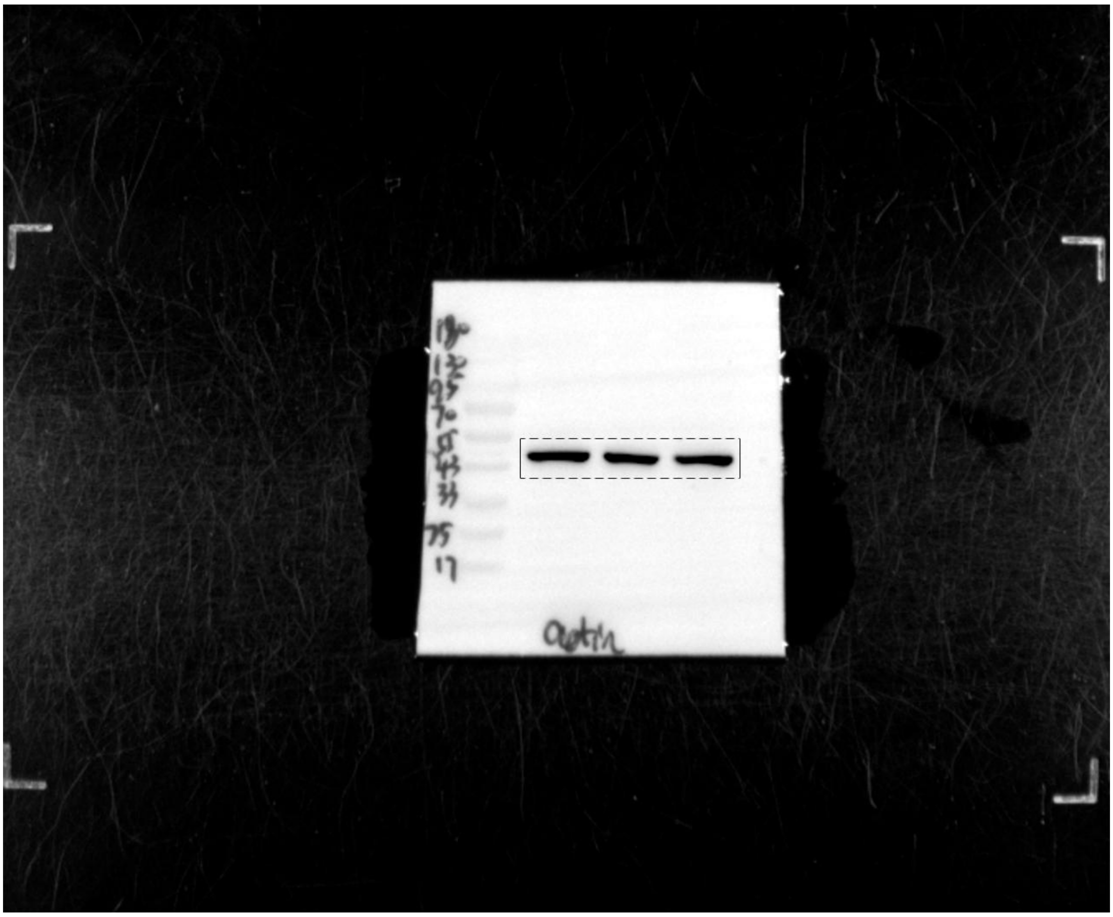

Full unedited gel/blot for Figure 7A-COX IV

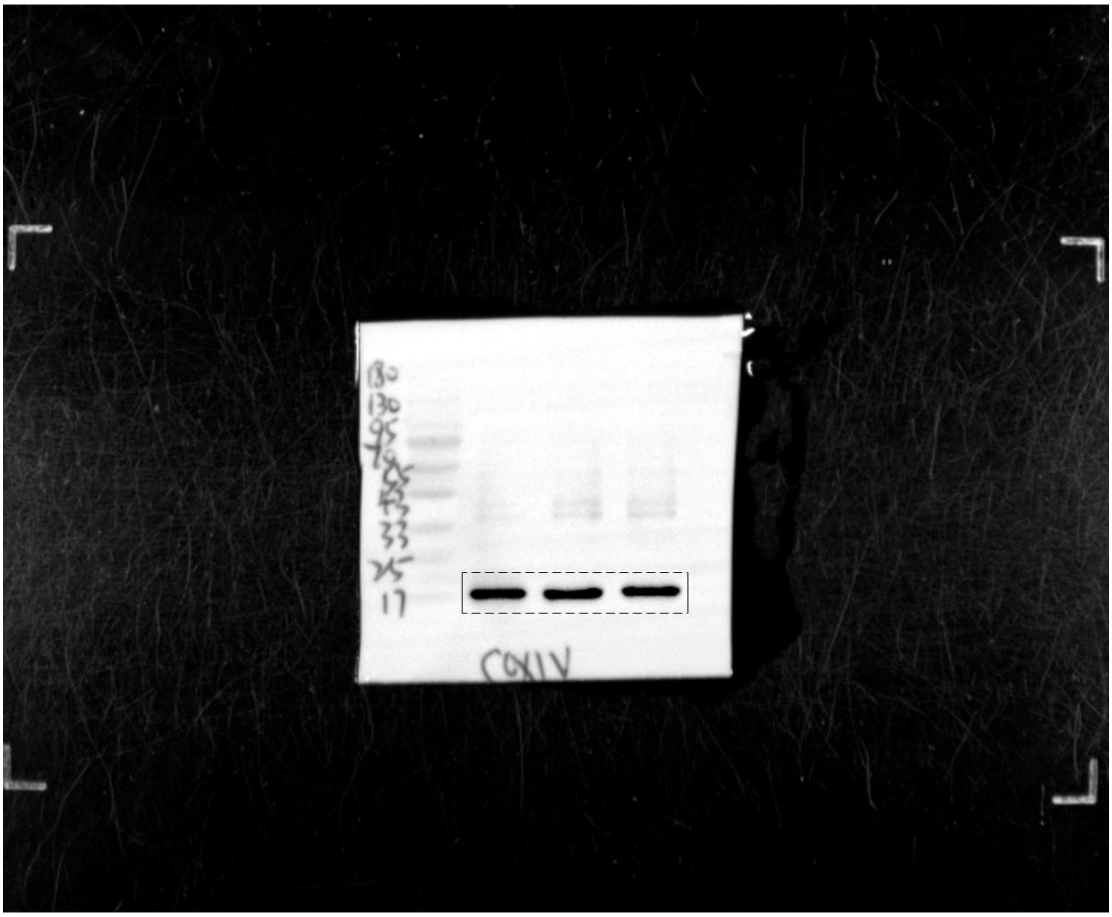

Full unedited gel/blot for Figure 7A-Drp1

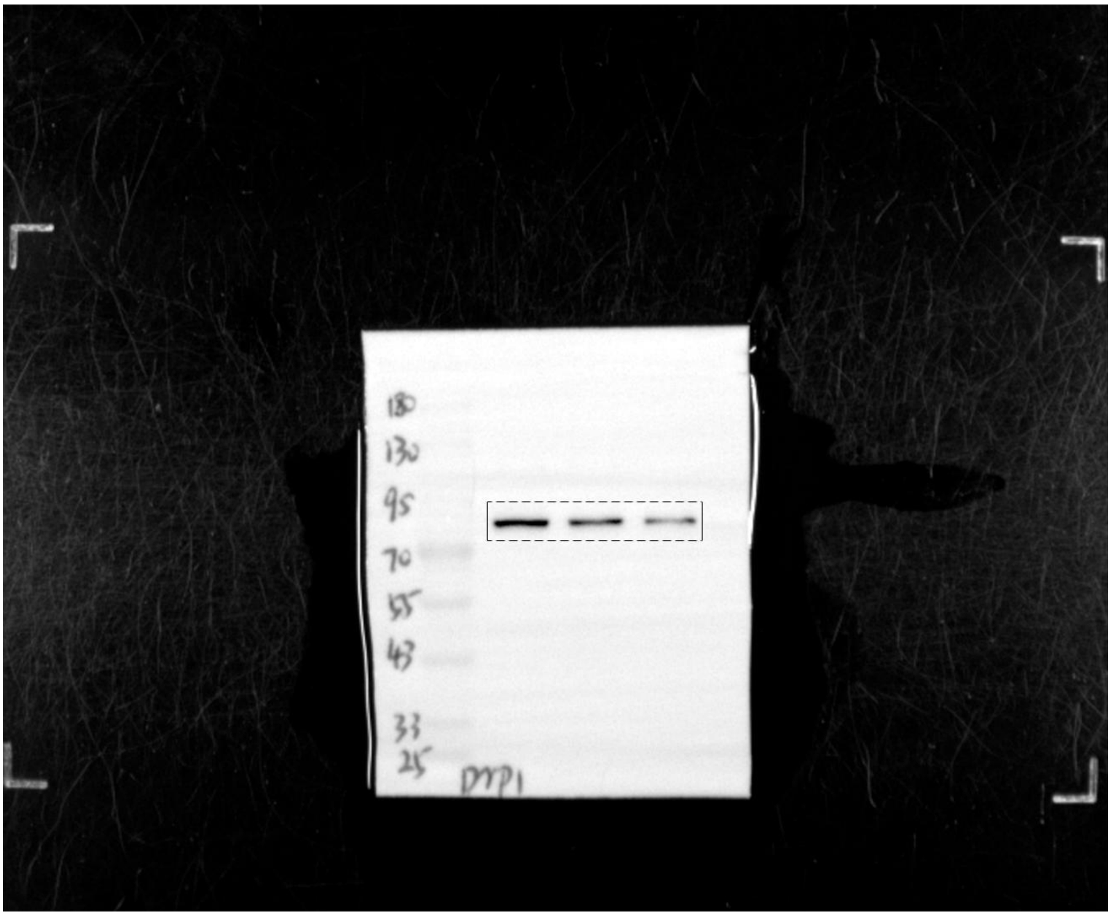

Full unedited gel/blot for Figure 7A-FTO

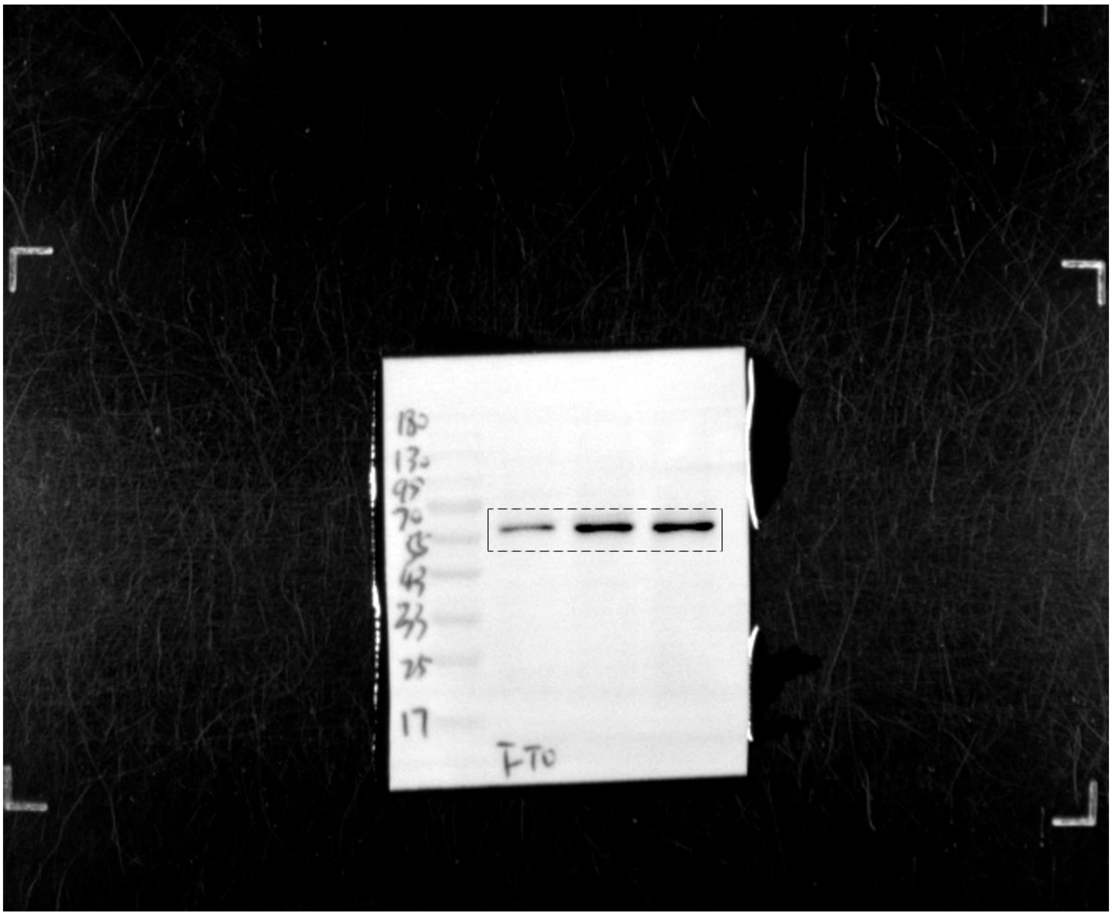

Full unedited gel/blot for Figure 7A-FYN

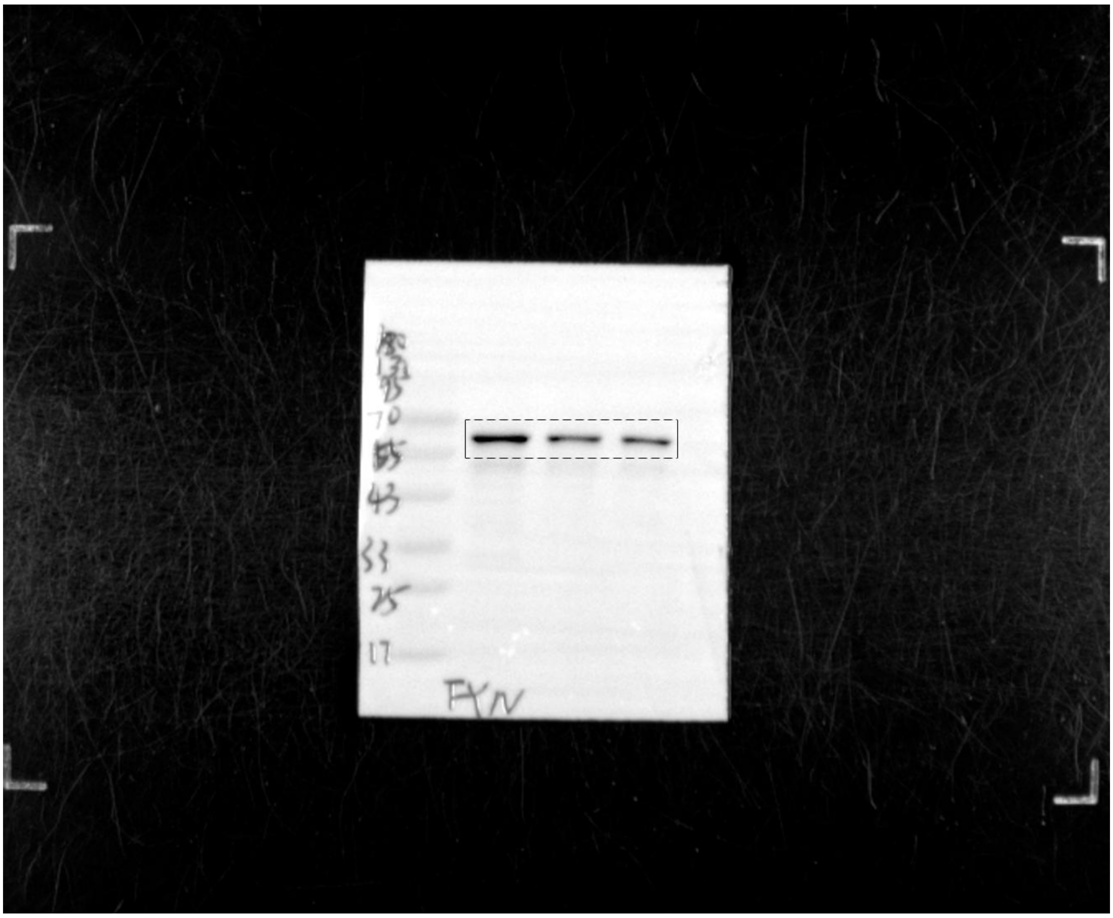

Full unedited gel/blot for Figure 7A-p-Drp1

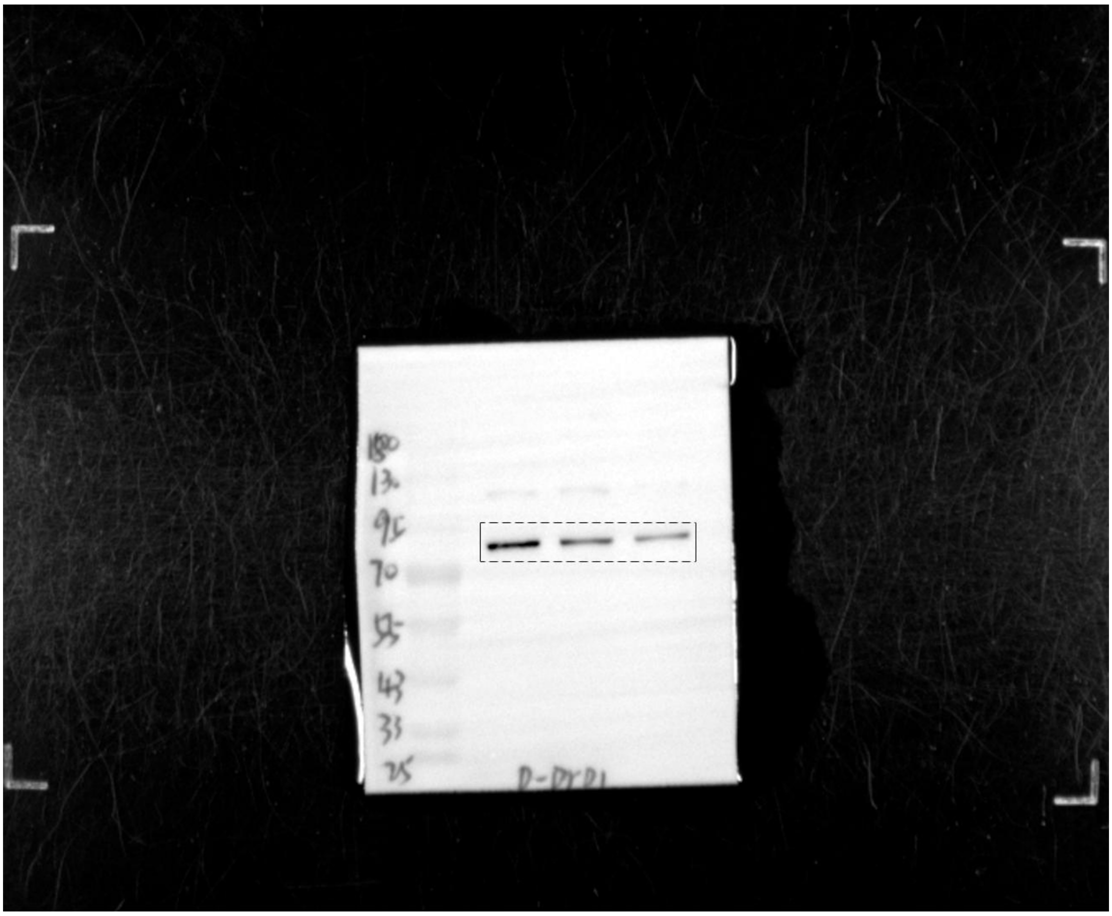

Full unedited gel/blot for Figure 7A-β-actin

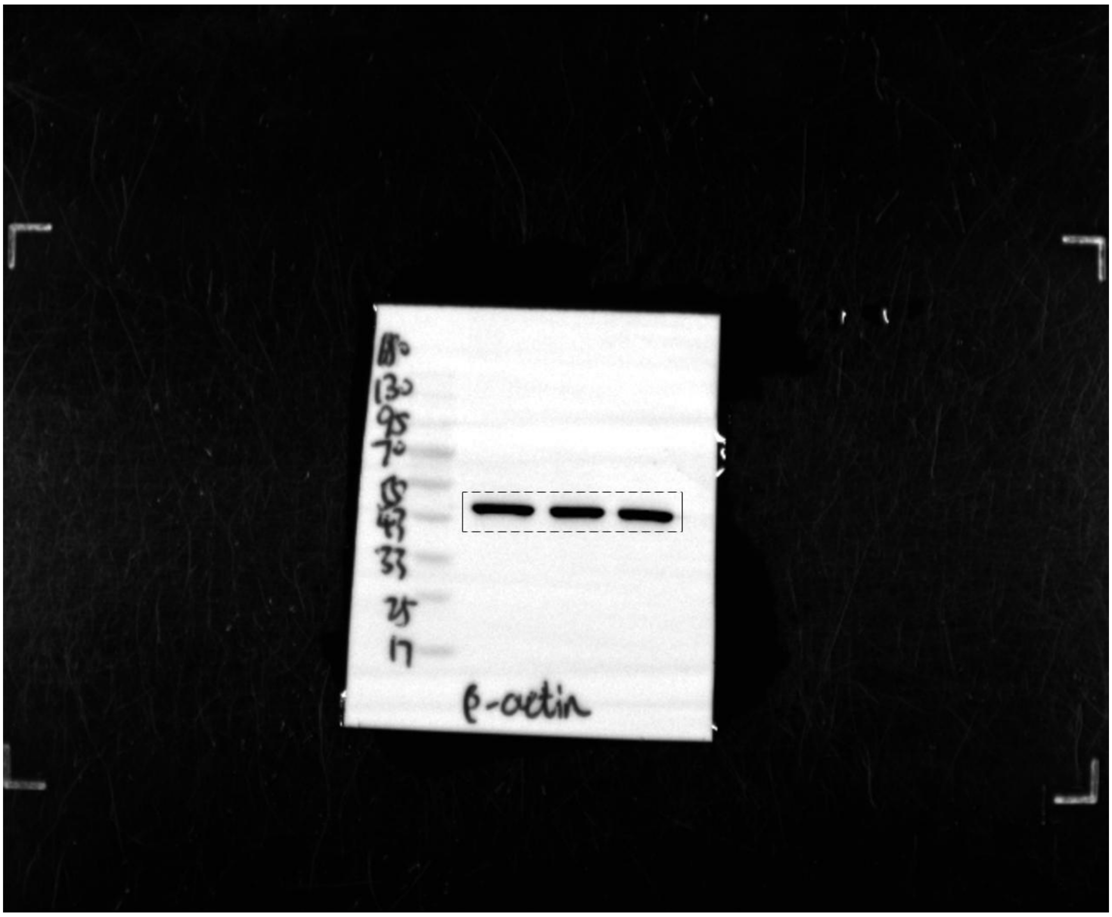

Full unedited gel/blot for Figure 7J-4-HNE

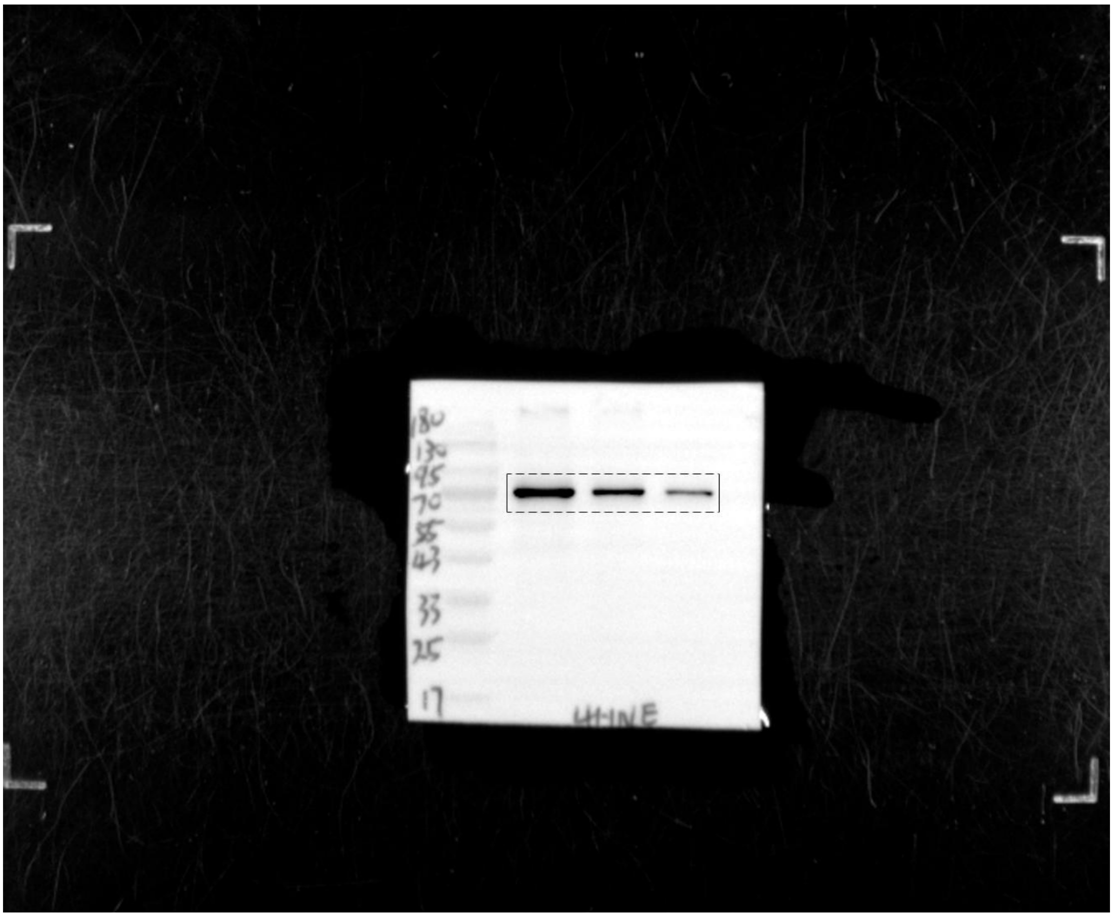

Full unedited gel/blot for Figure 7J-GPX4

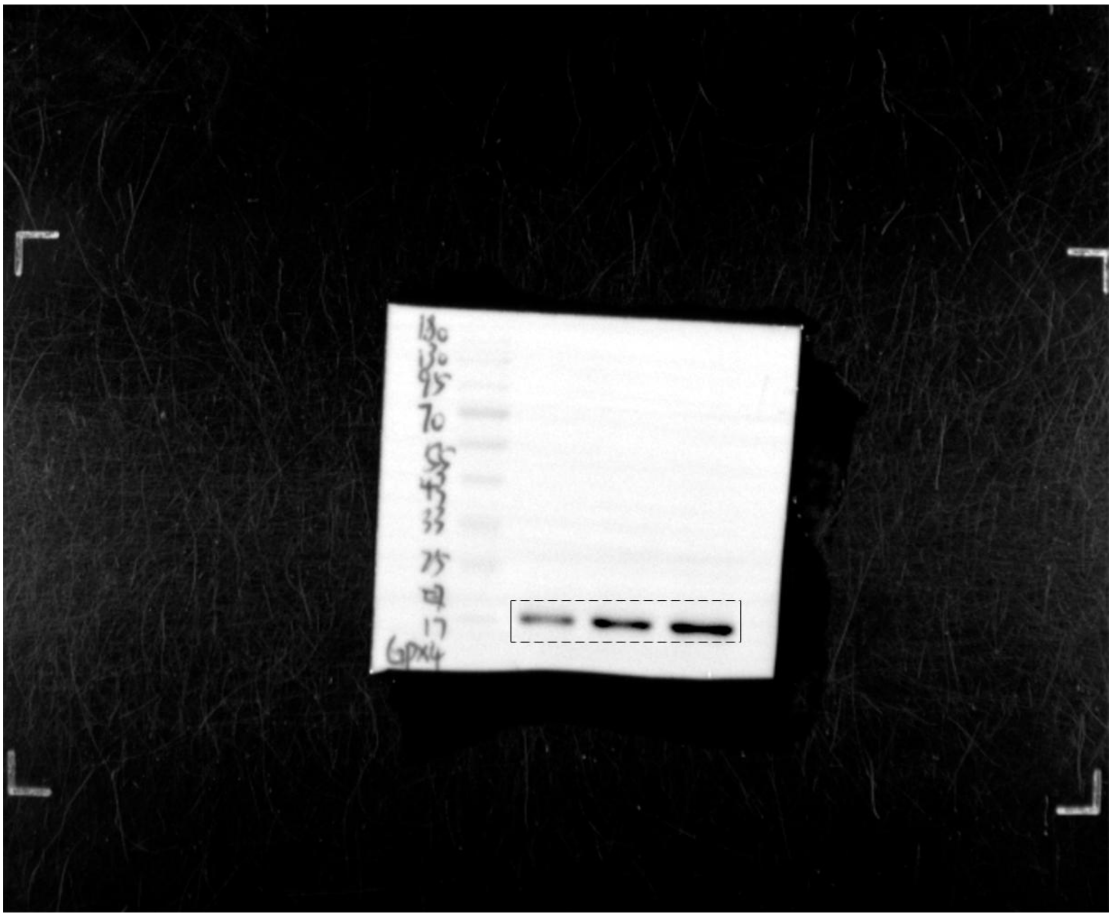

Full unedited gel/blot for Figure 7J-β-actin

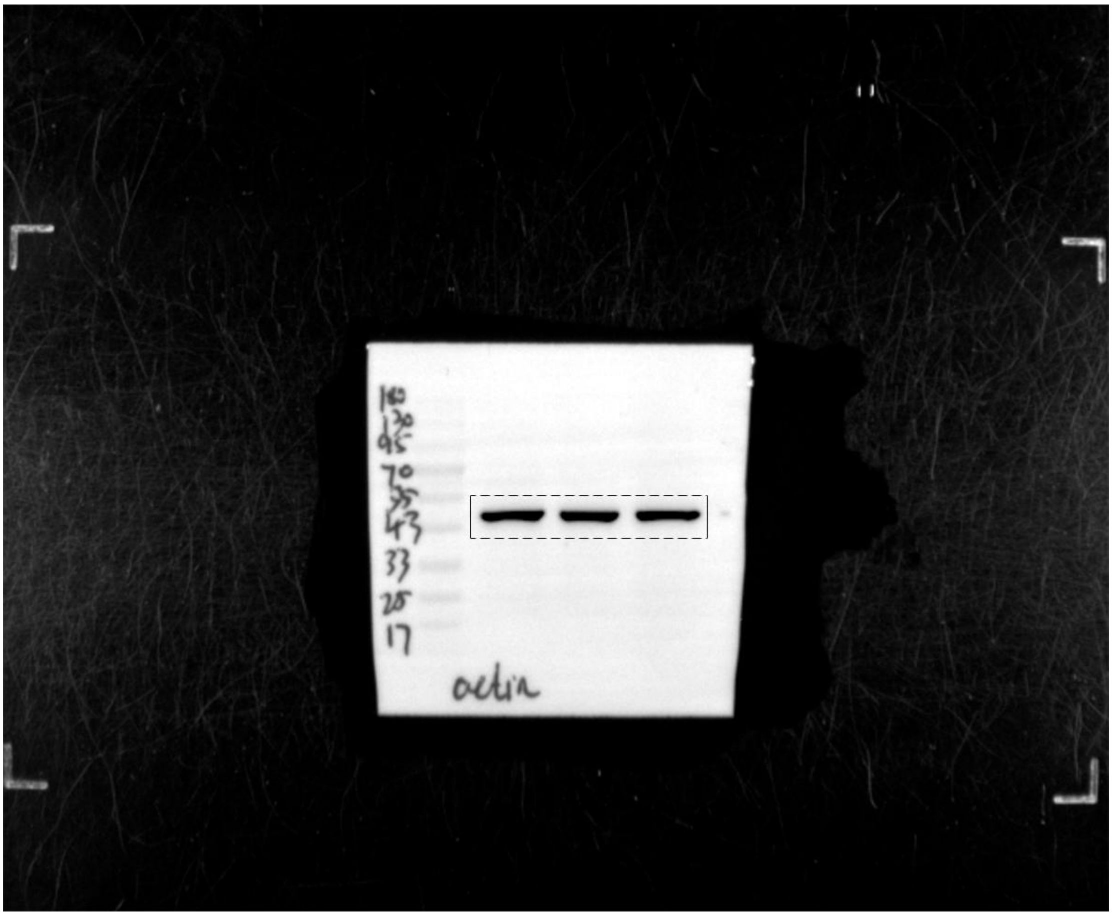

Supplement: Supplementary file 1 — Figures S1–S7 [file CNS-30-e14636-s001.pdf]
